# Synthesis and chemosensing properties of cinnoline-containing poly(arylene ethynylene)s

**DOI:** 10.3762/bjoc.11.43

**Published:** 2015-03-20

**Authors:** Natalia A Danilkina, Petr S Vlasov, Semen M Vodianik, Andrey A Kruchinin, Yuri G Vlasov, Irina A Balova

**Affiliations:** 1 Institute of Chemistry, Saint Petersburg State University, Universitetskiy pr. 26, Saint Petersburg, 198504, Russia. Fax: +7(812)4286733

**Keywords:** cinnolines, fluorescence quenching, Pd^2+^ detection, poly(arylene ethynylene)s, Sonogashira coupling

## Abstract

Novel poly(arylene ethynylene)s comprising a cinnoline core were prepared in high yields via a three-step methodology. A Richter-type cyclization of 2-ethynyl- and 2-(buta-1,3-diynyl)aryltriazenes was used for cinnoline ring formation, followed by a Sonogashira coupling for the introduction of trimethylsilylethynyl moieties and a *sila*-Sonogashira coupling as the polycondensation technique. The fluorescence of the cinnoline-containing polymers in THF was highly sensitive to quenching by Pd^2+^ ions.

## Introduction

Optical sensors provide an efficient and sensitive approach for the rapid detection and identification of a wide range of chemical substrates by measuring the changes of optical properties such as absorbance, reflectance, luminescence, or fluorescence [1]. Among them a type of sensors based on conjugated polymers (CPs) are of crucial importance for biological, environmental and clinical applications because of the remarkably amplified response of CPs to an analyte, compared with the respective monomer unit [2–7]. Since the change in fluorescence is the most sensitive and the most commonly used type of response, conjugated polymers for sensing are termed as amplifying fluorescent polymers (AFPs) [4]. The mechanism of the response amplification can be explained in terms of the "molecular wire" concept [2–9]. Binding of a single analyte molecule to a single recognition site completely quenches the fluorescence of the whole polymer chain, giving the increase of the sensitivity (amplification). This effect is also known as superquenching and comes from the delocalization of the charge carriers in the polymeric structures. Transport of excitons along the polymer backbone in a separate molecule as well as the transport of excitons between adjacent molecules makes possible a non-radiative relaxation of many excitons at a single trap created as a result of an analyte binding to a receptor integrated into the polymer chain [10]. As long as the trap and the generation sites of the exciton are spatially separated, the quenching amplification factor is only limited by the diffusion distance of the exciton. In practice, a single bound analyte molecule can deactivate excitons within 130 neighboring phenylene ethynylene repeating units [4].

Sensors based on CPs can be classified either by the nature of the polymer backbone, e.g., polyphenylenes, polythiophenes, poly(phenylene vinylene)s, poly(phenylene ethynylene)s etc, or by their chemosensing properties [3–4]. The latter depends on the nature of receptors (recognizing units) incorporated into a polymer chain that bind an analyte. Such materials find widespread use in sensing of metal ions and anions, detection of explosives and various biomolecules [2−4]. Among the great variety of known conjugated polymers, poly(arylene ethynylene)s (PAEs) are one of the most promising materials in the search for new chemical sensors [7].

**Figure 1:**
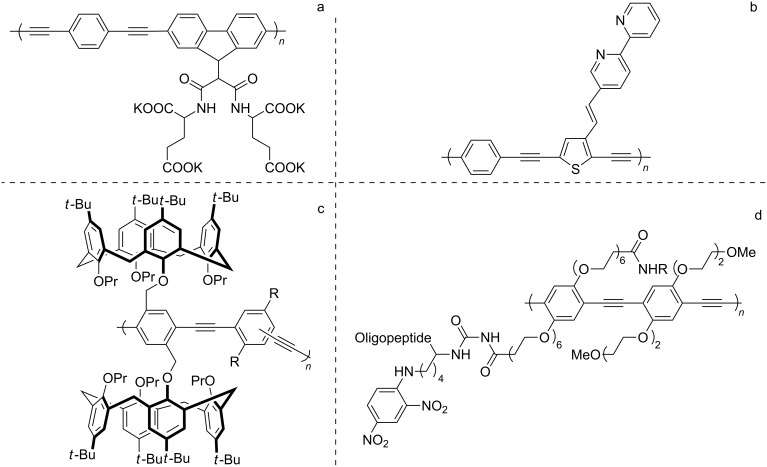
Recent examples of PAEs and their application for the detection of Hg^2+^ (a) [11], Ni^2+^ (b) [12], explosives (c) [13] and trypsin (d) [14].

A wide range of PAEs has been developed in the last decade [2–4], for example polymers with ether, ester or carboxylic acid functions [11] and *N*-containing heterocyclic scaffolds [12] for metal ion sensing; polymers with polycyclic aromatics and rigid groups for the detection of explosives [13]; and PAEs with ionogenic groups and specific functions for different kinds of biosensing [14] (Figure 1).

There are several known examples of PAEs having bi-, tri- and terpyridyl scaffolds that are capable of transition metal ion sensing [12,15] and pyridine-containing polymers for other applications [16–18]. Moreover, various species of` PAEs with quinoline [19–20], quinoxaline [21–22], thiadiazole [17,23], carbazole [18,24], 1,2,4-triazoles [25], thiazole [26] and azametallocyclic [27] units incorporated into a polymer chain have been described.

Since ligands based on pyridazine rings can form complexes with transition metal ions (e.g. Cu^+^, Cu^2+^, Ag^+^, Co^2+^, Ni^2+^) [28–35], the introduction of this structural fragments into PAEs might give rise to a new family of chemosensors. Here we describe the first synthesis of PAEs consisting of altering cinnoline, ethynyl and aryl units and investigate their chemosensing ability towards transition metal ions.

## Results and Discussion

### Synthesis and structure of cinnoline-containing PAEs

**Figure 2:**
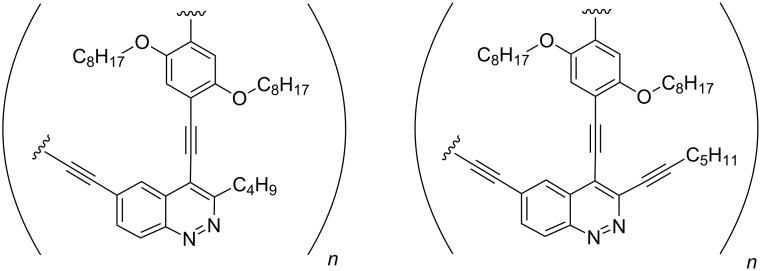
Target structures of PAEs.

Cinnoline-containing PAEs with two types of repeating units that differ by substituents at the C-3 carbon atom of a cinnoline ring (alkyl or alkynyl moiety) were chosen as target structures (Figure 2).

They were chosen in order to study the influence of an additional triple bond on the photochemical properties of cinnoline-containing PAEs. It should be noted that PAEs with an additional ethynyl moiety are almost unknown and only few examples of dimers and trimers were studied to date [36]. The *O-*alkylated hydroquinone scaffold was chosen as a commonly used spacer between the diethynylcinnoline units [2–4] that prevents the aggregation of polymer chains.

An efficient modification of the Richter type cyclization [37–38] based on mono- [39–40] and diacetylene [41–43] aryltriazene derivatives as starting materials was chosen over the approach that uses unstable *o*-ethynyl- or *o*-(buta-1,3-diynyl)arenediazinium salts. This reaction was a key step for the synthesis of cinnoline-containing structural units – 4,6-dibromocinnolines **4a,b** (Table 1).

**Table 1:** Synthesis of 4,6-bis(trimethylsilylethynyl)cinnolines **4**.

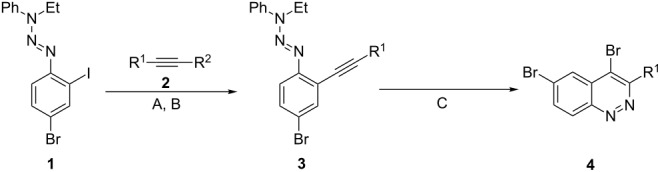							

Entry	R^1^	R^2^	Acetylene	Conditions^a^	Triazene, (yield, %)	Conditions^a^	Cinnoline, (yield, %)

1	C_4_H_9_	H	**2a**	A	**3a** (76)	C	**4a** (87)
2	≡−C_5_H_11_	TMS	**2b**	B	**3b** (90)	C	**4b** (58)

^a^Conditions: A – Pd(PPh_3_)_4_, CuI, Et_3_N, 35 °C; B – Pd(PPh_3_)_4_, CuI, KF, MeOH, DMF, rt; C – aqueous HBr (48%, 20 equiv), acetone, 20 °C.

Triazenes **3a,b** were synthesized from 4-bromo-2-iodophenyltriazene **1** by the Sonogashira coupling [44–45] with hexyne (**2a**) and TMS-protected diacetylene **2b** using conditions for the one-pot TMS group removal and the Sonogashira coupling developed recently [46]. The last reaction could also be defined as a type of *sila*-Sonogashira coupling [47–48], which is an extremely convenient method for the one-step synthesis of non-symmetrically substituted diarylacetylenes [49–50]. Mild conditions allowed carrying out both reactions chemoselectively leaving the bromine atom for further synthetic steps. 4,6-Dibromo-3-butylcinnoline (**4a**) and 4,6-dibromo-3-(hept-1-yn-1-yl)cinnoline (**4b**) were obtained from corresponding triazenes **3a,b** by Richter cyclization under known conditions [43] in satisfactory and good yields.

Although the Sonogashira coupling of 4-bromocinnolines has been recently reported [41,51], the double Sonogashira coupling for dibromocinnolines has not been investigated. To optimize the coupling conditions, several test experiments were carried out using the 4,6-dibromo-3-butylcinnoline (**4a**, Table 2).

**Table 2:** Conditions optimization for the double Sonogashira coupling.

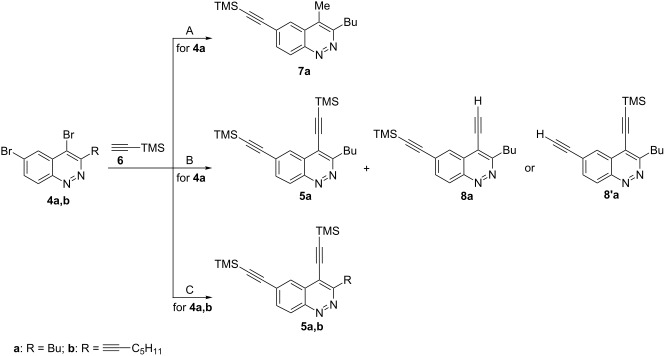				

Entry	Dibromocinnoline	R	Conditions^a^	Reaction product (yield, %)^b^

1	**4a**	Bu	A	**7a** (50)
2	**4a**	Bu	B	**5a:8a (or 8’a)** (34:23)^c^
3	**4a**	Bu	C	**5a**
4	**4a**	Bu	C	**5a** (88)
5	**4b**	≡−C_5_H_11_	C	**5b** (91)

^a^A – trimethylsilylacetylene **6** (5 equiv), Pd(PPh_3_)_4_, CuI, DIPA, DMF, 80 °C, 24 h; B – trimethylsilylacetylene **6** (3 equiv), Pd(PPh_3_)_4_, CuI, Et_3_N, 50 °C, 20 h; C – trimethylsilylacetylene **6** (3 equiv), Pd(PPh_3_)_4_, CuI, Et_3_N, 50 °C, 3.5 h; ^b^Entries 1,2,4,5 – isolated yields, entry 3 – the full conversion of **4a** to **5a** was estimated by ^1^H NMR spectroscopy, the isolated yield of **5a** was not determined; ^c^The distinguishing between **8a** and **8’a** was not done.

Taking into account that the substitution of a bromine atom in Pd-catalyzed coupling typically requires heating to 80–100 °C [45], we first tried heating in DMF at 80 °C over 24 h in the presence of diisopropanolamine (DIPA) as a base (Table 2, entry 1). Under those conditions the reaction yielded an unexpected 4-methylcinnoline **7a** that could be explained in terms of unusual Hiyama coupling [52–54] of trimethylsilylacetylene with the more reactive C-4 carbon atom of the cinnoline ring. The substitution of the bromine atom at C-4 (not at C-6) of the cinnoline ring was proved by an ^1^H NMR NOESY experiment. Switching to Et_3_N as a solvent and a base as well as decreasing the temperature gave a mixture of the desired product **5a** along with the monodesilylated compound **8a** or **8’a** (Table 2, entry 2). Using ^1^H NMR spectroscopy to monitor the reaction allowed figuring out that heating in Et_3_N at 50 °C for 3.5 hours is enough for a full conversion of the starting cinnoline **4a** into the desired bis(trimethylsilylethynyl)cinnoline **5a** (Table 2, entry 3). The optimized conditions were used in the synthesis of bis(trimethylsilylethynyl)cinnolines **5a,b**, so that the starting materials for the synthesis of PAEs were obtained in good isolated yields (Table 2, entries 4 and 5). These experiments revealed that both bromine atoms at pyridazine and benzene rings are highly reactive in the Sonogashira coupling due to the strong activation of the electron deficient nature of the heterocycle.

Having starting compounds **5a,b** in hand, we were ready to carry out the polycondensation with 1,4-diiodo-2,5-bis(octyloxy)benzene (**9**). There are two main reactions of polycondensation that have been reported as synthetic approaches towards PAEs: the acyclic diyne metathesis polymerization (ADIMET) [55] and the Sonogashira coupling [56]. Both of them have their own advantages and drawbacks [57]. The ADIMET approach usually gives products with higher molecular weights and fewer defects than the Pd-catalyzed polycondensation by the Sonogashira reaction. On the other hand, the Sonogashira coupling is more tolerant to different functional groups and allows the polymer structure to be varied significantly. We chose the Sonogashira coupling of diethynylcinnolines and alkylated diiodohydroquinone as a method for the formation of PAE’s backbone.

It also should be mentioned that despite the fact that *sila*-Sonogashira coupling have been widely applied for the synthesis of different acetylenes with aryl- and hetaryliodides, -bromides -tosylates and -triflates [47–50], there are only a few examples where polycondensation was carried out under these conditions [58–60]. In our case this approach seemed to be more convenient than the common Sonogashira coupling, because it allows TMS-protected diethynylcinnolines to be used as a starting material instead of unstable terminal alkynes. It also avoids an extra synthetic step.

**Scheme 1:**
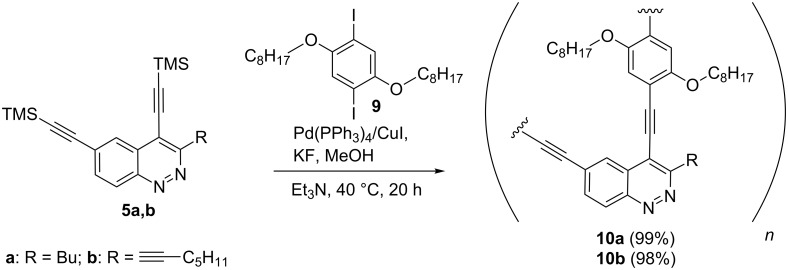
Synthesis of cinnoline-containing PAEs **10a,b**.

When we tried polycondensation of bis(trimethylsilyl)cinnolines **5a,b** with diiodoarene **9** in the presence of Pd(PPh_3_)_4_/CuI as a catalytic system and KF/MeOH as a deprotecting source in Et_3_N, it afforded compounds **10a,b** in quantitative yields even at 40 °C (Scheme 1).

Both products were readily soluble in CHCl_3_, DCM, THF, and MeCN. This allowed for a convenient removal of copper and palladium ion traces by successive washing of the DCM solutions of the products **10a,b** with dilute aqueous ammonia. It also allowed us to determine the molecular mass using gel-permeation chromatography (GPC). The GPC analysis showed mass-average molecular weights (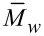) of 13.3 kDa (**10a**) and 9.2 kDa (**10b**), mass average degree of polymerization (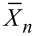) of 9 (**10a**) and 6 (**10b**) and molar-mass dispersity (*Đ*_M_ = 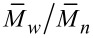) of 2.5 for both compounds. In accordance with this data, compounds **10a** and **10b** should be defined as oligomers. However the common accepted abbreviation for this class of compounds is PAEs (poly(arylene ethynylene)s). This name has also been applied before to compounds with a similar range of mass-average molecular weights (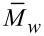) [17,19,25]. Therefore in order to avoid using of new unusual terms the oligomers obtained were named PAEs.

The structure of oligomers **10a,b** was confirmed by ^1^H and ^13^C NMR spectroscopy, FTIR spectra and CHN-elemental analysis. In FTIR spectra of compounds **10a,b** the specific bands for disubstituted triple bonds (R−C≡C−R) stretching at ~2200 cm^−1^ are observed whereas there are no bands of stretching vibration for monosubstituted triple bonds Ar−C≡C−H (≈2100 cm^−1^) and Ar−C≡C−TMS (≈2150 cm^−1^) [61].

The results of the elemental analysis were in agreement with the oligomer structures possessing alkylated iodohydroquinone units as the end groups. The presence of iodine in oligomers was also confirmed by the Beilstein test [62].

**Figure 3:**
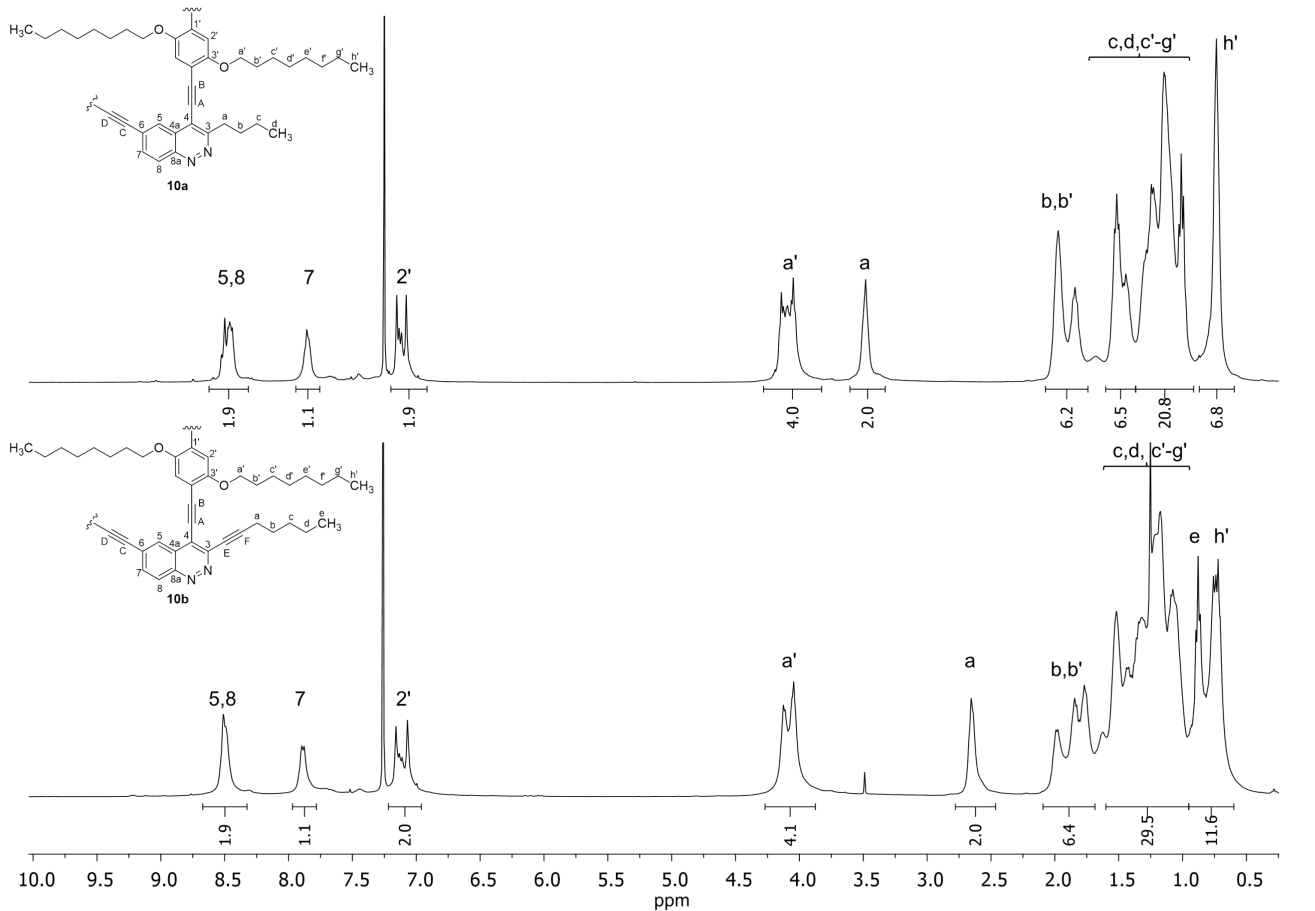
^1^H NMR spectra of PAEs **10a,b** solutions in CDCl_3_.

Regarding the NMR data, the chemical shift values of H and C atoms in both PAEs synthesized do not significantly differ from the chemical shift values in staring cinnolines **5a,b** and diiodoarene **9** that allowed all ^1^H and ^13^C signals in NMR spectra of PAEs to be assigned based on 1D and 2D NMR spectra of starting compounds **5a,b**, **9** and 1,4-diethynyl-2,5-bis(octyloxy)benzene (for copies of all NMR spectra see Supporting Information File 1). Moreover, all signals in ^1^H and ^13^C NMR spectra are rather sharp that corresponds to the oligomer structure of both PAEs **10a,b**. In ^1^H NMR spectra of both compounds **10a,b** the ratio of integral intensities within signals of H atoms from cinnoline (aromatic and alkyl chain hydrogen atoms) and aromatic and alkyl pendant groups of hydroquinone structural units matches the oligomers structure (Figure 3).

**Figure 4:**
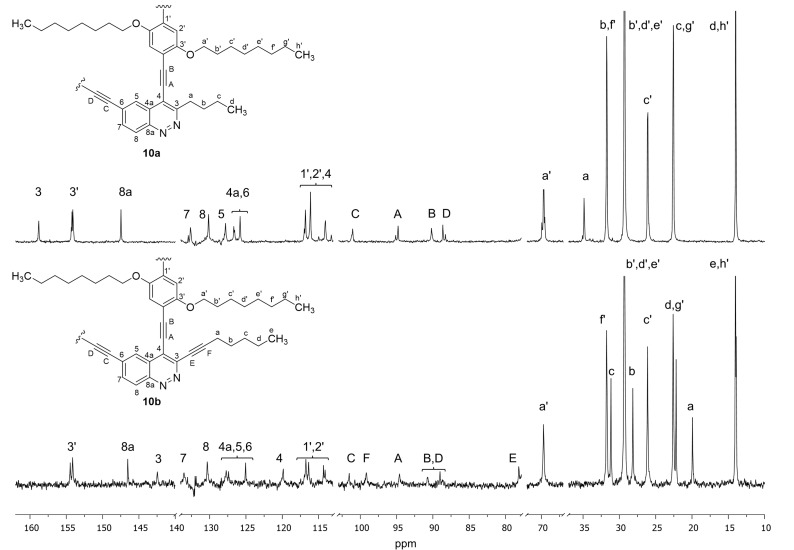
^13^C NMR spectra of PAEs **10a,b** solutions in CDCl_3_.

**Figure 5:**
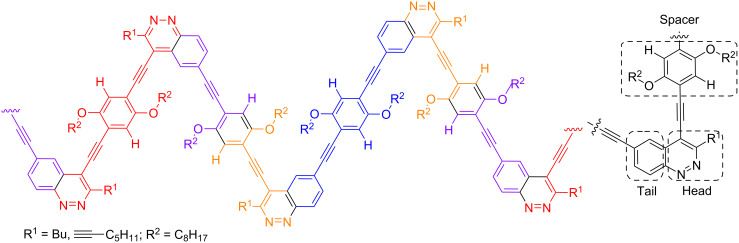
Irregular chain structure (nonequivalent structural units are marked in different colors).

The ^13^C NMR spectra also confirm the presence of both units in PAE molecules **10a,b**. It is important to note that there are four types of C_sp_ atom signals (A–D) in the spectrum of PAE **10a**, which come from the oligomer chain while in the ^13^C NMR spectrum of PAE **10b** two additional C_sp_ signals (E, F) of ethynyl pendant group are observed (Figure 4). The splitting of H atoms (2', a') and C atoms (1'–3', a') signals of hydroquinone structural units of PAEs **10a,b** in NMR spectra confirmed that the orientation of cinnoline units in both oligomer chains is not regular. Thus there are three types of hydroquinone spacers between head-to-head (red), tail-to-tail (blue) and head-to-tail (orange–purple) oriented cinnoline units in the irregular PAEs chains (Figure 5). Moreover for the hydroquinone moiety that connects head-to-tail oriented cinnoline units (orange–purple) the additional splitting was observed (for zoom copies of NMR spectra see Supporting Information File 1).

### Optical properties and metal-sensing ability of cinnoline-containing PAEs

**Figure 6:**
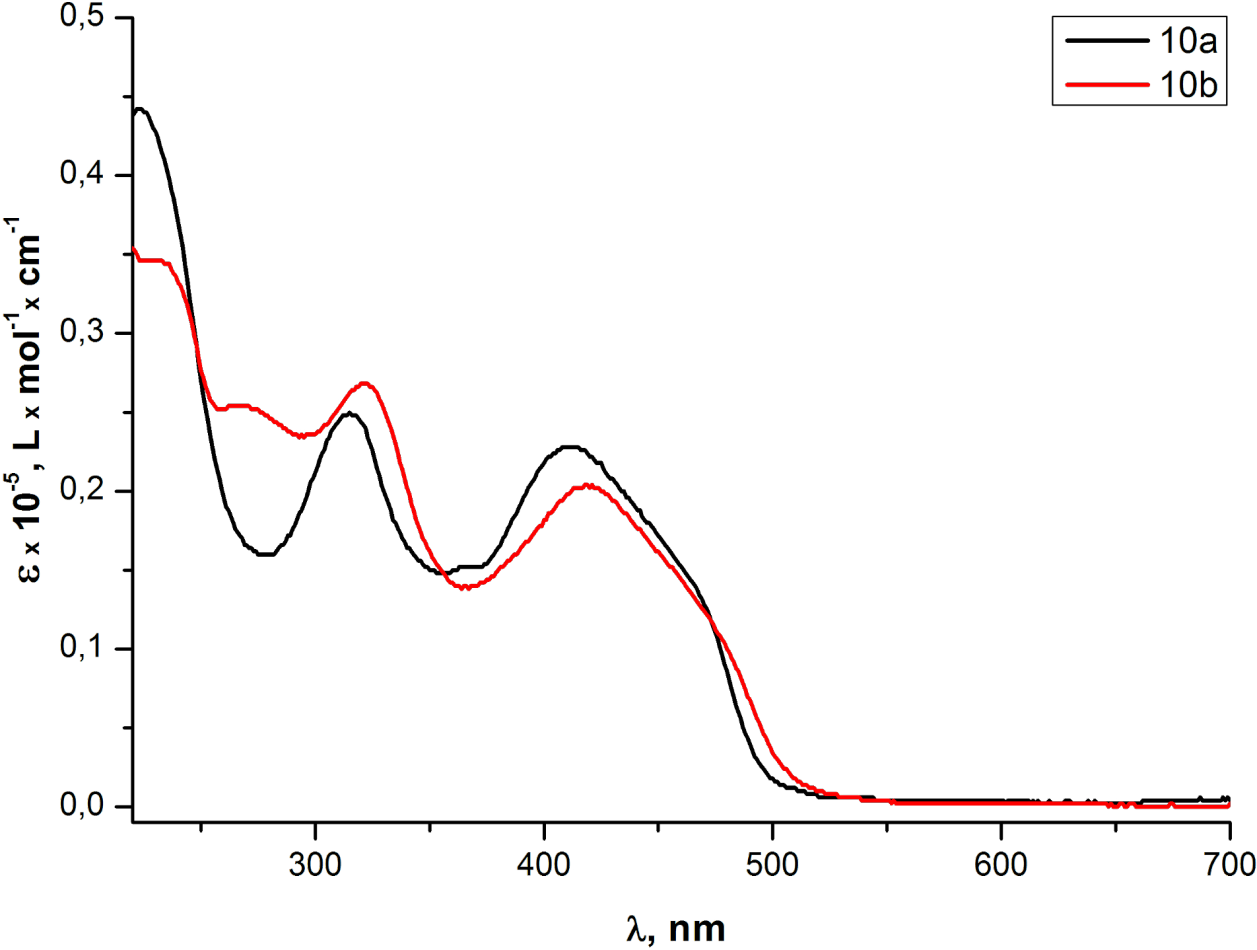
Optical absorption spectra of PAEs **10a,b** in THF solutions.

**Figure 7:**
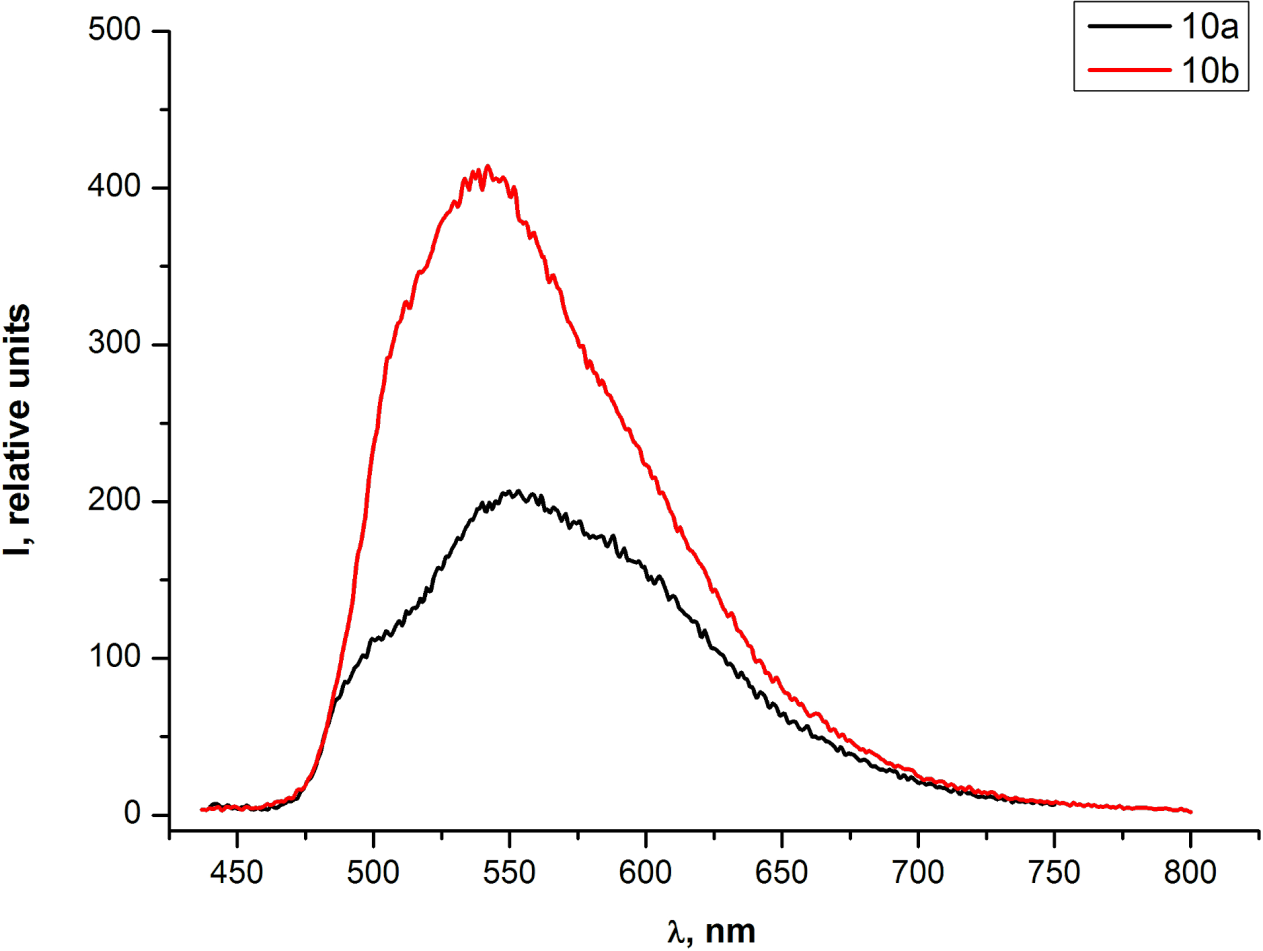
Emission spectra of PAEs **10a,b** in THF solutions.

The absorption spectrum in the UV–vis range of THF solutions of the PAE **10b** with an additional acetylene fragment differed from the absorption spectrum of the PAE **10a** by a small bathochromic shift (≈7 nm) of two absorption bands and an additional absorption band at 266 nm (Figure 6). The fluorescence emission spectra of the same solutions (Figure 7) were recorded at the optimal excitation wavelength of 425 nm which corresponds to the absorption bands at ≈425 nm in UV–vis spectra of both samples (Figure 6). An additional ethynyl moiety in the repeating unit of olygomer **10b** led to the increase in quantum yield from 1% for **10a** to 2% for **10b,** while qualitative similarity of both emission spectra remained unchanged.

**Figure 8:**
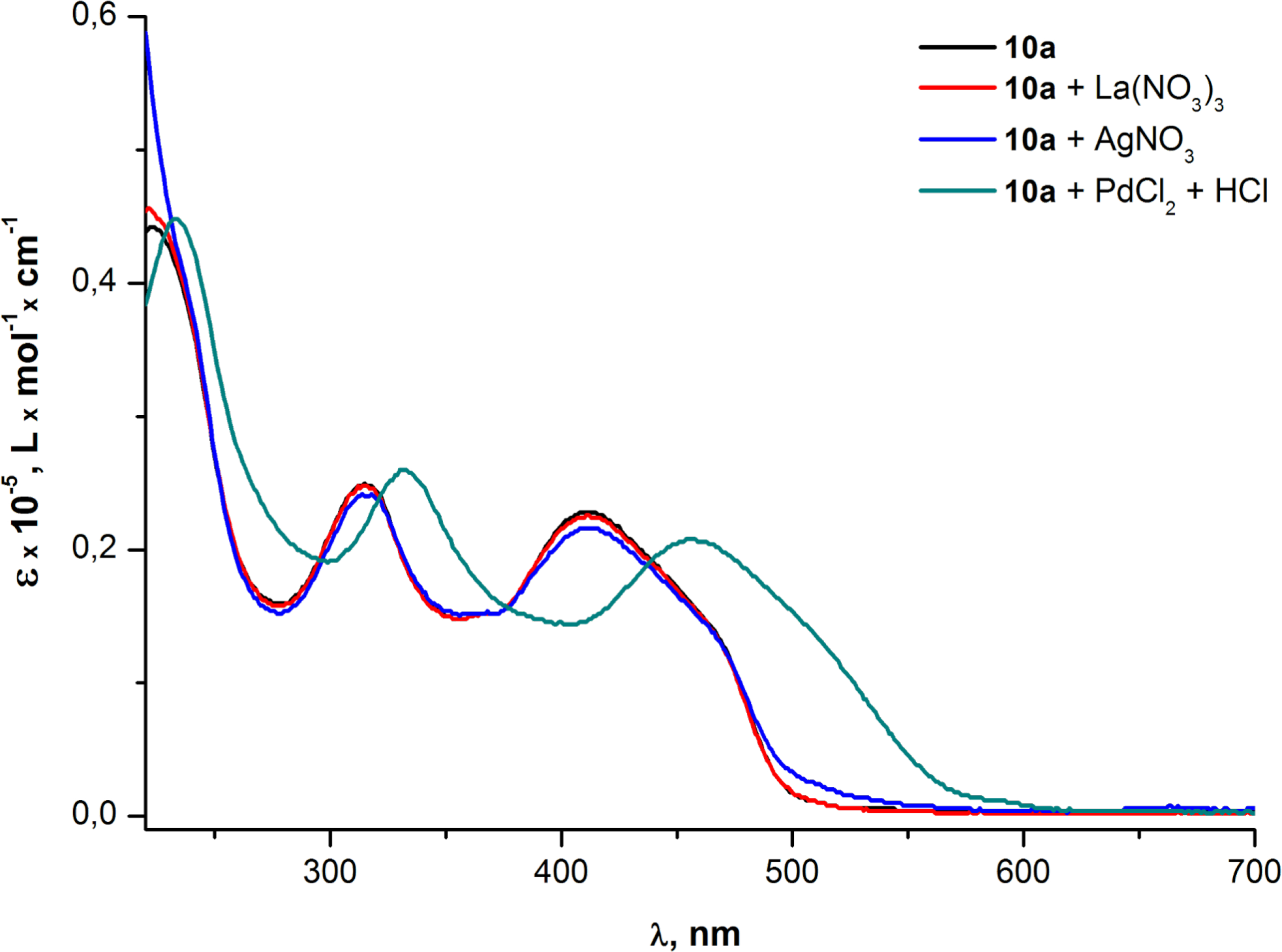
Optical absorption spectra of PAE **10a** in THF before and after the addition of metal analytes.

**Figure 9:**
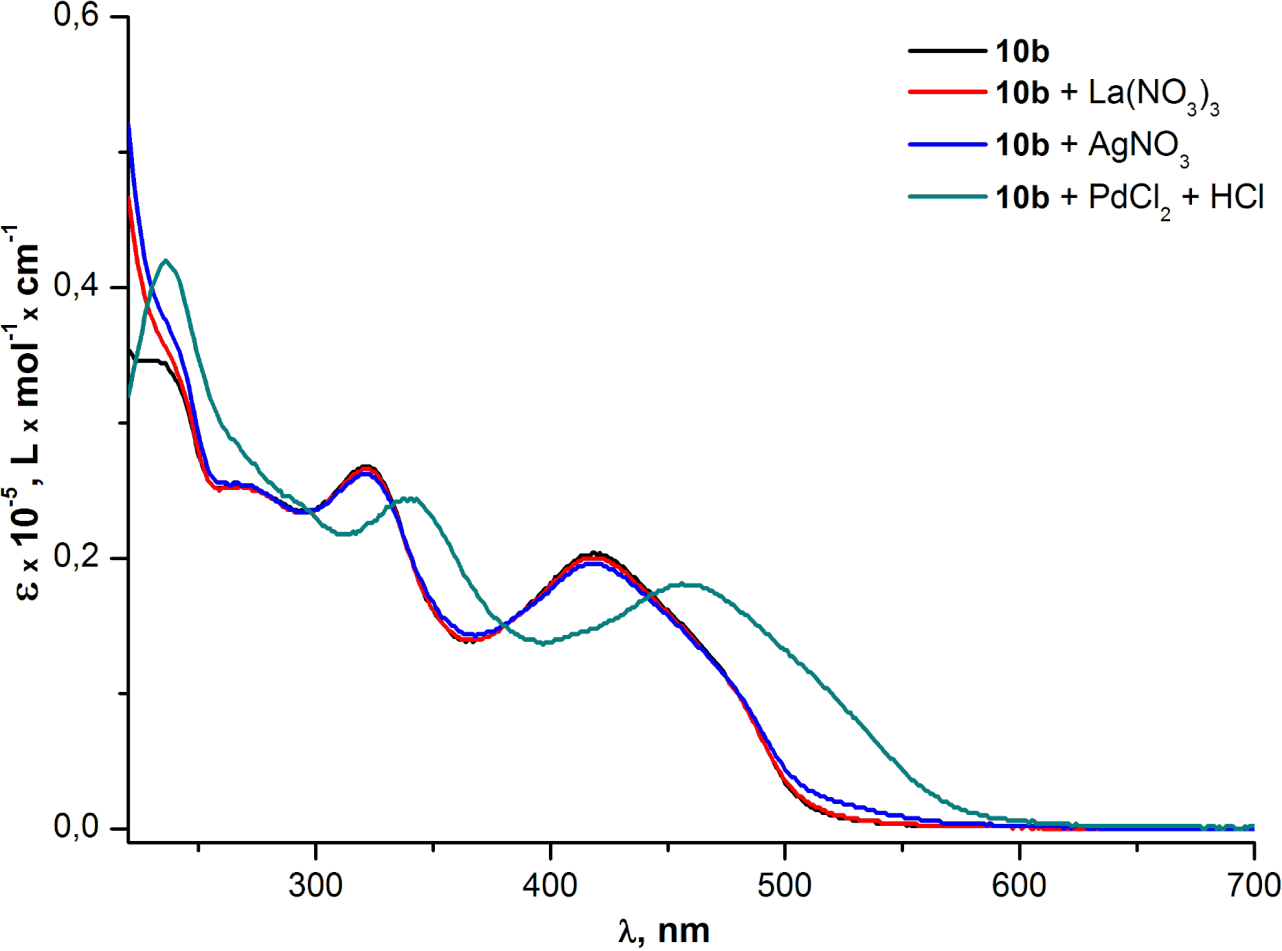
Optical absorption spectra of PAE **10b** in THF before and after the addition of metal analytes.

Then the metal-sensing ability of the PAEs **10a,b** was studied by the screening of the influence of different transition metal ions (Pd^2+^, La^3+^, Ag^+^) on the UV–vis absorption of THF solutions of oligomers. A significant red shift of all absorption bands was observed after the addition of a PdCl_2_/HCl solution to both PAE samples. The influence of Ag^+^ and La^3+^ ions was much weaker and almost did not change the absorption spectra at all (Figure 8 and Figure 9).

**Figure 10:**
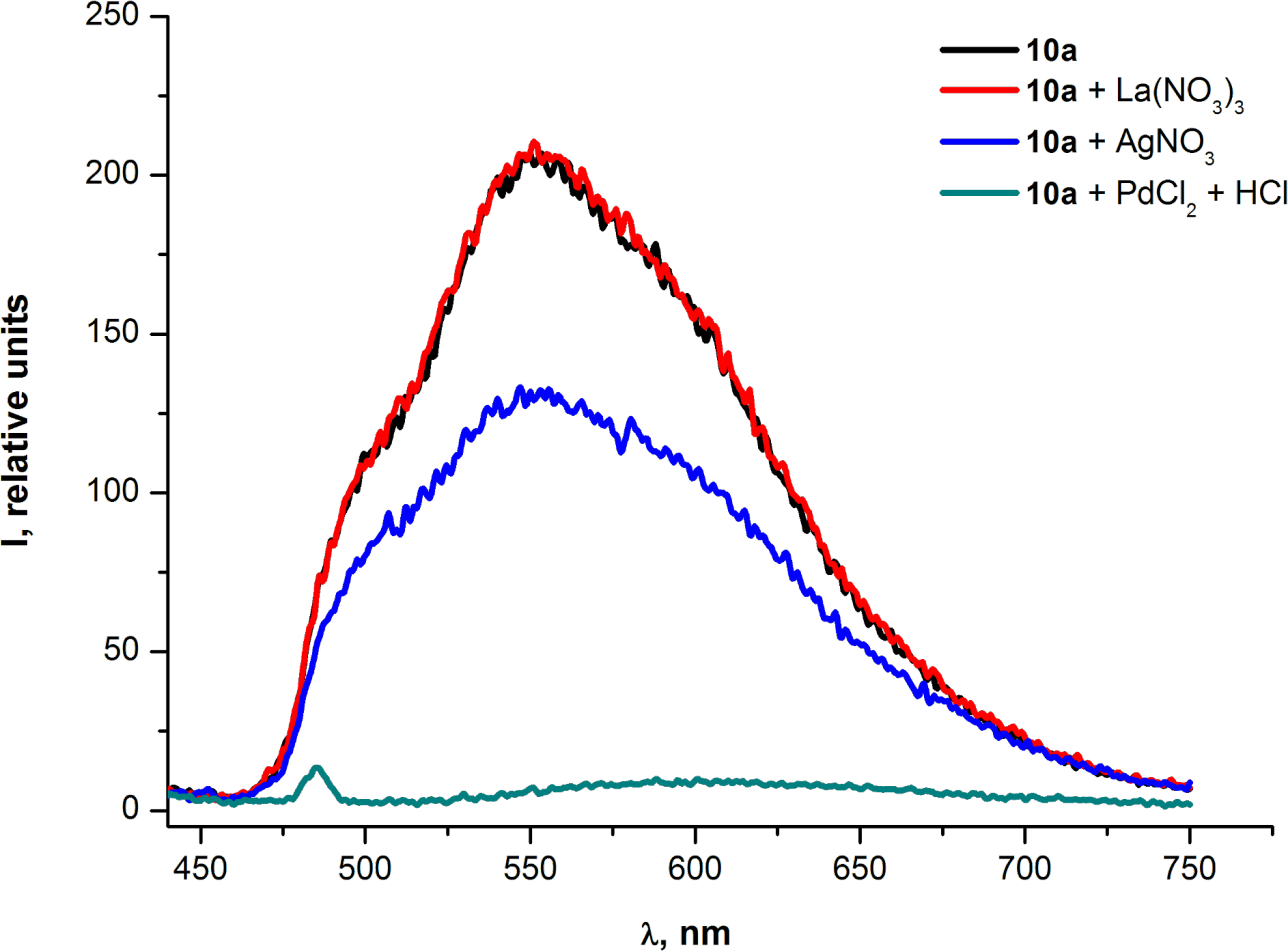
Emission spectra of PAE **10a** in THF before and after the addition of metal ions.

**Figure 11:**
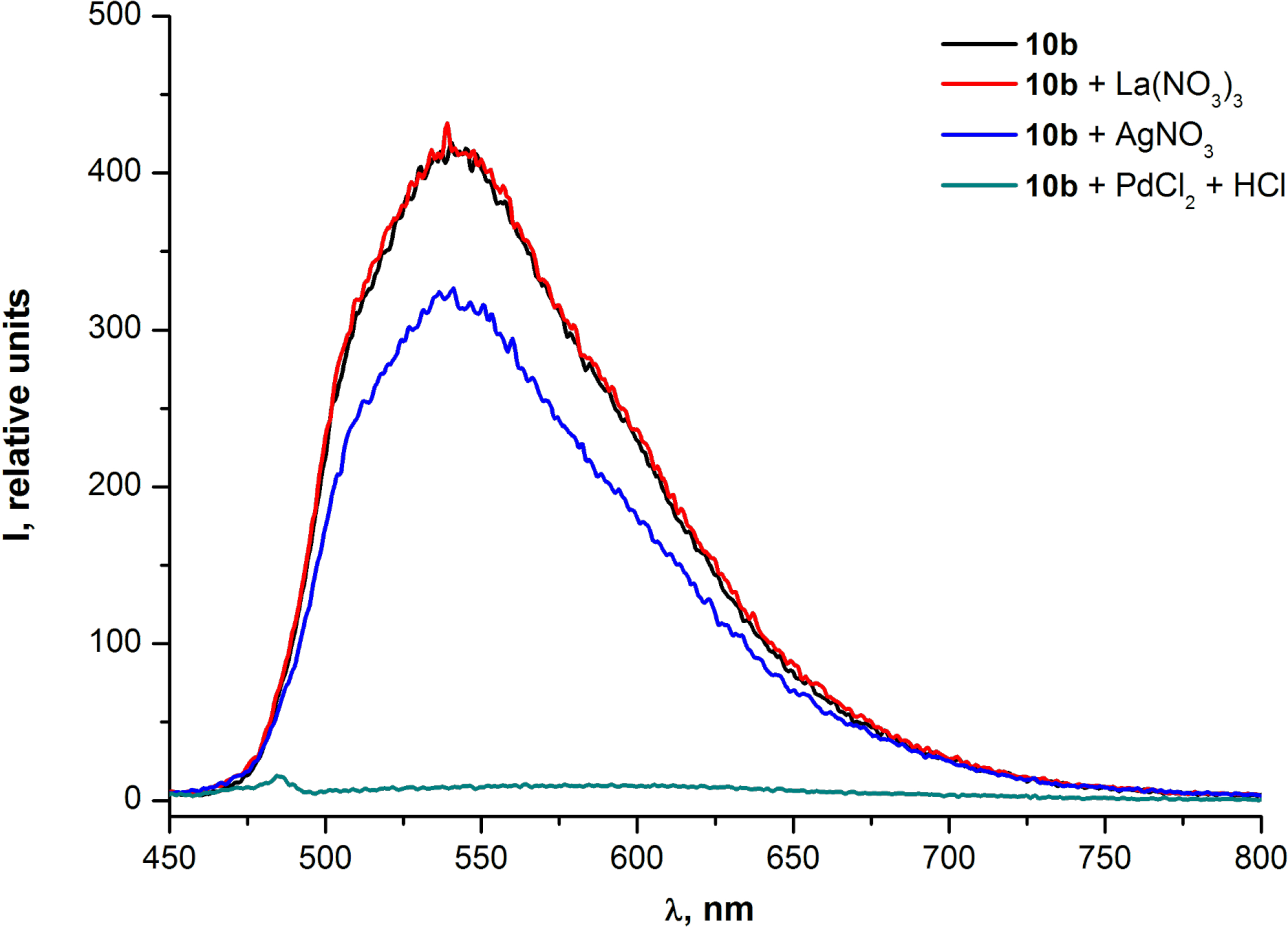
Emission spectra of PAE **10b** in THF before and after the addition of metal ions.

It was also found that Pd^2+^ cations noticeably quenched the fluorescence of oligomers **10a,b** (Figure 10 and Figure 11). For quantitative evaluation of the fluorescence quenching the parameter *Q* was used (*Q = I_o_/I – 1*, where *Q, I_o_* and *I* are the quenching efficiency, the initial fluorescence intensity and the fluorescence intensity after the interaction with a quencher, respectively).

Figure 8 and Figure 9 illustrate that changes in the absorption spectra of compounds **10a,b** resulting from the interaction of oligomers with Pd^2+^ are similar for both samples. The similarity means that neither the additional ethynyl moiety of the repeating unit of **10b** nor the degree of polymerization (*n* = 9 for **10a** and *n* = 6 for **10b**) contribute considerably to the interaction between the palladium and oligomer molecules in the ground (non-excited) state. However, at the same time, the fluorescence quenching efficiency exhibited almost a two fold increase for the PAE **10b** (*Q* = 47) in comparison with the PAE **10a** (*Q* = 26). This illustrates the significant contribution of the aforementioned triple bond to the interaction of Pd^2+^ with oligomer molecules in the excited state (Figure 10 and Figure 11). Moreover, the increased quenching efficiency of the PAE **10b** with shorter chains and an additional ethynyl moiety in the cinnoline ring means that the conjugation extension in the repeating unit that comes from the triple bond is more important for the collective quenching effect than the degree of polymerization.

**Figure 12:**
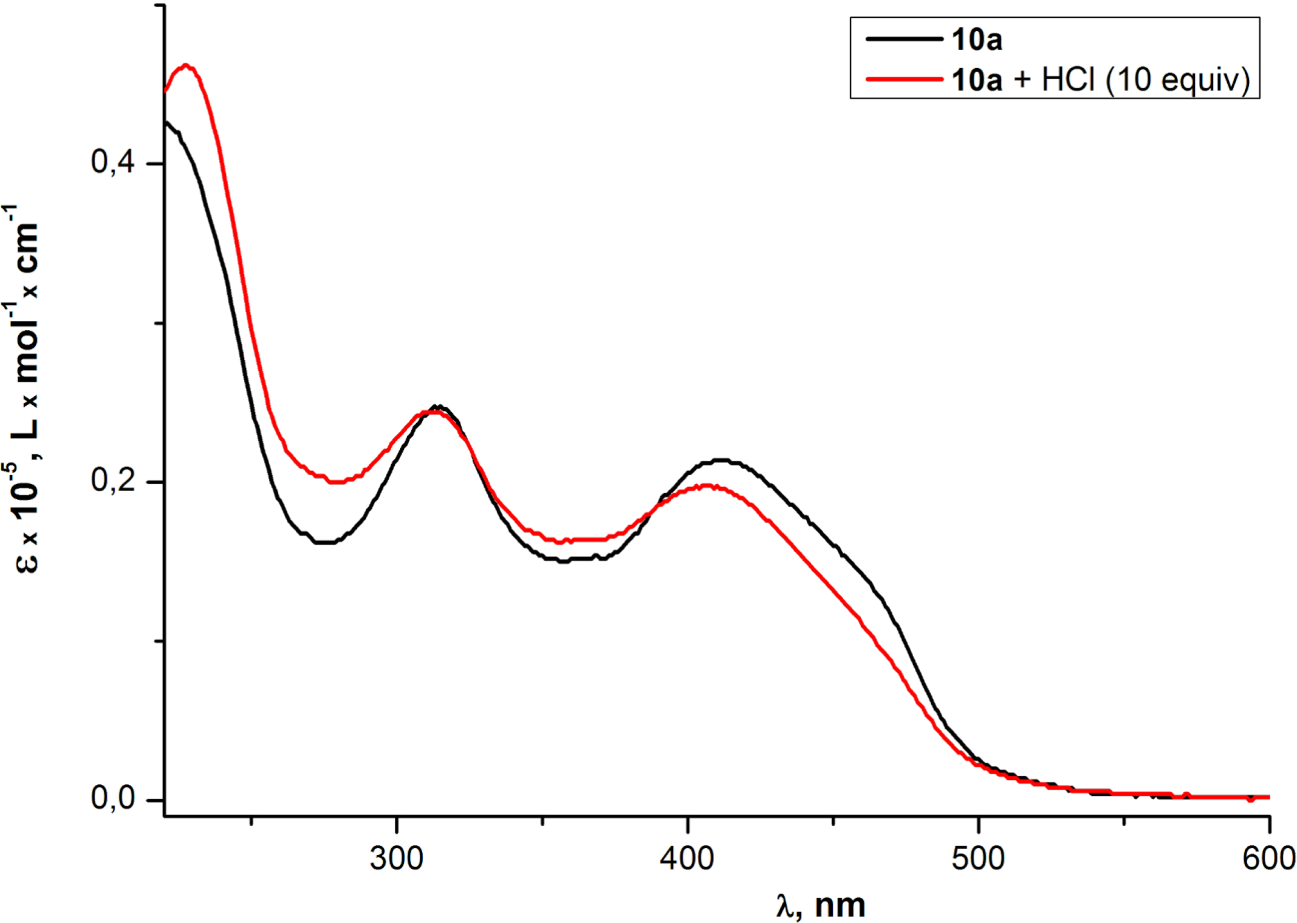
Optical absorption spectra of PAE **10a** in THF before and after the addition of HCl (10 equiv).

**Figure 13:**
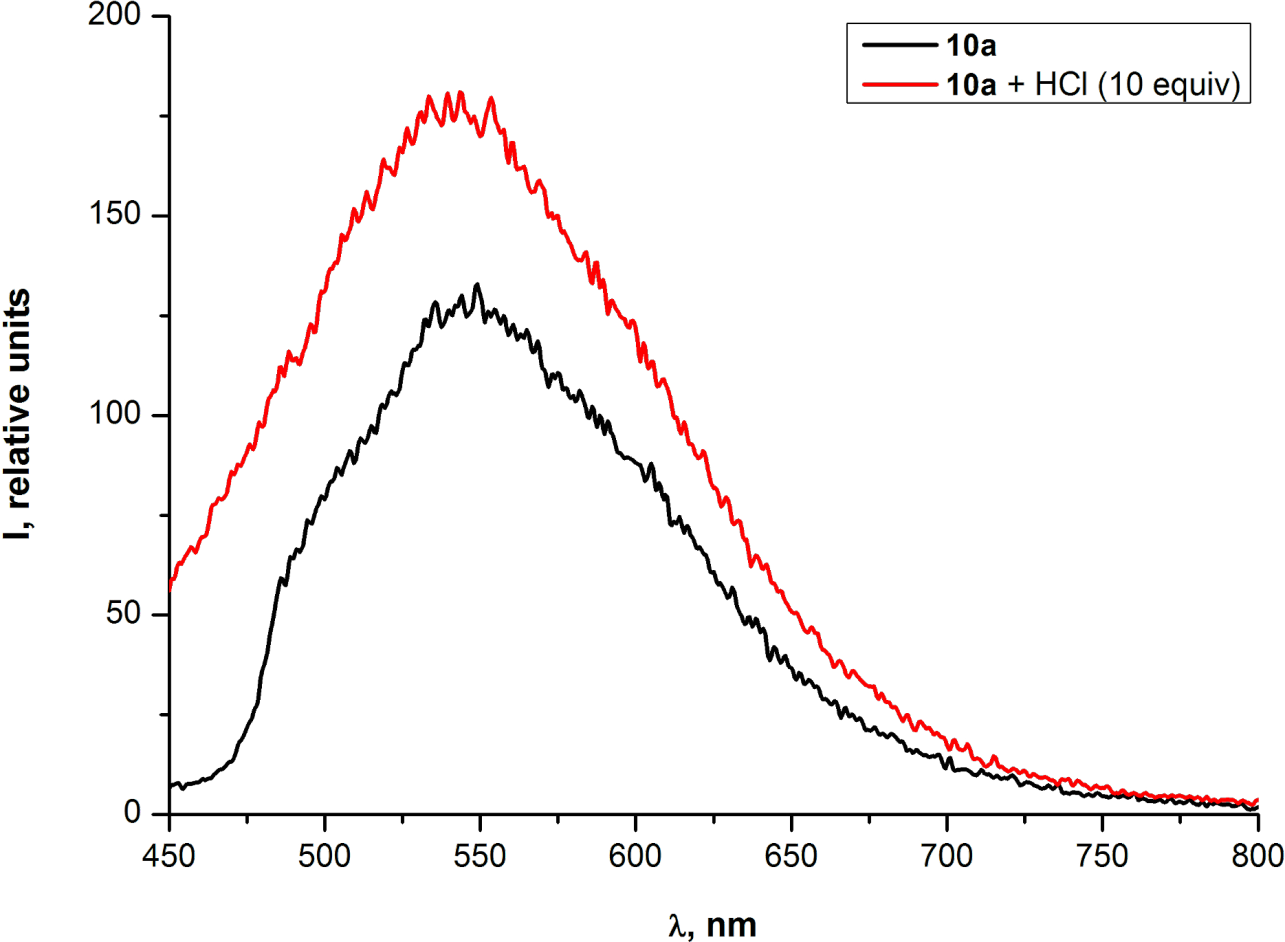
Emission spectra of PAE **10a** in THF before and after the addition of HCl (10 equiv).

In order to prove that the observed changes in optical properties of PAEs **10a,b** were induced by Pd^2+^ and are not related to the hydrochloric acid which was essential for the preparation of an aqueous solutions of PdCl_2_, the influence of HCl on the optical properties of the oligomer **10a** was studied. The obtained data revealed that changes in the absorption and fluorescence emission spectra for the PAE **10a** under 10-fold excess of HCl are opposite to the ones induced by Pd^2+^ ions. Thus instead of a "red" shift of absorption bands and the fluorescence quenching, a slight "blue" shift and the increase in the fluorescence emission were observed (Figure 12 and Figure 13).

**Figure 14:**
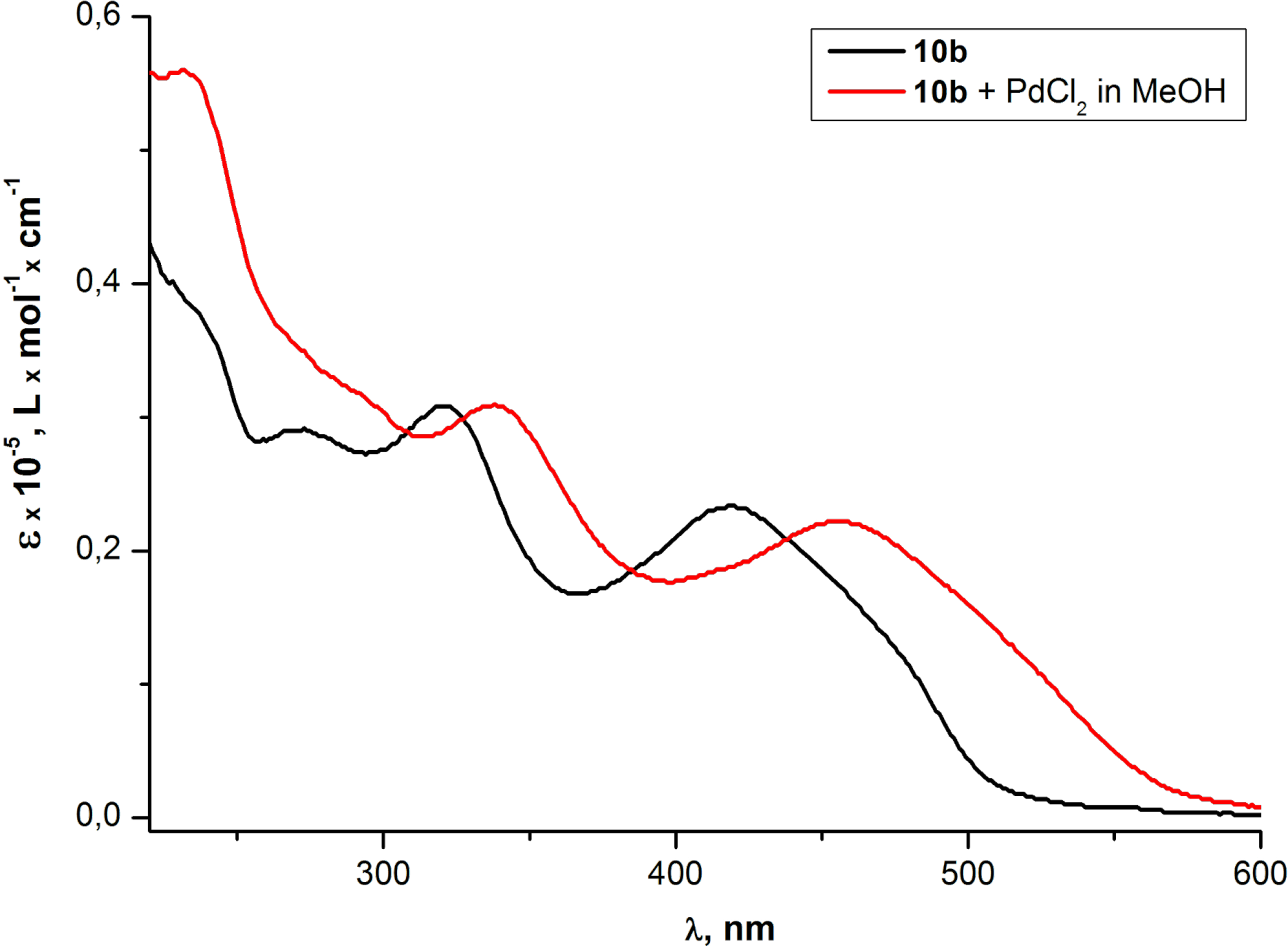
Optical absorption spectra of PAE **10b** in THF before after the addition of methanol solution of PdCl_2_.

**Figure 15:**
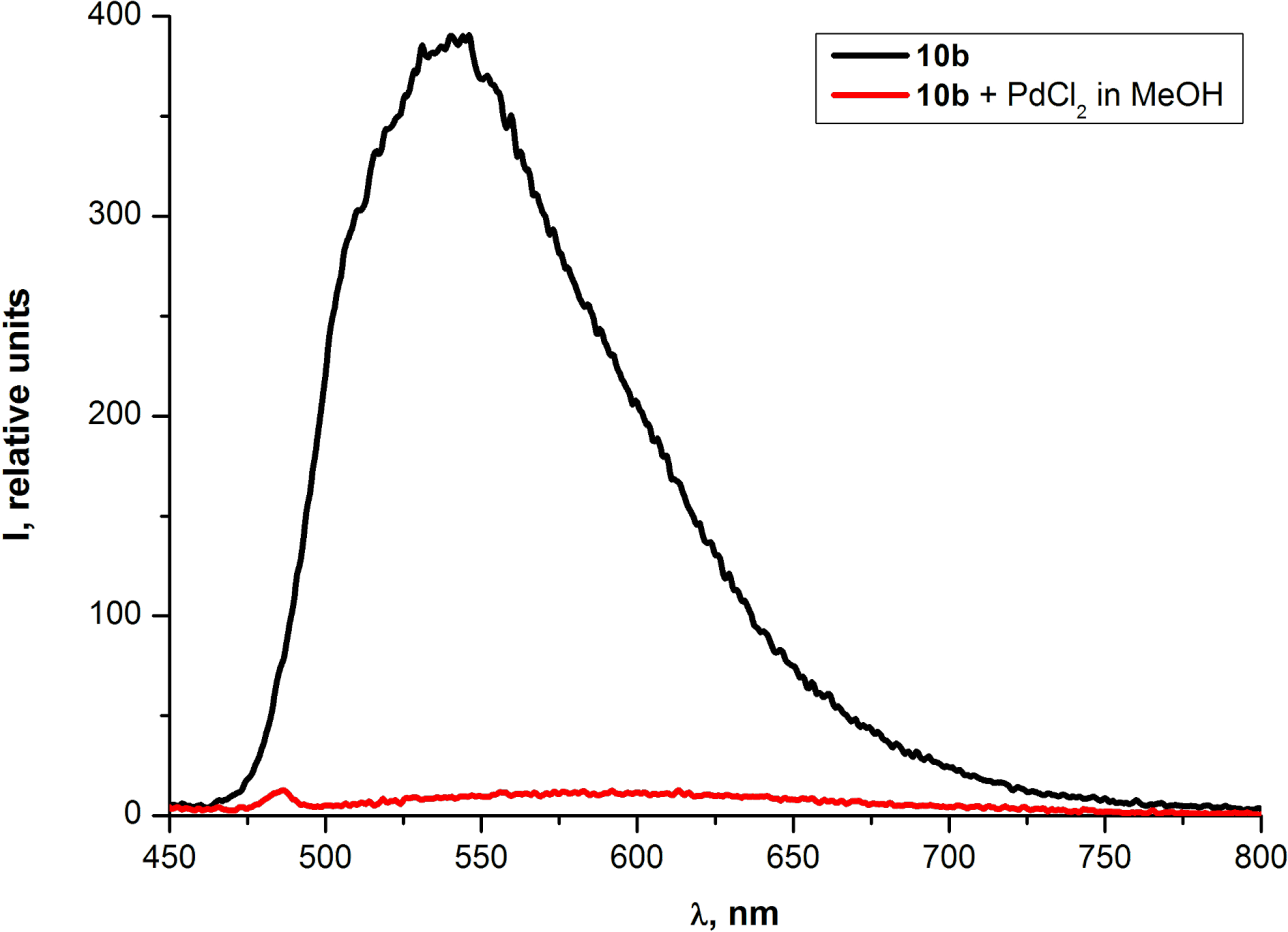
Emission spectra of PAE **10b** in THF before and after the addition of methanol solution of PdCl_2_.

The additional evidence for the Pd^2+^-promoted changes in the absorption and the fluorescence of PAEs **10a,b** was obtained by measuring the UV–vis and fluorescence emission spectra for the THF solution of **10b** in the presence of a methanol solution of PdCl_2_ (Figure 14 and Figure 15). Thus in both spectra changes similar to the ones depicted in Figure 9 and Figure 11 were observed.

**Figure 16:**
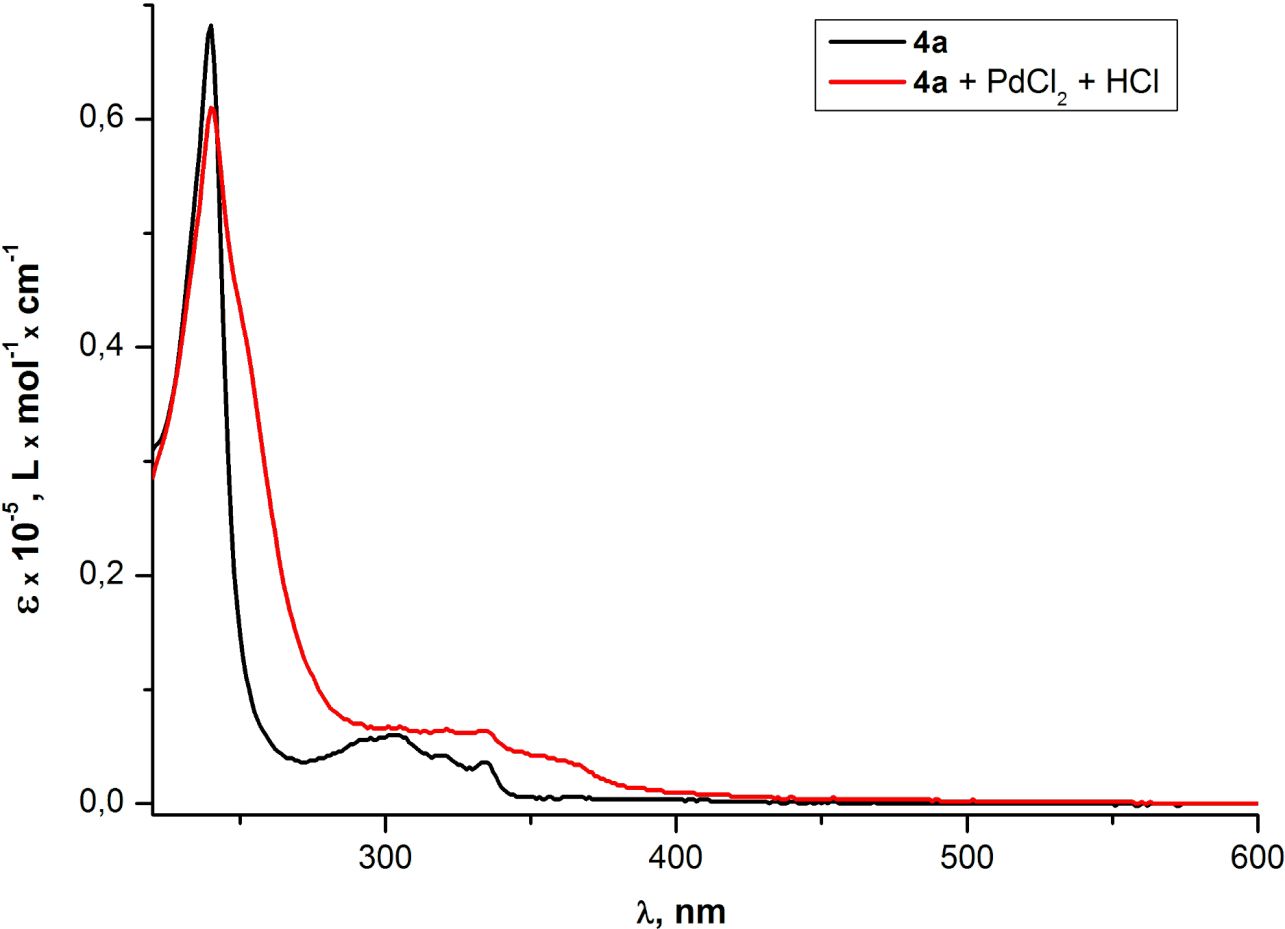
Optical absorption spectra of cinnoline **4a** in THF before and after the addition of aqueous solution of PdCl_2_.

**Figure 17:**
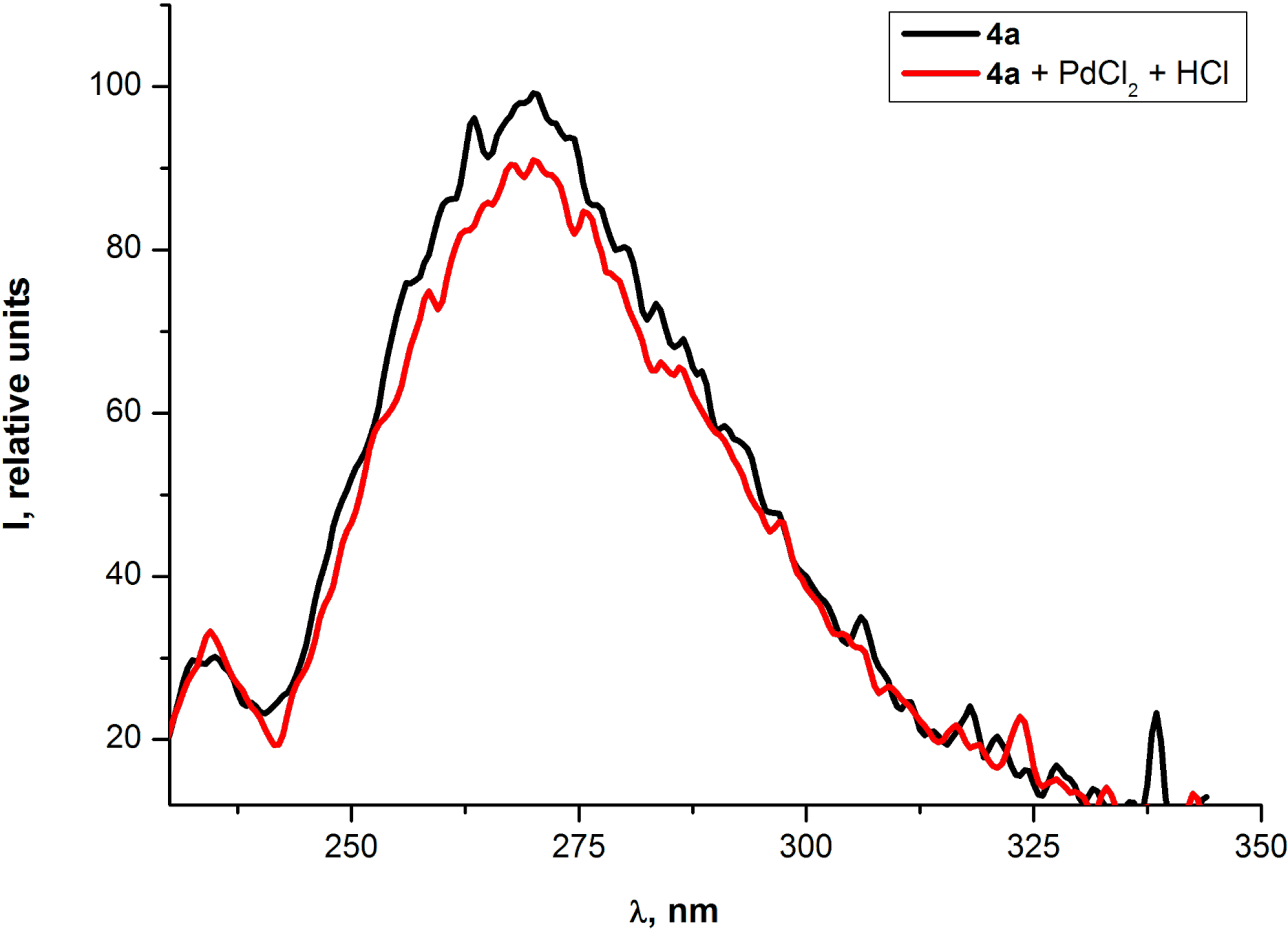
Emission spectra of cinnoline **4a** in THF before and after the addition of aqueous solution of PdCl_2_.

Previously palladium(II)-alkyne π-complexes have been proposed as intermediates in Pd(II)-catalyzed diamination of alkynes [63], enyne coupling [64], hydroarylation of alkynes [65], inramolecular carbocyclization [66] and other reactions. Some types of these complexes have been isolated [67]. Therefore, two structural features in the obtained oligomer chains can be responsible for the interaction with Pd ^2+^ cations: conjugated triple bonds (and/or the additional ethynyl moiety in the case of compound **10b**) and a cinnoline ring. In order to determine the role of the cinnoline fragment in this process, UV–vis and fluorescence emission spectra for the dibromocinnoline **4a**, the compound without any triple bonds, were measured. The observed "red" shift of absorption bands in the UV spectrum of the cinnoline **4a** in the presence of Pd^2+^ ions (Figure 16) suggested the interaction of Pd^2+^ with the cinnoline moiety. This allowed us to conclude that the cinnoline fragment is playing the role of a recognition site of Pd^2+^ cations. On the other hand the fluorescence quenching for compound **4a** (Figure 17) is much less effective than for PAE **10a** (Figure 10). This could be explained by a known collective effect in the conjugated chains [8–9].

## Conclusion

Cinnoline-containing poly(arylene ethynylene)s **10a,b** were synthesized for the first time via polycondensation based on one-pot removal of the TMS group and Cu^I^/Pd^0^-catalyzed Sonogashira coupling of bis(trimethylsilylethynyl)cinnolines and dioctylated diiodohydroquinone. The Pd^2+^-sensing ability of PAEs obtained was demonstrated by Pd^2+^-dependent fluorescence quenching of their THF solutions. This effect is likely due to the binding of Pd^2+^ cations to the cinnoline moiety of oligomers. The comparison of photochemical properties and Pd^2+^-sensing ability of PAEs obtained suggested that the incorporation of an additional triple bond into a cinnoline ring offers a more significant improvement to the fluorescence quantum yield and the Pd^2+^dependent quenching efficiency than increasing of the polymerization degree.

## Supporting Information

File 1 General information and methods, all synthetic procedures and analytical data, procedures for the investigation of cation sensing ability for PAEs **10a,b**.

File 2 Copies of ^1^H and ^13^C NMR spectra for all compounds, copies of DEPT and 2D NMR spectra for compounds **5a,b** and **7**, copies of IR spectra for compounds **4a,b**; **5,b**; **7** and **10a,b**, copies of GPC chromatograms for PAEs **10a,b**.
