# Supplementary material for: Synthesis and chemosensing properties of cinnoline-containing poly(arylene ethynylene)s
Source: Beilstein J Org Chem. 2015 Mar 20;11:373–84. doi: 10.3762/bjoc.11.43 (PMC4419556; doi:10.3762/bjoc.11.43)
Supplement: File 1 — General information and methods, all synthetic procedures and analytical data, procedures for the investigation of cation sensing ability for PAEs 10a,b. [file Beilstein_J_Org_Chem-11-373-s001.pdf]

Supporting Information File 1

for

Synthesis and chemosensing properties of cinnoline-containing poly(arylene ethynylene)s

Natalia A. Danilkina, Petr S. Vlasov, Semen M. Vodianik, Andrey A. Kruchinin, Yuri G. Vlasov, Irina A. Balova*

Address: Institute of Chemistry, Saint Petersburg State University, Universitetskiy pr. 26, Saint Petersburg, 198504, Russia. Fax:+7(812)4286733

Email: Irina A. Balova* - balova.irina@chem.spbu.ru

*Corresponding author

General information and methods, synthetic procedures and analytical data, procedures for the investigation of cations sensing ability for PAEs 10a,b.

Table of contents

General information and methods – S2

Synthetic procedures – S3

Investigation of cations sensing ability for PAEs **10a,b** – S12

References – S12

General information and methods

Solvents, reagents, and chemicals (**2a, 6**) used for reactions were purchased from commercial suppliers. Catalysts $Pd(PPh_3)_4$ was purchased in Sigma-Aldrich. Solvents were dried under standard conditions; chemicals were used without further purification. Trimethyl(nona-1,3-diynyl)silane (**2b**) [1] and 4-bromo-2-iodaniline [2], were synthesized by known procedures without any modification. 1,4-Diiodo-2,5-bis(octyloxy)benzene **9** and 1,4-diethynyl-2,5-bis(octyloxy)benzene were synthesized in accordance with general procedures described by M. J. Plater et al. [3]. All reactions were carried out under Ar in flame-dried glassware. Evaporation of solvents and concentration of reaction mixtures were performed in vacuo at 30−40 °C on a Heidolph rotary evaporator. Thin-layer chromatography (TLC) was carried out on silica gel plates (Silica gel 60, F254, Merck) with detection by UV or staining with a basic aqueous solution of $KMnO_4$. Normal-phase silica gel (Silica gel 60, 230−400 mesh, Merck) was used for preparative chromatography. Melting points (mp) determined by Stuart SMP30 equipment are uncorrected.

IR spectra were recorded for thin films on KBr on a Bruker Tensor-27 spectrometer. Absorption is reported as values in reciprocal centimeters (cm^{-1}). UV–vis spectra for solutions of all compounds in THF were recorded on a Shimadzu UV-2600 spectrophotometer at 20 °C. Fluorescence spectra for the same solutions were recorded on a Perkin-Elmer LS-55 fluorometer at 20 °C. ^{1}H and ^{13}C NMR 1D and 2D spectra were recorded at 300 and 75 MHz at a Bruker 300 MHz DPX spectrometer (for **3a,b**, **4a,b**) or at 400 and 100 MHz using a Bruker 400 MHz Avance spectrometer (for all other compounds) in $CDCl_3$. The ^{1}H NMR data are reported as chemical shifts (δ), multiplicity (s, singlet; d, doublet; t, triplet; q, quartet; m, multiplet; br, broad), coupling constants (*J*, given in Hz), and number of protons. The ^{13}C NMR data are reported as the chemical shift (δ). Chemical shifts are reported as δ values (ppm) referenced to residual solvent (δ = 7.26 ppm for ^{1}H; δ = 77.00 ppm for ^{13}C). High-resolution mass spectra (HRMS) were measured using electrospray ionization (ESI) in the mode of positive ion registration using Bruker Micro TOF equipment. Elemental analysis was performed on a Heraeus CHN-O-Rapid. Gel permeation chromatography (GPC) for polymer samples was carried out in THF at 30 °C on a Shimadzu LC-20AD chromatograph equipped with a TSKgel $G5000H_{HR}$ (Tosoh Bioscience) column and refractometric detector. The calibration was performed with nine polystyrene standards (Fluka) and approximated by third order polynomial function.

The quantum yield for THF solutions of PAEs **10a,b** was estimated in accordance with the procedure suggested by Horiba company (formerly Jobin Yvon) [4] and calculated as described therein. A solution of quinine sulfate in 0.1 M sulfuric acid was chosen as a quantum yield standard [5].

$$\Phi_X = \Phi_{ST}\left(\frac{Grad_X}{Grad_{ST}}\right)\left(\frac{\eta_X^2}{\eta_{ST}^2}\right)$$, where

Φx − quantum yield of the sample under investigation;

Φ_{ST} − quantum yield of the standard, Φ_{ST} = 0.54 [5];

$Grad_X$ – the slope of the dependence of the integrated fluorescence intensity on the solution optical density at the sample excitation wavelength for the sample under investigation;

$Grad_{ST}$ – the slope of the dependence of the integrated fluorescence intensity on the solution optical density at the standard excitation wavelength for the standard.

n_X – the refractive index of THF at 20 °C, n_X = 1.407.

n_{ST} – 1.334 refractive index of 0.1 M sulfuric acid at 20 °C, n_{ST} = 1.334

Synthetic procedures

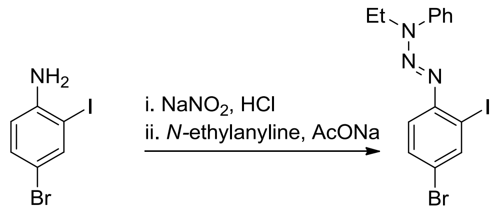

1

1-(4-Bromo-2-iodophenyl)-3-ethyl-3-phenyltriaz-1-ene (1). A mixture of 4-bromo-2-iodoaniline (50.0 mmol, 14.9 g) [2] and concentrated HCl (14.3 mL) was heated in a beaker at 100 °C over 1−2 minutes affording a fine dispersed residue of 4-bromo-2-iodaniline hydrochloride. The suspension formed was cooled to −5 °C and diazotized with a solution of $NaNO_2$ (55.0 mmol, 3.79 g) in water (11.0 mL). Then to the solution obtained a cooled to 0 °C solution of *N*-ethylaniline (50.0 mmole, 6.10 g) in the mixture of H_2O (3.60 mL), EtOH (10.0 mL) and concentrated HCl (3.60 mL) was added in one portion. The resulting mixture was immediately poured into a cooled (10 °C) solution of NaOAc (40.0 g) in H_2O (150 mL) with 36.0 g of ice inside. The resulting mixture was stirred for 1 hour at room temperature and then

put into the fridge overnight. The solid formed was filtered off, washed with water, air dried and recrystallized from MeCN (long heating in MeCN led to a yield reduction) and twice from EtOH to give 15.9 g (74%) of **1** as yellow-orange crystals. Mp 81−82 °C. ^{1}H NMR (400 MHz, $CDCl_3$, *δ*): 1.41 (t, *J* =7.1, 3H, CH_3), 4.38 (q, *J* = 7.1, 2H, CH_2), 7.19 (t, *J* = 7.2, 1H, H_{Ar}), 7.38−7.49 (m, 6H, H_{Ar}), 8.05 (d, *J* = 2.0, Hz, H_{Ar}). ^{13}C NMR (75 MHz, $CDCl_3$, *δ*): 10.9, 41.2, 98.0, 117.0, 118.7, 119.9, 124.1, 129.3, 131.7, 141.0, 143.7, 148.9.

Sonogashira coupling

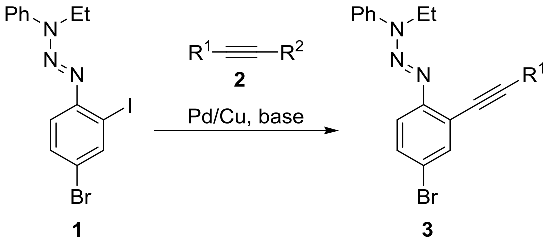

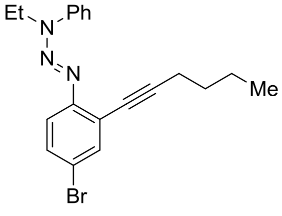

3a

1-[4-Bromo-2-(hex-1-yn-1-yl)phenyl]-3-ethyl-3-phenyltriaz-1-ene (3a). To a degassed solution of triazene **1** (2.00 mmol, 860 mg) in the mixture of Et_3N (8.00 mL) and THF (4.00 mL) were added $Pd(PPh_3)_4$ (0.100 mmol, 116 mg), PPh_3 (0.200 mmol, 52.4 mg) and hex-1-yne (**2a**, 3.00 mmol, 246 mg, 0.346 mL). The resulting mixture was stirred under Ar over 5 min, then CuI (0.3 mmol 57.0 mg) was added and the reaction mixture was stirred at 35 °C for 25 minutes (TLC monitoring). The resulting mixture was poured into a saturated aqueous solution of NH_4Cl, extracted with ethyl acetate, washed with a saturated solution of NH_4Cl and brine, dried over anhydrous Na_2SO_4, and concentrated under reduced pressure to yield the crude product, which was purified by column chromatography on silica gel using petroleum ether/dichloromethane (10:1→6:1) as the eluent to give 587 mg (76%) of **1a** as an orange oil. ^{1}H NMR (300.13 MHz, $CDCl_3$, *δ*): 0.94 (t, *J* =7.1, 3H, CH_3), 1.35 (t, *J* =7.0, 3H, CH_3), 1.46−1.55 (m, 2H, CH_2), 1.57−1.64 (m, 2H, CH_2), 2.47 (t, *J* =7.0, 2H, CH_2), 4.37 (q, *J* = 7.1, 2H, CH_2), 7.14 (t, *J* = 7.3, 1H, H_{Ar}), 7.37−7.41 (m, 4H, H_{Ar}), 7.46−7.48 (m, 2H, H_{Ar}), 7.60 (d, *J* = 1.5, 1H, H_{Ar}). All other analytical data of the compound **3a** obtained are in a good accordance with the data reported previously [6].

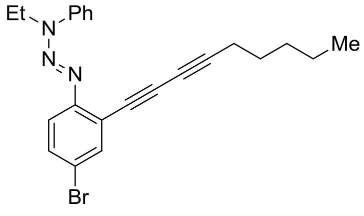

3b

1-[4-Bromo-2-(nona-1,3-diyn-1-yl)phenyl]-3-phenyl-3-ethyltriaz-1-ene (3b). To the degassed solution of triazene **1** (4.30 mmol, 1.85 g) in anhydrous DMF (45.0 mL) were added $Pd(PPh_3)_4$ (0.220 mmol, 254 mg), KF (21.5 mmol, 1.25 g), CuI (0.645 mmol, 123 mg). The reaction mixture was evacuated and flushed with Ar several times and stirred under Ar for 5 min; then, MeOH (43.0 mmol, 1.38 g, 1.74 mL) followed by trimethyl(nona-1,3-diynyl)silane **2b** (8.6 mmol, 1.65 g) [1] was added. The reaction mixture was stirred at room temperature for 2 hours (TLC monitoring), poured into a saturated aqueous solution of NH_4Cl, extracted with DCM (3×30 mL), washed with a saturated solution of NH_4Cl and brine, dried over anhydrous Na_2SO_4, and concentrated under reduced pressure to yield the crude product, which was purified by column chromatography on silica gel using petroleum ether/ethyl acetate (3:1) as the eluent to give 1.63 g (90%) of **1b** as an orange oil. ^1H NMR (300 MHz, $CDCl_3$, δ): 0.93 (t, J =7.0, 3H, CH_3), 1.29–1.47 (m, 7H, 2×CH_2, CH_3), 1.55–1.64 (m, 2H, CH_2), 2.38 (t, J =7.0, 2H, CH_2), 4.37 (q, J = 7.0, 2H, CH_2), 7.16 (t, J = 7.2, 1H, H_{Ar}), 7.38–7.45 (m, 4H, H_{Ar}), 7.47–7.50 (m, 2H, H_{Ar}), 7.66 (s, 1H, H_{Ar}). ^{13}C NMR (75 MHz, $CDCl_3$, δ): 10.8, 13.9, 19.6, 22.1, 27.9, 31.0, 40.9, 65.3, 71.5, 79.9, 86.1, 117.1, 118.8, 119.3, 119.8, 124.0, 129.2, 132.4, 136.1, 143.9, 152.0. ESI-HRMS (m/z): calcd for $C_{23}H_{25}BrN_3$, 422.1232 $[M+H]^+$; found, 422.1217. calcd for $C_{23}H_{24}BrN_3Na$, 444.1051 $[M+Na]^+$; found, 444.1039.

General procedure for the synthesis of 4,6-bromocinnolines 4.

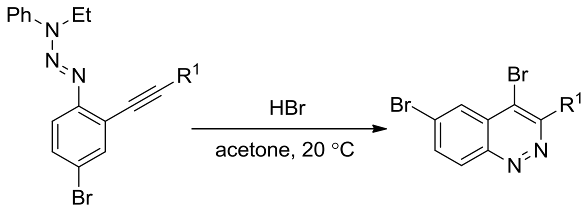

3 4

Concentrated hydrobromic acid (50.0 mmol, 8.40 g, 5.70 mL,) was promptly added to the solution of corresponding triazene **3** (2.5 mmol) in acetone (25.0 mL) at such a way so as to maintain the temperature of the reaction mixture at 20 °C (water cooling bath). The reaction mixture was stirred at 20 °C for 5 minutes (in the case of **3a**) and for 15 minutes (in the case of **3b**), then, it was poured into a solution of Et_3N (50.0 mmol, 5.06 g, 7.00 mL) in H_2O (100 mL) and extracted with DCM (3×20 mL). The combined organic layers were washed with a saturated aqueous solution of NH_4Cl, H_2O, dried over anhydrous

Na_2SO_4 and concentrated under reduced pressure to yield the crude product, which was purified by column chromatography on silica gel using petroleum ether/ethyl acetate (20:1→10:1) as the eluent.

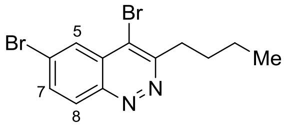

4a

4,6-dibromo-3-butylcinnoline (4a) was synthesized in accordance with the General Procedure from triazene **3a** (2.5 mmol, 961 mg). Yield 410 mg (87%). Mp 57–59 °C (lit. [7] 59–61 °C). ^{1}H NMR (300 MHz, $CDCl_3$, δ): 1.00 (t, J =7.3, 3H, CH_3), 1.44–1.56 (m, 2H, CH_2), 1.83–1.91 (m, 2H, CH_2), 3.41 (t, J =7.8, 2H, CH_2), 7.87 (dd, ^3J = 9.0, ^4J = 1.7, 1H, H^7), 8.31 (d, ^4J = 1.7, 1H, H^5), 8.36 (d, ^3J = 9.0, 1H, H^8). ^{13}C NMR (75 MHz, $CDCl_3$, δ): 13.9, 22.6, 31.1, 36.1, 125.3, 127.6, 127.8, 128.1, 131.7, 134.0, 148.1, 158.0. IR (KBr) ν_{max} (cm^{-1}): 3078, 3023, 2955, 2926, 2868, 2858, 1600, 1547, 1455, 1414, 1380, 1328, 1300, 1278, 1226, 1197, 1117, 1105, 1068, 992, 947, 921, 883, 828, 787, 767, 746, 653, 608. ESI-HRMS (m/z): calcd for $C_{12}H_{12}Br_2N_2$, 342.9445 [M+H]$^+$; found, 342.9408. calcd for $C_{12}H_{11}Br_2N_2Na$, 364.9265 [M+Na]$^+$; found, 364.9232. calcd for $C_{12}H_{12}Br_2N_2K$, 380.9004 [M+K]$^+$; found, 380.8968.

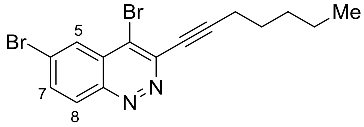

4b

4,6-dibromo-3-(hept-1-yn-1-yl)cinnoline (**4b**) was synthesized in accordance with the General Procedure from triazene **3b** (2.5 mmol, 1.06 g). Yield 554 mg (58%). Mp 68–70 °C. ^{1}H NMR (300 MHz, $CDCl_3$, δ): 0.94 (t, J =7.2, 3H, CH_3), 1.34–1.41 (m, 2H, CH_2), 1.46–1.59 (m, 2H, CH_2), 1.70–1.80 (m, 2H, CH_2), 2.63 (t, J =7.0, 2H, CH_2), 7.91 (dd, ^3J = 9.0, ^4J = 1.7, 1H, H^7), 8.31 (d, ^4J = 1.7, 1H, H^5), 8.39 (d, ^3J = 9.0, 1H, H^8). ^{13}C NMR (75 MHz, $CDCl_3$, δ): 14.0, 19.8, 22.2, 27.8, 31.1, 77.9, 101.0, 127.1, 127.4, 128.2, 128.3, 131.9, 134.8, 143.6, 146.9. IR (KBr) ν_{max} (cm^{-1}): 3085, 3021, 2954, 2927, 2857, 2234, 1597, 1542, 1452, 1412, 1377, 1330, 1300, 1298, 1238, 1223, 1148, 1114, 1061, 1032, 968, 931, 866, 830, 774, 729, 688, 653, 608. ESI-HRMS (m/z): calcd for $C_{15}H_{15}Br_2N_2$, 380.9602 [M+H]$^+$; found, 380.9594. calcd for $C_{15}H_{14}Br_2N_2Na$, 402.9421 [M+Na]$^+$; found, 402.9407.

Optimization of the double Sonogashira coupling (see Table 2).

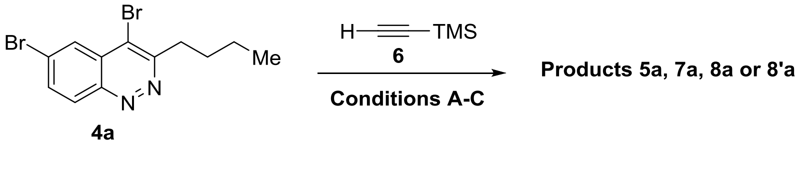

Conditions A

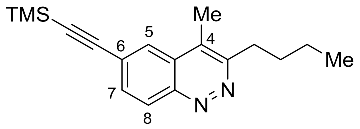

7a

3-Butyl-4-methyl-6-[(trimethylsilyl)ethynyl]cinnoline (**7a).** To a degassed solution of dibromocinnoline **4a** (0.291 mmol, 100 mg) in anhydrous DMF (3.00 mL) were added PPh_3 (0.0145 mmol, 3.81 mg), diisopropanolamine (DIPA) (1.16 mmol, 155 mg), $Pd(PPh_3)_4$ (0.0145mmol, 16.7 mg), CuI (0.0436 mmol, 8.20 mg). The resulting solution was evacuated and flushed with Ar several times; then, trimethylsilylacetylene (1.46 mmol, 144 mg, 0.208 mL) was added, and the reaction mixture was allowed to stir at 80 °C for 24 hours (TLC monitoring). The resulting mixture was poured into a saturated aqueous solution of NH_4Cl, extracted with ethyl acetate (3×20 mL), washed with saturated solution of NH_4Cl and brine, dried over anhydrous Na_2SO_4, and concentrated under reduced pressure to yield the crude product, which was purified by column chromatography on silica gel using hexane/ ethyl acetate (15:1→10:1→5:1) as the eluent to give 42.8 mg (50%) of **7a** as an orange oil. ^{1}H NMR (400 MHz, $CDCl_3$, δ): 0.32 (s, 9H, TMS), 1.00 (t, J =7.3, 3H, CH_3), 1.45−1.54 (m, 2H, CH_2), 1.78−1.86 (m, 2H, CH_2), 2.64 (s, 3H, CH_3), 3.26 (t, J =7.8, 2H, CH_2), 7.73 (d, ^3J = 8.7, 1H, H^7), 8.10 (d, ^4J = 1.3, 1H, H^5), 8.37 (d, ^3J = 8.7, 1H, H^8). ^{13}C NMR (100 MHz, $CDCl_3$, δ): δ -0.22 (TMS), 12.8 (Me), 13.9 (Me), 22.7 (CH_2), 31.9 (CH_2), 33.7 (CH_2), 98.2(C_{sp}), 104.2 (C_{sp}), 125.3(C_{Ar}), 126.0 (C_{Ar}), 126.7 (CH_{Ar}), 127.8 (C_{Ar}), 130.2 (CH_{Ar}), 131.6 (CH_{Ar}), 147.4 (C_{Ar}), 157.0 (C_{Ar}). IR (KBr) ν_{max} (cm^{-1}): 2958, 2931, 2872, 2160, 1614, 1559, 1473, 1431, 1294, 1250, 1195, 1158, 1117, 946, 858, 844, 760, 700, 635. ESI-HRMS (m/z): calcd for $C_{18}H_{25}N_2Si$, 297.1787 [M+H]$^+$; found, 297.1807. calcd for $C_{18}H_{24}N_2Na$, 319.1606 [M+Na]$^+$; found, 319.1612. calcd for $C_{18}H_{24}N_2K$, 335.1346 [M+K]$^+$; found, 335.1351.

Conditions B

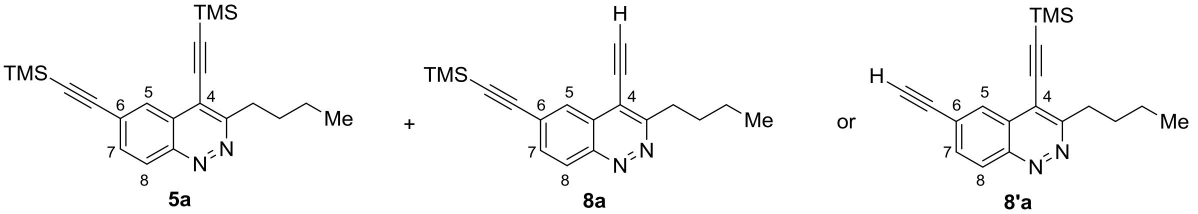

5a **8a** **8'a**

The mixture of 3-butyl-4,6-bis[(trimethylsilyl)ethynyl]cinnoline (**5a**) **and monodesilylated cinnoline 8a (or 8’a).** To the degassed solution of dibromocinnoline **4a** (0.150 mmol, 50.0 mg) in anhydrous Et_3N (2.0 mL) were added $Pd(PPh_3)_4$ (0.0075 mmol, 8.67 mg), PPh_3 (0.0075 mmol, 2.00 mg), CuI (0.0225

mmol, 4.30 mg) and trimethylsilylacetylene (0.45 mmol, 44.2 mg, 63.5 µL). The mixture was stirred under an argon atmosphere at 50 °C for 20 hours. However even after 20 hours only the starting material was detected by TLC monitoring. The resulting mixture was cooled, poured into a saturated aqueous solution of NH_4Cl, extracted with DCM (3×10 mL), washed with a saturated solution of NH_4Cl and H_2O, dried over anhydrous Na_2SO_4, and concentrated under reduced pressure to yield the crude product, which was purified by column chromatography on silica gel using petroleum ether/ethyl acetate (7:1) as the eluent to give 30.0 mg of a mixture of **5a** (34%) **/ 8a (or 8'a)** (23%)(1.5:1). For the ^{1}H NMR spectrum of this mixture see Supporting Information File 2. The elucidation of the position of a desilylated triple pond was not performed.

Conditions C

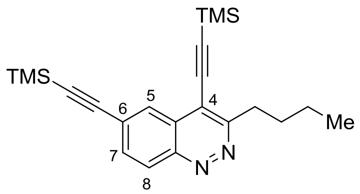

5a

3-Butyl-4,6-bis[(trimethylsilyl)ethynyl]cinnoline (**5a**)**.** To the degassed solution of dibromocinnoline **4a** (0.150 mmol, 50.0 mg) in anhydrous Et_3N (2.0 mL) were added $Pd(PPh_3)_4$ (0.0075 mmol, 8.67 mg), PPh_3 (0.0075 mmol, 2.00 mg), CuI (0.0225 mmol, 4.30 mg) and trimethylsilylacetylene (0.45 mmol, 44.2 mg, 63.5 µL). The mixture was stirred under an argon atmosphere at 40 °C for 3.5 hours. The resulting mixture was cooled, poured into a saturated aqueous solution of NH_4Cl, extracted with DCM (3×10 mL), washed with saturated solution of NH_4Cl and H_2O, dried over anhydrous Na_2SO_4, and concentrated under reduced pressure and the crude reaction product was analyzed by ^{1}H NMR spectroscopy. ^{1}H NMR (300 MHz, $CDCl_3$): δ 0.31 (s, 9H, TMS), 0.37 (s, 9H, TMS), 0.99 (t, J =7.3, 3H, CH_3), 1.42−1.54 (m, 2H, CH_2), 1.83−1.94 (m, 2H, CH_2), 3.36 (t, J =7.8, 2H, CH_2), 7.75 (dd, ^3J = 8.8, ^4J = 1.3, 1H, H^7) 8.21 (s, 1H, H^5), 8.38 (d, ^3J = 8.8, 1H, H^8).

Synthesis of 4,6-bis[(trimethylsilyl)ethynyl]cinnolines (5a,b) using optimized conditions C.

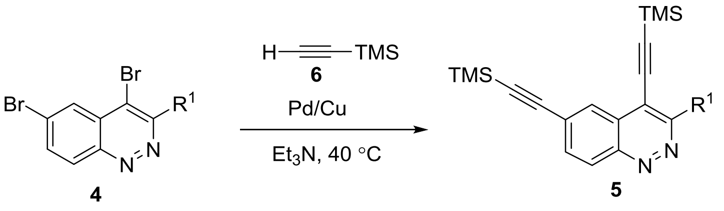

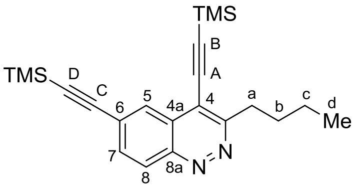

5a

3-Butyl-4,6-bis[(trimethylsilyl)ethynyl]cinnoline (**5a**)**.** The compound **5a** was synthesized under conditions C from dibromocinnoline **4a** (0.6 mmol, 206 mg) and trimethylsilylacetylene (1.8 mmol, 177 mg, 0.254 mL) in Et_3N (8.0 mL) using PPh_3 (0.03 mmol, 7.86 mg), $Pd(PPh_3)_4$ (0.03 mmol, 35.7 mg) and CuI (0.09 mmol, 17.1 mg) as a catalytic system. It is essential not to heat the reaction mixture over 30 °C during the work up and the purification. Purification of the crude product by column chromatography on silica gel using petroleum ether/ethyl acetate (7:1) as the eluent gave 200 mg (88%) of **5a** as a yellow oil. ^{1}H NMR (400 MHz, $CDCl_3$, δ): 0.31 (s, 9H, TMS), 0.37 (s, 9H, TMS), 0.98 (t, J =7.3, 3H, CH_3), 1.43−1.52 (m, 2H, CH_2), 1.84−1.92 (m, 2H, CH_2), 3.36 (t, J =7.8, 2H, CH_2), 7.75 (dd, ^3J = 8.8, ^4J = 1.3, 1H, H^7) 8.21 (d, ^4J = 1.3, 1H, H^5), 8.38 (d, ^3J = 8.8, 1H, H^8). ^{13}C NMR (100 MHz, $CDCl_3$, δ): δ -0.3 (TMS),-0.2 (TMS), 13.9 (CH_3^d), 22.7 (CH_2^c), 31.6 (CH_2^b), 34.9 (CH_2^a), 97.3 (C^A), 99.4 ($C^{B\ or\ D}$), 104.0 (C^C), 112.0 ($C^{B\ or\ D}$), 115.8 (C^4), 125.7 ($C^{4a\ or\ 6}$), 126.4 ($C^{4a\ or\ 6}$), 128.5 (C^5), 130.0 (C^8), 132.6 (C^7), 147.3 (C^{8a}), 159.5 (C^3). IR (KBr) ν_{max} (cm^{-1}): 2960, 2930, 2661, 2155, 1612, 1550, 1523, 1468, 1424, 1299, 1250, 1194, 1094, 912, 844, 760, 699, 647. ESI-HRMS (m/z): calcd for $C_{22}H_{31}N_2Si_2$, 379.2026 $[M+H]^+$; found, 379.2025. calcd for $C_{22}H_{30}N_2NaSi_2$, 401.1845 $[M+Na]^+$; found, 401.1831.

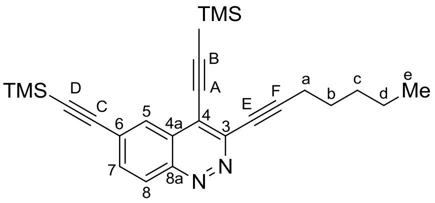

5b

3-(Hept-1-yn-1-yl)-4,6-bis[(trimethylsilyl)ethynyl]cinnoline (**5b**). The compound **5b** was synthesized under conditions C from dibromocinnoline **4b** (0.6 mmol, 229 mg) and trimethylsilylacetylene (1.8 mmol, 177 mg, 0.254 mL) in Et_3N (8.0 mL) using PPh_3 (0.03 mmol, 7.86 mg), $Pd(PPh_3)_4$ (0.03 mmol, 35.7 mg) and CuI (0.09 mmol, 17.1 mg) as a catalytic system. It is essential not to heat the reaction mixture over 30 °C during the work up and the purification. Purification of the crude product by column chromatography on silica gel using petroleum ether/ethyl acetate (7:1) as the eluent gave 228 mg (91%) of **5b** as a yellow oil. ^{1}H NMR (400 MHz, $CDCl_3$, δ): 0.38 (s, 9H, TMS), 0.40 (s, 9H, TMS), 0.93 (t, J =7.2, 3H, CH_3), 1.36−1.42 (m, 2H, CH_2), 1.49−1.53 (m, 2H, CH_2), 1.72−1.76 (m, 2H, CH_2), 2.60 (t, J =7.1, 2H, CH_2), 7.79 (dd, ^3J = 8.7, ^4J = 1.6, 1H, H^7) 8.19 (d, ^4J = 1.6, 1H, H^5), 8.41 (d, ^3J = 8.7, 1H, H^8). ^{13}C NMR (100 MHz, $CDCl_3$, δ): -0.28 (TMS), -0.26 (TMS), 13.9 (CH_3^e), 19.9 (CH_2^a), 22.2 (CH_2^d), 28.1 (CH_2^b), 31.2 (CH_2^c), 77.8 (C^E), 97.2 (C^A), 99.3 (C^F), 100.1 ($C^{B\ or\ D}$), 103.7 (C^C), 112.8 ($C^{B\ or\ D}$), 119.5 (C^4), 124.9 ($C^{4a\ or\ 6}$), 127.0 ($C^{4a\ or\ 6}$), 128.4 (C^5), 130.2 (C^8), 133.5 (C^7), 143.2 (C^3), 146.4 (C^{8a}). IR (KBr) ν_{max}

(cm^{-1}): 2958, 2935, 2900, 2870, 2227, 2158, 1608, 1545, 1511, 1466, 1416, 1347, 1305, 1246, 1196, 1123, 1072, 1038, 909, 894, 847, 759, 736, 702, 684, 634, 615. ESI-HRMS (m/z): calcd for $C_{25}H_{33}N_2Si_2$, 417.2182 $[M+H]^+$; found, 417.2172. calcd for $C_{25}H_{32}N_2NaSi_2$, 439.2002 $[M+Na]^+$; found, 439.1983. calcd for $C_{25}H_{32}N_2KSi_2$, 455.1741 $[M+K]^+$; found, 455.1723.

General procedure for the synthesis of cinnoline-containing PAEs 10a,b.

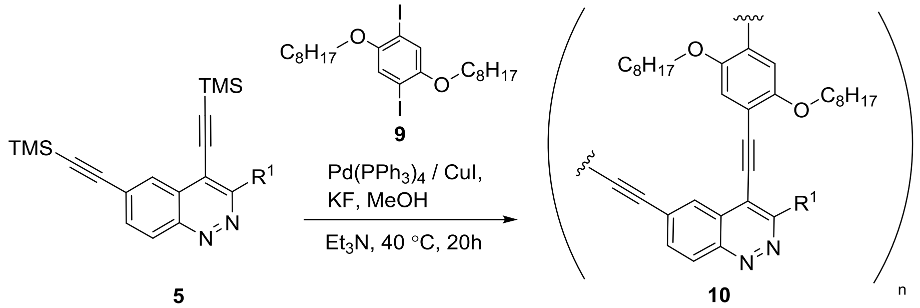

To the degassed solution of 1,4-diiodo-2,5-bis(octyloxy)benzene **9** (0.12 mmol, 70.4 mg) [3] in Et_3N (1.0 mL) in a vial were added $Pd(PPh_3)_4$ (0.0066 mmol, 7.62 mg,), CuI (0.0132 mmol, 2.51 mg), KF (1.32 mmol, 76.7 mg), and 3,6-bis(trimethylsilylethynyl)cinnoline **5** (0.132 mmol). The vial was sealed, degassed and flushed with argon. Then, to the reaction mixture was added MeOH (2.64 mmol, 84.5 mg, 0.11 mL,), and the resulting mixture was stirred at 40 °C for 20 hours (TLC monitoring). The reaction mixture was cooled, poured into 5% aqueous solution of NH_3 (40 mL), and extracted with $CHCl_3$ (3×10 mL). The combined organic layers were washed with 5% aqueous solution of NH_3 and H_2O, dried over anhydrous Na_2SO_4 and concentrated under reduced pressure to yield the crude oligomer, which was dissolved in a small amount of $CHCl_3$ and precipitated by adding of MeOH. The orange powder formed was filtered off, washed with MeOH and dried under vacuum to give PAEs **10a,b** in quantitative yields.

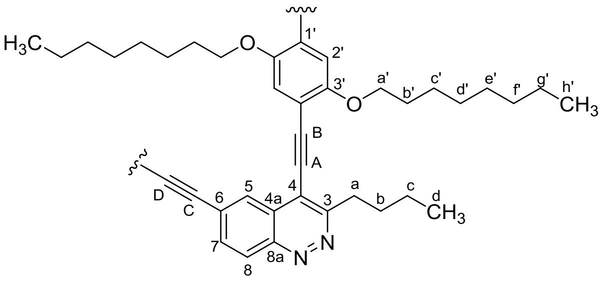

10a

Poly[(3-butylcinnoline-4,6-diyl)-ethyne-1,2-diyl-(2,5-bis(octyloxy)-1,4-phenylene)-ethyne-1,2-diyl] (PAE 10a). The oligomer **10a** was synthesized as described for the general procedure using 3,6-bis(trimethylsilylethynyl)cinnoline **5a** (0.132 mmol, 50.0 mg). Yield 67.0 mg (99%, calculated for the repeating unit). ^{1}H NMR (400 MHz, $CDCl_3$, δ): 0.75 (s, 6H, 2 × $CH_3^{h'}$), 1.00−1.58 (m, 25H, CH_2^{c}, 2 × $CH_2^{c'-g'}$, CH_3^{d}), 1.81−2.05 (m, 6H, 2 × $CH_2^{b'}$, CH_2^{b}), 3.50 (s, 2H, CH_2^{a}), 4.04−4.17 (m, 4H, 2 × $OCH_2^{a'}$), 7.09−7.16 (m, 2H, $H^{2'}$), 7.87 (s, 1H, H^7), 8.44−8.55 (m, 2H,

$H^{5,8}$). ^{13}C NMR (100 MHz, $CDCl_3$, δ): 14.0 (CH_3^{d}, 2 × $CH_3^{h'}$) 22.55 and 22.65 (CH_2^{c}, 2 × $CH_2^{g'}$), 26.08−26.15 (2 × $CH_2^{c'}$), 29.26−29.38 (2 × $CH_2^{b',d',e'}$), 31.72 and 31.76 (CH_2^{b}, 2 × $CH_2^{f'}$), 34.9 (CH_2^{a}), 69.5−70.0 (2 × $OCH_2^{a'}$), 88.3−88.6 ($C^{B\ or\ D}$), 90.2 ($C^{B\ or\ D}$), 94.8−95.1 (C^{A}), 101.1 (C^{C}), 114.1 (C^{4}), 116.1 (2 × $C^{1'}$), 116.8−117.0 (2 × $C^{2'}$), 125.8 (C^{6}), 126.6 (C^{4a}), 127.8 (C^{5}), 130.2 (C^{8}), 132.6 (C^{7}), 147.5 (C^{8a}), 154.02−154.33 (2 × $C^{'3}$), 158.8 (C^{3}). IR (KBr) ν_{max} (cm^{-1}): 3058, 2953, 2925, 2855, 2202, 1610, 1548, 1500, 1466, 1437, 1388, 1277, 1216, 1078, 1023, 886, 856, 834, 721, 692, 638. SEC, M_w 13300, M_n 5380. Anal. Calcd for $C_{38}H_{48}N_2O_2$ (repeating unit): C, 80.81; H, 8.57, N 4.96; for $C_{326}H_{420}I_2N_{16}O_{18}$ (oligomer with both iodohydroquinone end groups n = 8): C, 76.70; H, 8.29, N 4.39. Found: C, 76.16; H, 7.97, N 4.57.

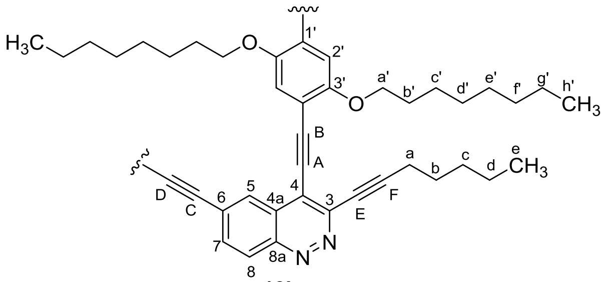

10b

Poly[(3-(hept-1-ynyl)cinnoline-4,6-diyl)-ethyne-1,2-diyl-(2,5-bis(octyloxy)-1,4-phenylene)-ethyne-1,2-diyl] (PAE 10b). The oligomer **10b** was synthesized as described in the general procedure using 3,6-bis(trimethylsilylethynyl)cinnoline **5b** (0.132 mmol, 55.0 mg). Yield 71.0 mg (98%, calculated for the repeating unit). ^{1}H NMR (400 MHz, $CDCl_3$, δ): 0.70−0.90 (m, 9H, CH_3^{e}, 2 × $CH_3^{h'}$), 1.05−1.57 (m, 24H, $CH_2^{c,d}$, 2 × $CH_2^{c'-g'}$), 1.75−2.01 (m, 6H, CH_2^{b}, 2 × $CH_2^{b'}$), 2.66 (s, 2H, CH_2^{a}), 4.04−4.13 (m, 4H, 2 × $OCH_2^{a'}$), 7.07−7.16 (m, 2H, $H^{2'}$), 7.88−7.90(m, 1H, H^{7}), 8.49−8.51 (m, 2H, $H^{5,8}$). ^{13}C NMR (100 MHz, $CDCl_3$, δ): 13.92 and 14.01 (CH_3^{e}, 2 × $CH_3^{h'}$) 19.9 (CH_2^{a}), 22.19 (CH_2^{d}), 22.54−22.60 (2 × $CH_2^{g'}$), 26.1 (2 × $CH_2^{c'}$), 28.2 (CH_2^{b}), 29.3 (2 × $CH_2^{b',d',e'}$), 31.2 (CH_2^{c}), 31.71− 1.77 (2 × $CH_2^{f'}$), 69.67−69.71 (2 × $OCH_2^{a'}$), 78.2 (C^{E}), 88.0 ($C^{B\ or\ D}$), 90.7 ($C^{B\ or\ D}$), 94.6 (C^{A}), 99.2, (C^{F}), 101.5 (C^{C}), 114.1−114.3 (2 × $C^{1'}$), 116.4−116.8 (2 × $C^{2'}$), 119.9 (C^{4}), 125.0 ($C^{4a\ or\ 6}$), 127.4 ($C^{4a\ or\ 6}$), 127.7 (C^{5}), 130.3 (C^{8}), 133.5 (C^{7}), 142.5 (C^{3}), 146.5 (C^{8a}), 154.14−154.46 (2 × $C^{3'}$). IR (KBr) ν_{max} (cm^{-1}): 3058, 2925, 2854, 2220, 2202, 1608, 1545, 1499, 1466, 1435, 1387, 1328, 1301, 1277, 1216, 1145, 1113, 1024, 890, 856, 834, 790, 722, 693, 638. SEC, M_w 9150, M_n 3670. Anal. Calcd for $C_{41}H_{50}N_2O_2$ (repeating unit): C, 81.69; H, 8.36, N 4.65; for $C_{268}H_{336}I_2N_{12}O_{14}$ (oligomer with both iodohydroquinone end groups, n = 6): C, 76.58; H, 8.06, N 4.00 Found: C, 77.03; H, 7.76, N 4.24.

Investigation of cations sensing ability for the solutions of PAEs 10a,b in THF.

Stock aqueous solutions of salts $PdCl_2$, $La(NO_3)_3$, $AgNO_3$ (5 mM) were prepared once and used when necessary. For the preparation of aqueous solution of $PdCl_2$, 10-fold excess of HCl was used. Stock solutions (1.0 mM) of PAE **10a,b** in THF were prepared from polymers samples and THF and stored not longer than 5 days. The M_w of repeating units of PAEs **10a,b** was used for all calculations. Working solutions of PAEs **10a,b** (5.0 μM, 2.8 mL) for the screening of their metal ions sensing ability were prepared by dilution of corresponding stock solutions with THF just before use. For the each experiment, first, the initial fluorescence emission intensity was measured, and then, an aliquot of the corresponding stock salt solution (2.8 μL, equimolar amount of the salt regarding to the repeating unit of the oligomer) was added directly to the cuvette. The solution obtained was stirred using magnetic stirring bar directly in the cuvette for 2 minutes, then the fluorescence intensity of the resulting solution was measured. All measurements were carried out using the excitation wavelength of 425 nm.

References

[1] Kyi, S.; Wongkattiya, N.; Warden, A. C.; O'Shea, M. S.; Deighton, M.; Macreadie, I.; Graichen, F. H. M. *Bioorg. Med. Chem. Lett*., **2010**, *20* (Suppl. S36), 4555–4557. doi: 10.1016/j.bmcl.2010.06.020

[2] Yin, Y.; Ma, W.; Chai, Z.; Zhao, G. *J. Org. Chem.* **2007,** *72* (Suppl. S6), 5731−5736. doi: 10.1021/jo070681h

[3] Plater, M. J.; Sinclair, J. P.; Aiken, S.; Gelbrich, T.; Hursthouse, M. B. *Tetrahedron* **2004**, *60*, 6385−6394. doi: 10.1016/j.tet.2004.03.059

[4] http://www.horiba.com/fileadmin/uploads/Scientific/Documents/Fluorescence/quantumyieldstrad.pdf

[5] Brouwer, A. M. *Pure Appl. Chem.* **2011**, 83, 2213–2228. doi: 10.1351/PAC-REP-10-09-31

[6] Danilkina, N. A.; Gorbunova, E. G.; Sorokoumov, V. N.; Balova, I. A. *Russ. J. Org. Chem.* **2012**, *48*, 1424–1434. doi: 10.1134/S1070428012110048.
