# Supplementary material for: Synthesis and chemosensing properties of cinnoline-containing poly(arylene ethynylene)s
Source: Beilstein J Org Chem. 2015 Mar 20;11:373–84. doi: 10.3762/bjoc.11.43 (PMC4419556; doi:10.3762/bjoc.11.43)
Supplement: File 2 — Copies of 1H and 13C NMR spectra for all compounds, copies of DEPT and 2D NMR spectra for compounds 5a,b and 7, copies of IR spectra for compounds 4a,b; 5,b; 7 and 10a,b, copies of GPC chromatograms for PAEs 10a,b. [file Beilstein_J_Org_Chem-11-373-s002.pdf]

Supporting Information File 2

for

Synthesis and chemosensing properties of cinnoline-containing poly(arylene ethynylene)s

Natalia A. Danilkina, Petr S. Vlasov, Semen M. Vodianik, Andrey A. Kruchinin, Yuri G. Vlasov, Irina A. Balova*

Address: Institute of Chemistry, Saint Petersburg State University, Universitetskiy pr. 26, Saint Petersburg, 198504, Russia. Fax:+7(812)4286733

Email: Irina A. Balova* - balova.irina@chem.spbu.ru

*Corresponding author

Copies of ^{1}H and ^{13}C NMR spectra for all compounds, copies of DEPT and 2D NMR spectra for compounds 5a,b and 7, copies of IR spectra for compounds 4a,b; 5,b; 7 and 10a,b, copies of GPC chromatograms for PAEs 10a,b.

Table of contents

Copies of NMR and IR spectra – S2

Copies of GPC chromatograms – S45

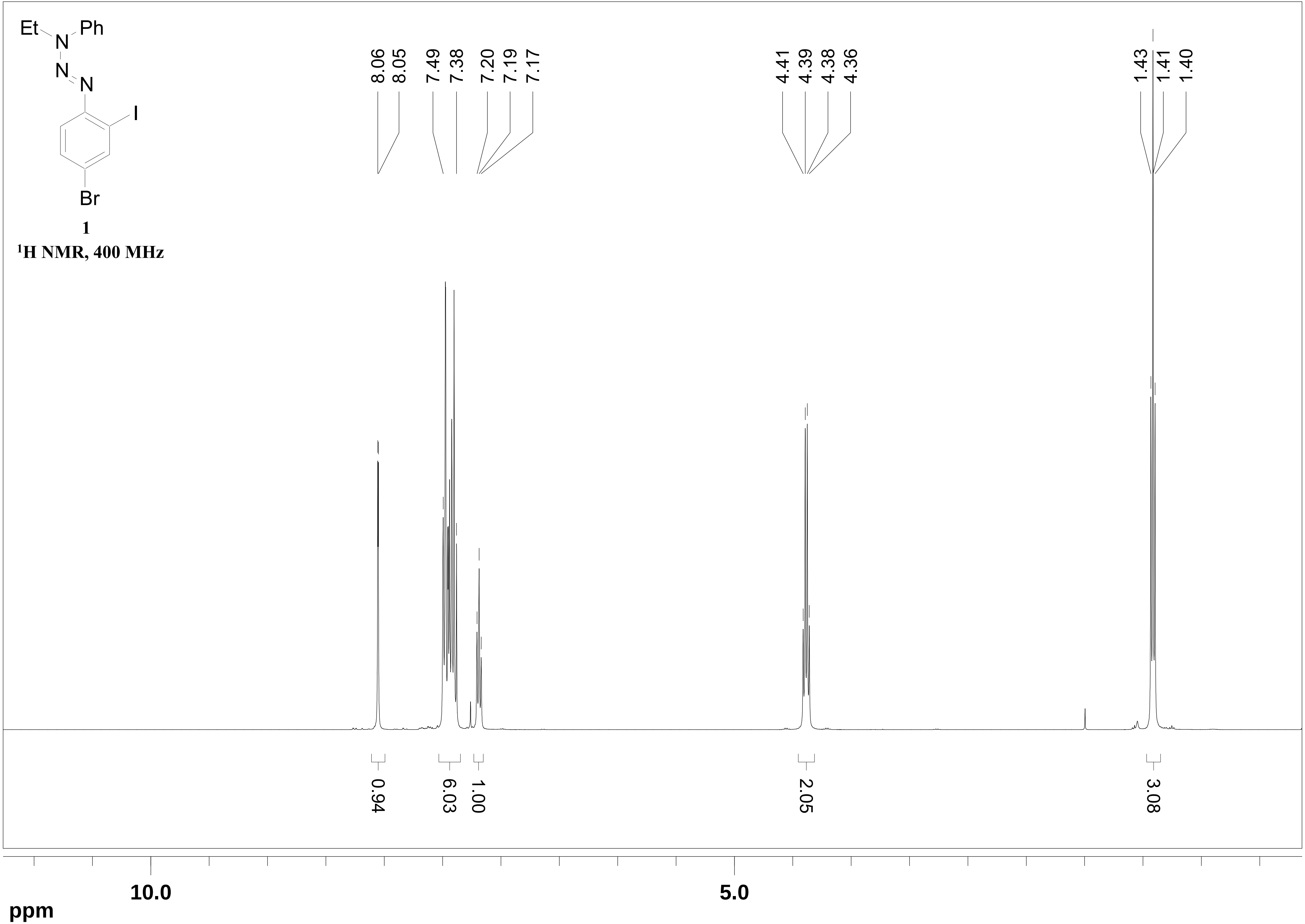

Et
N
Ph
N
N
I
Br
1
1H NMR, 400 MHz
8.06
8.05
7.49
7.38
7.20
7.19
7.17
4.41
4.39
4.38
4.36
1.43
1.41
1.40
0.94
6.03
1.00
2.05
3.08
10.0
5.0
ppm

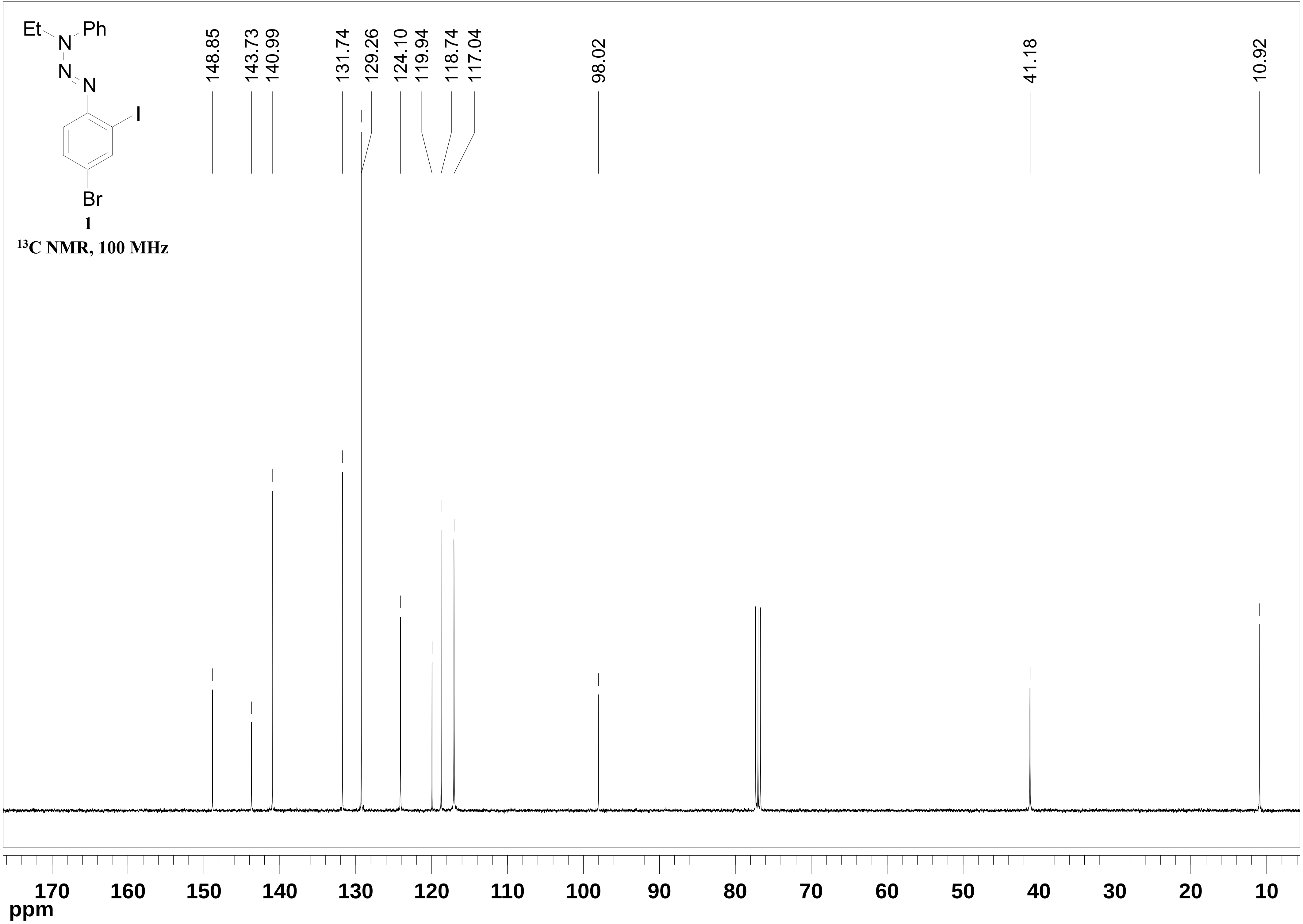
Et
N
Ph
N
N
N
I
Br
1
13C NMR, 100 MHz
148.85
143.73
140.99
131.74
129.26
124.10
119.94
118.74
117.04
98.02
41.18
10.92
170
160
150
140
130
120
110
100
90
80
70
60
50
40
30
20
10
ppm

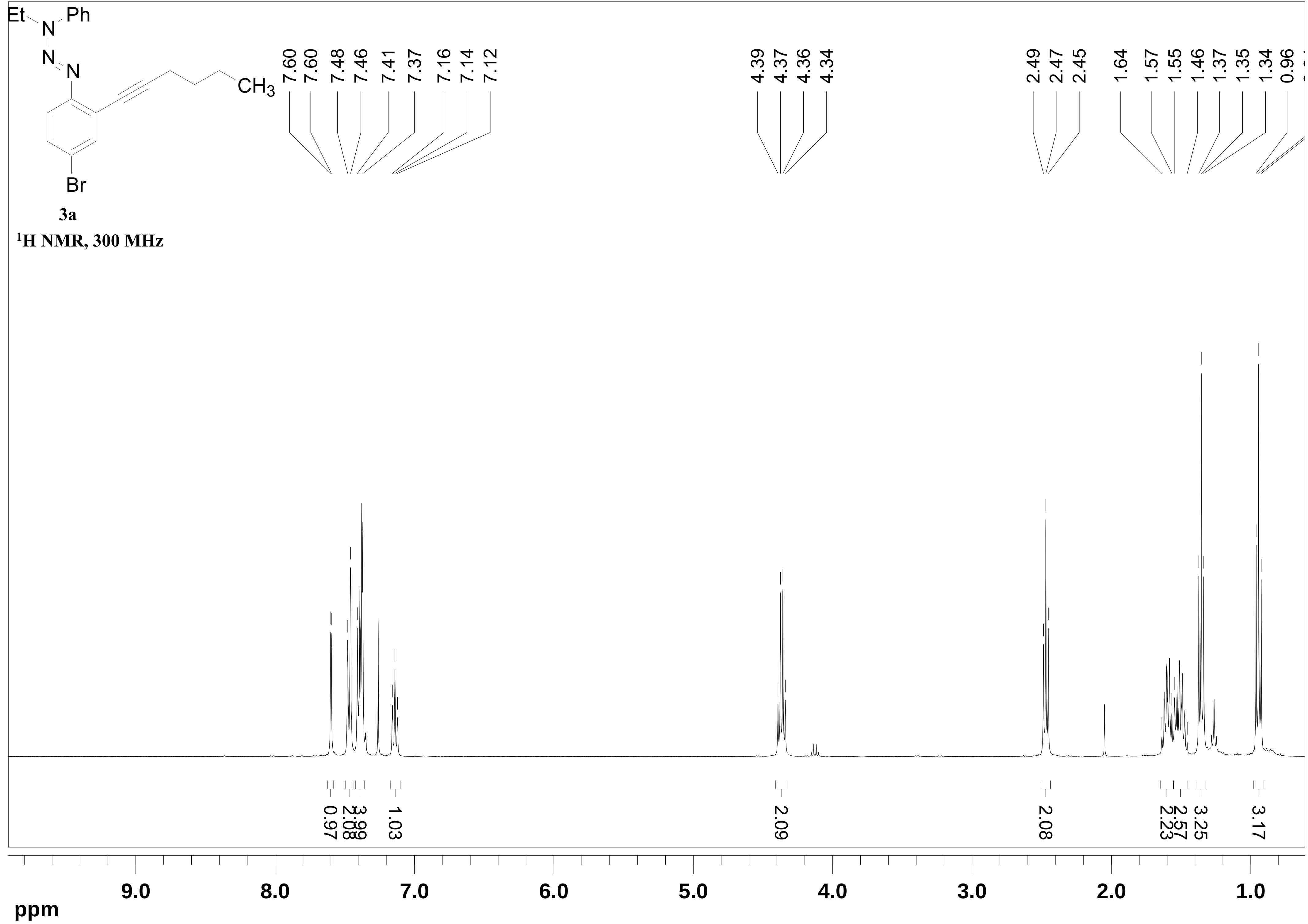

3a

^{1}H NMR, 300 MHz

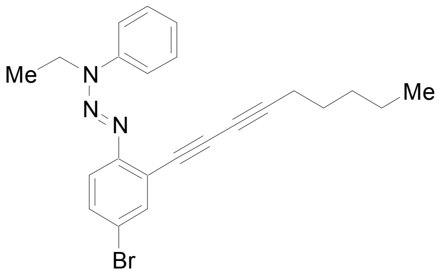

3b

^{1}H NMR, 300 MHz

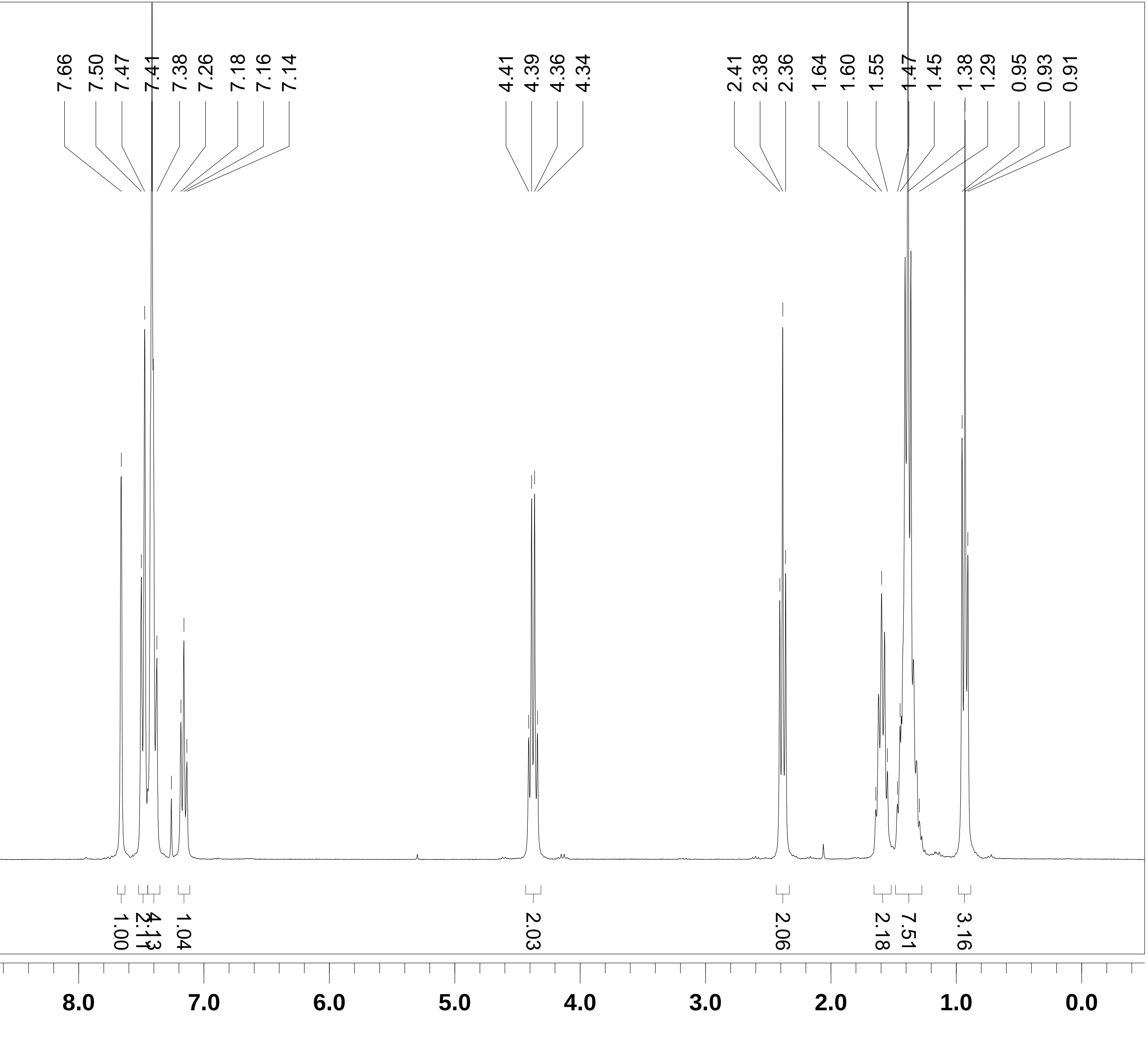

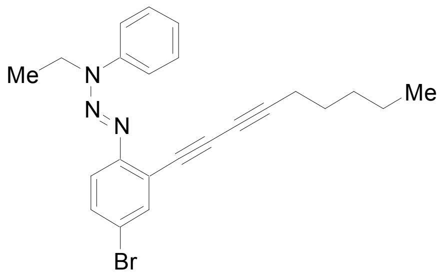

3b

^{13}C NMR, 75 MHz

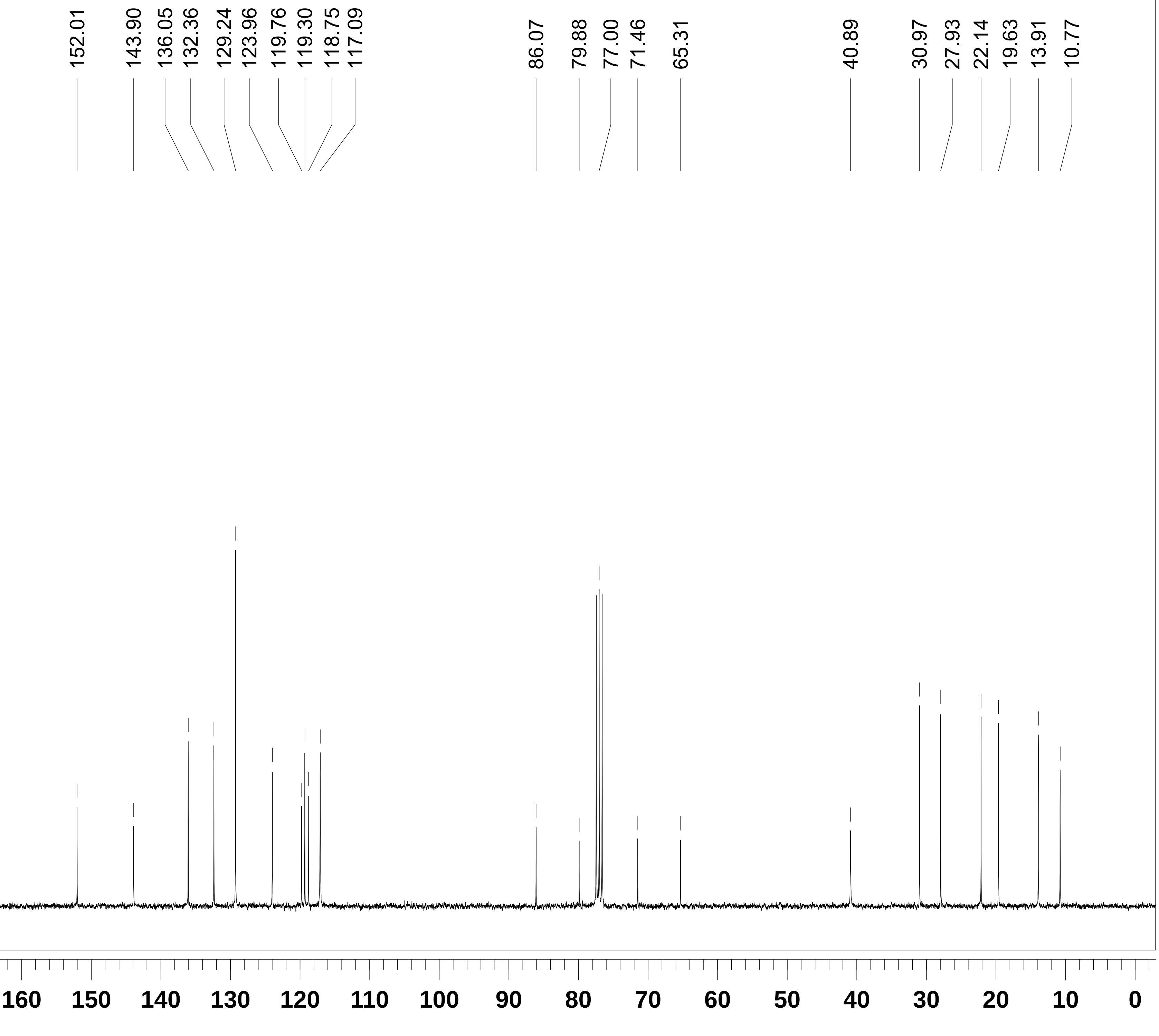

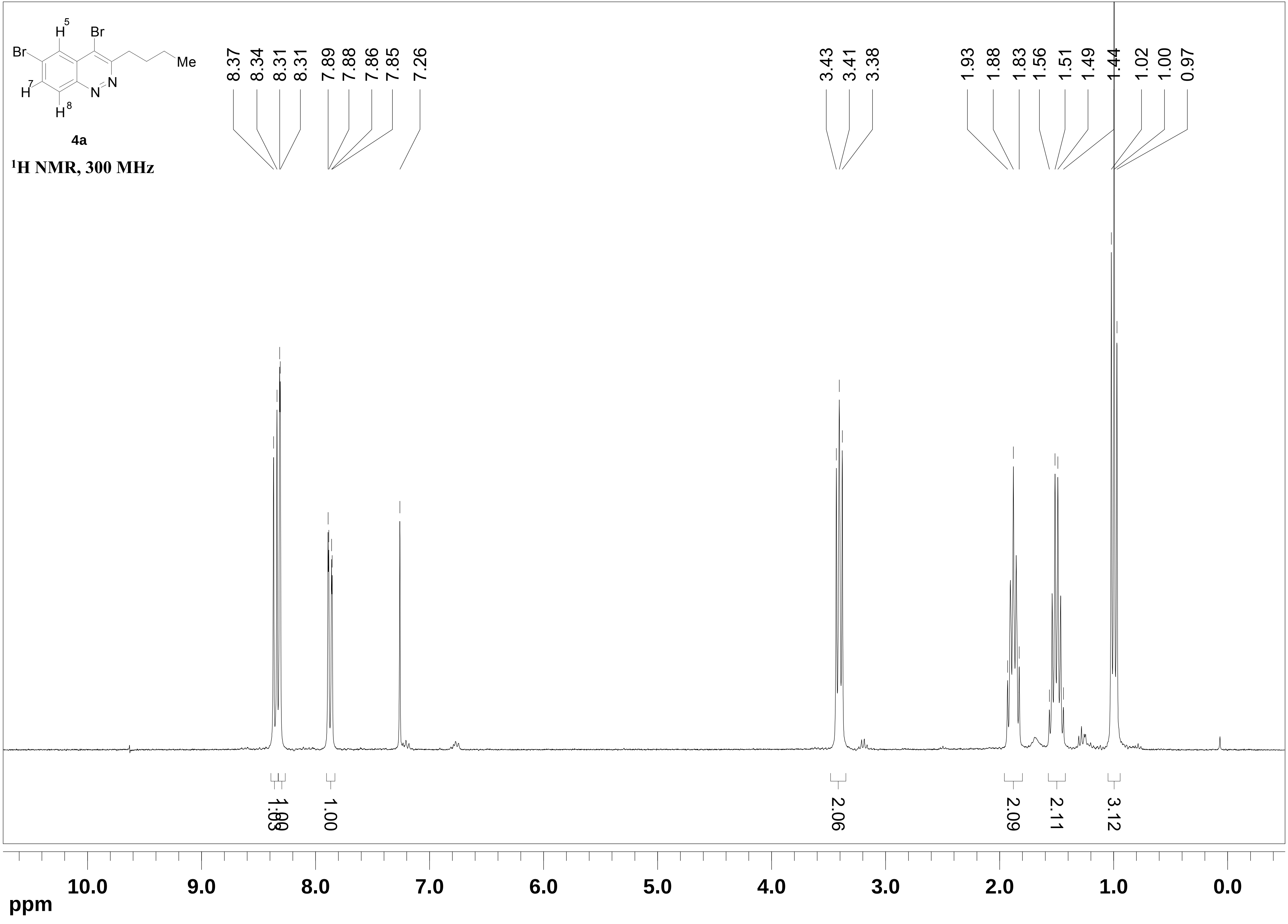

Br
Br
Me
N
N
H5
H7
H8
4a
¹H NMR, 300 MHz
8.37 8.34 8.31 8.31 7.89 7.88 7.86 7.85 7.26
3.43 3.41 3.38
1.93 1.88 1.83 1.56 1.51 1.49 1.44 1.02 1.00 0.97
1.03 1.00 1.00 2.06 2.09 2.11 3.12
ppm
10.0 9.0 8.0 7.0 6.0 5.0 4.0 3.0 2.0 1.0 0.0

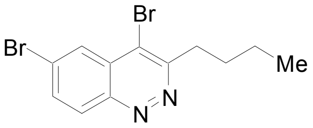

4a

^{13}C NMR, 75 MHz

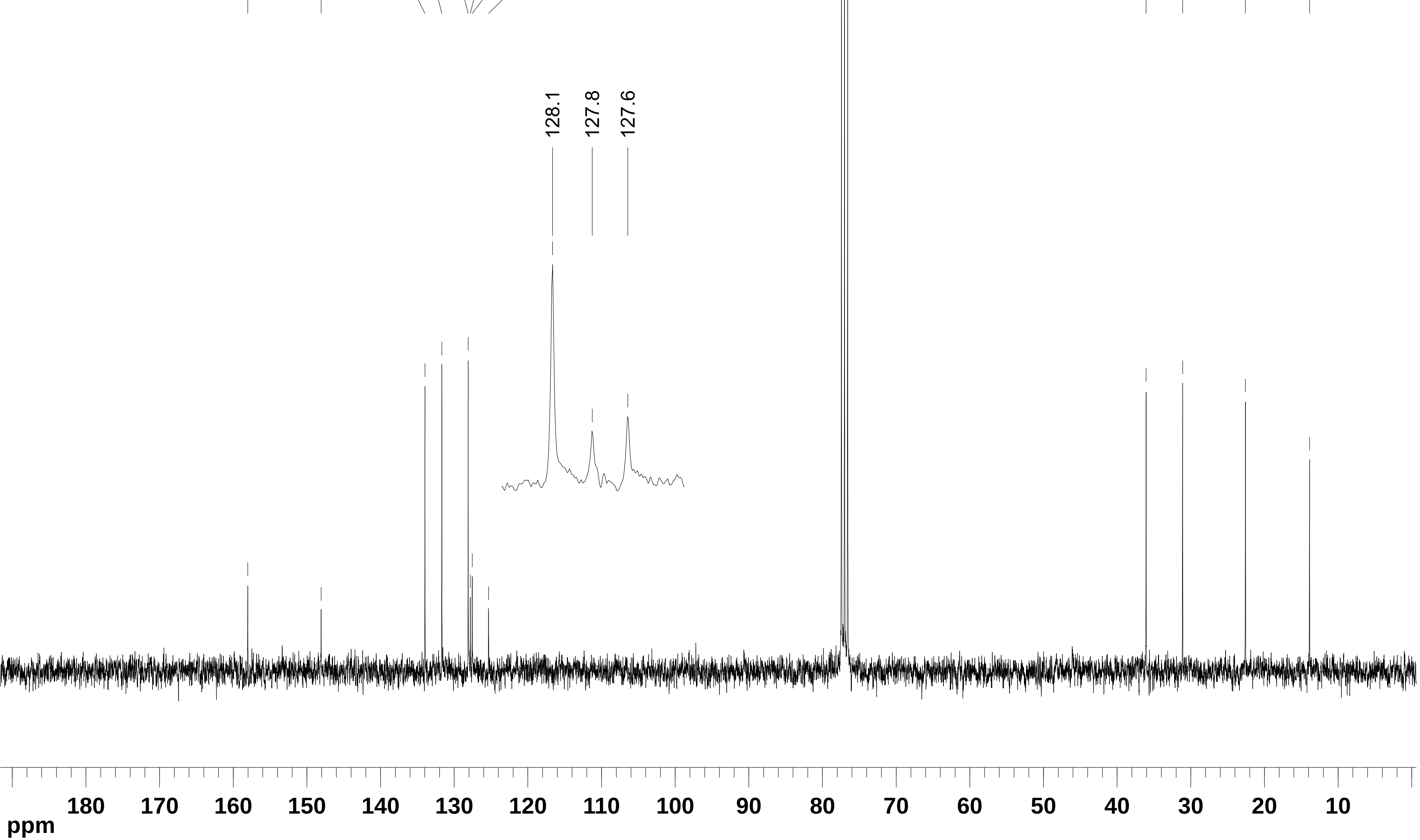

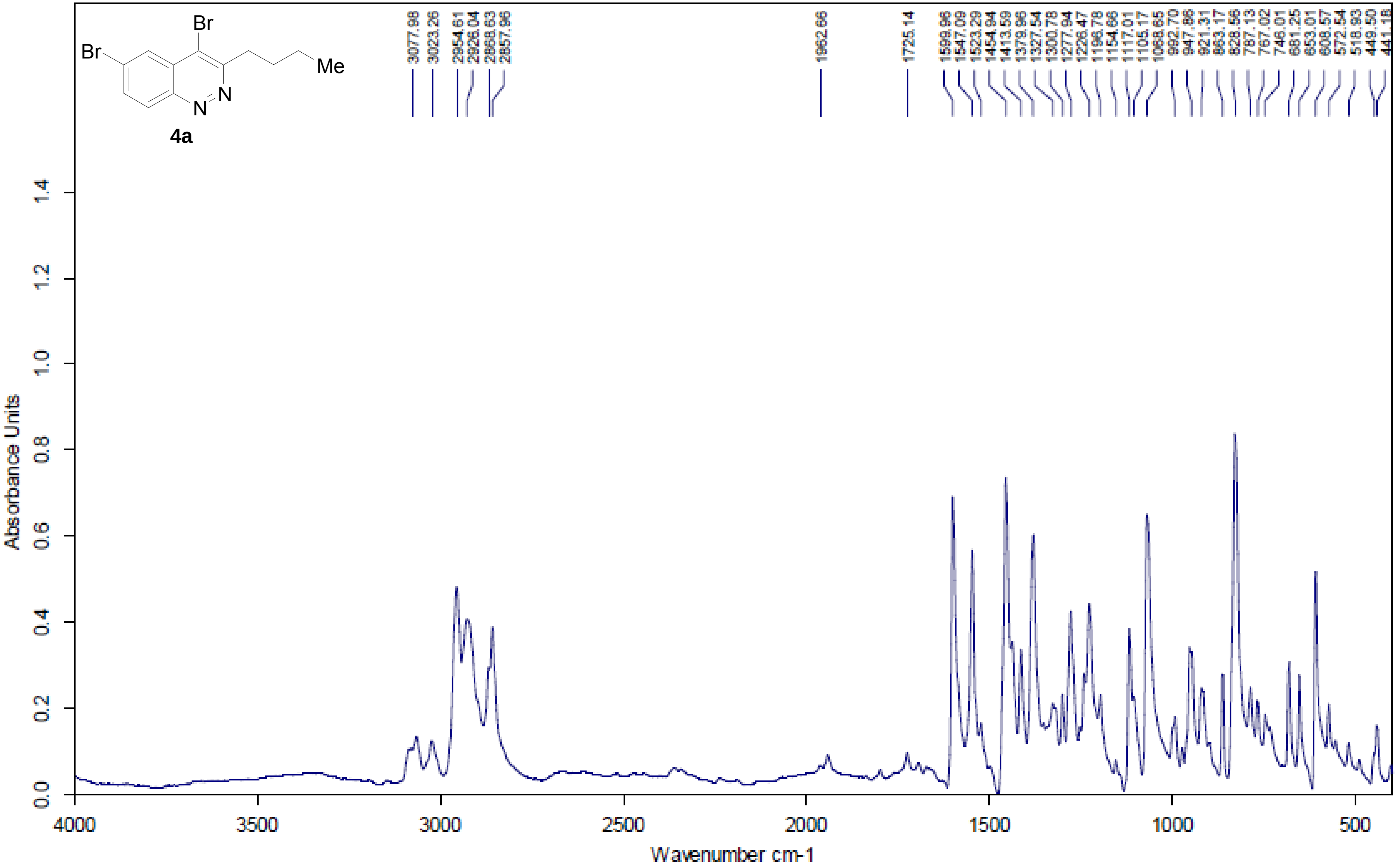

Br
Br
Me
N
N
4a
3077.98
3023.26
2954.61
2926.04
2868.63
2857.96
1962.66
1725.14
1599.96
1547.09
1523.29
1454.94
1413.59
1379.96
1327.54
1300.78
1277.94
1226.47
1196.78
1154.66
1117.01
1105.17
1068.65
992.70
947.86
921.31
863.17
828.56
787.13
767.02
746.01
681.25
653.01
608.57
572.54
518.93
449.50
441.18
Absorbance Units
0.0
0.2
0.4
0.6
0.8
1.0
1.2
1.4
4000
3500
3000
2500
2000
1500
1000
500
Wavenumber cm-1

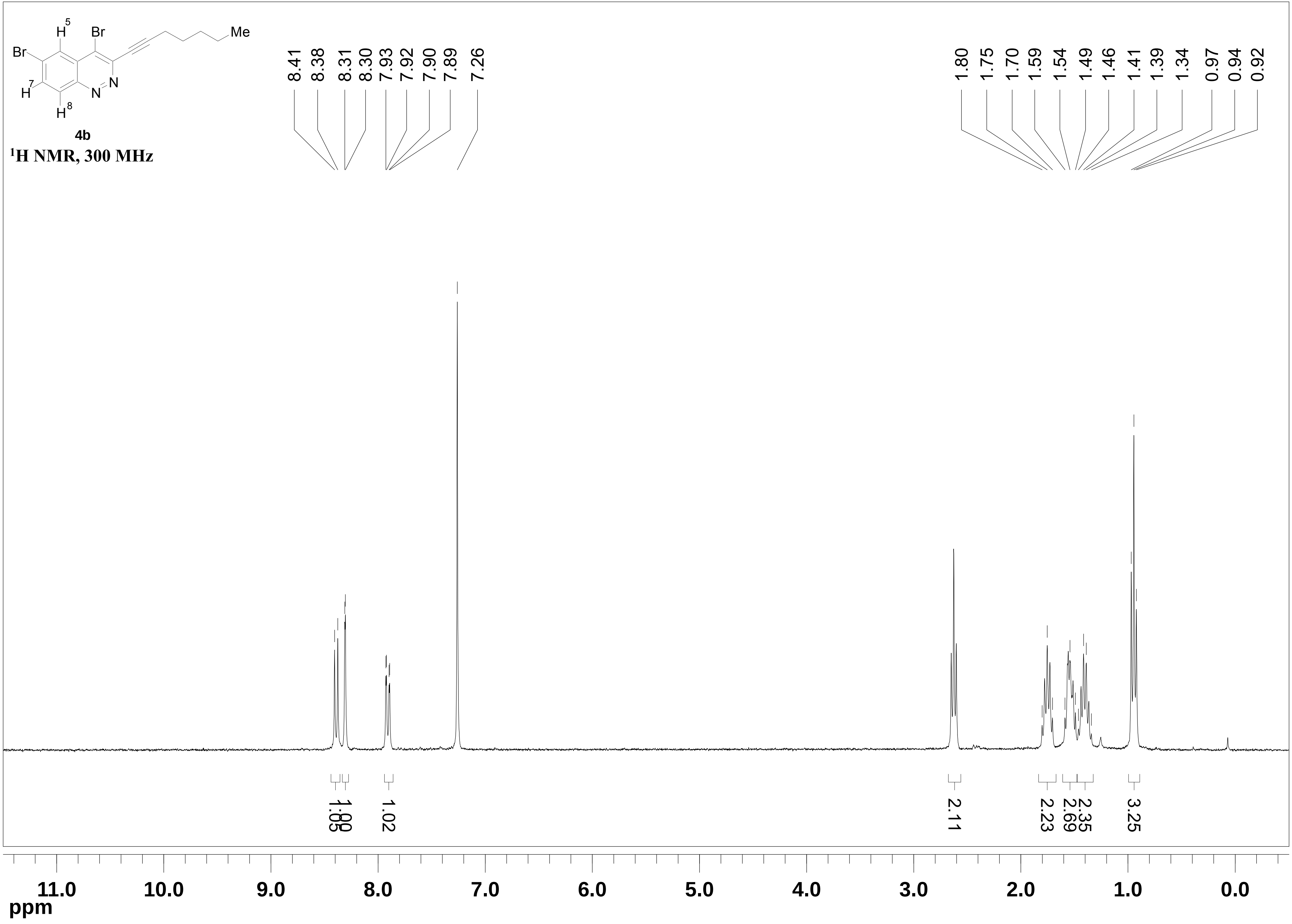
Br
Br
Me
H5
H7
H8
N
N
4b
1H NMR, 300 MHz
8.41 8.38 8.31 8.30 7.93 7.92 7.90 7.89 7.26
1.80 1.75 1.70 1.59 1.54 1.49 1.46 1.41 1.39 1.34 0.97 0.94 0.92
1.02
2.11
2.23 2.69 2.35
3.25
11.0 10.0 9.0 8.0 7.0 6.0 5.0 4.0 3.0 2.0 1.0 0.0
ppm

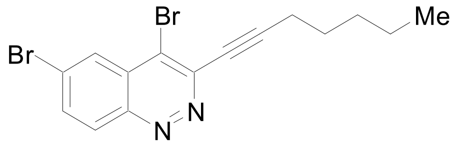

4b

^{13}C NMR, 75 MHz

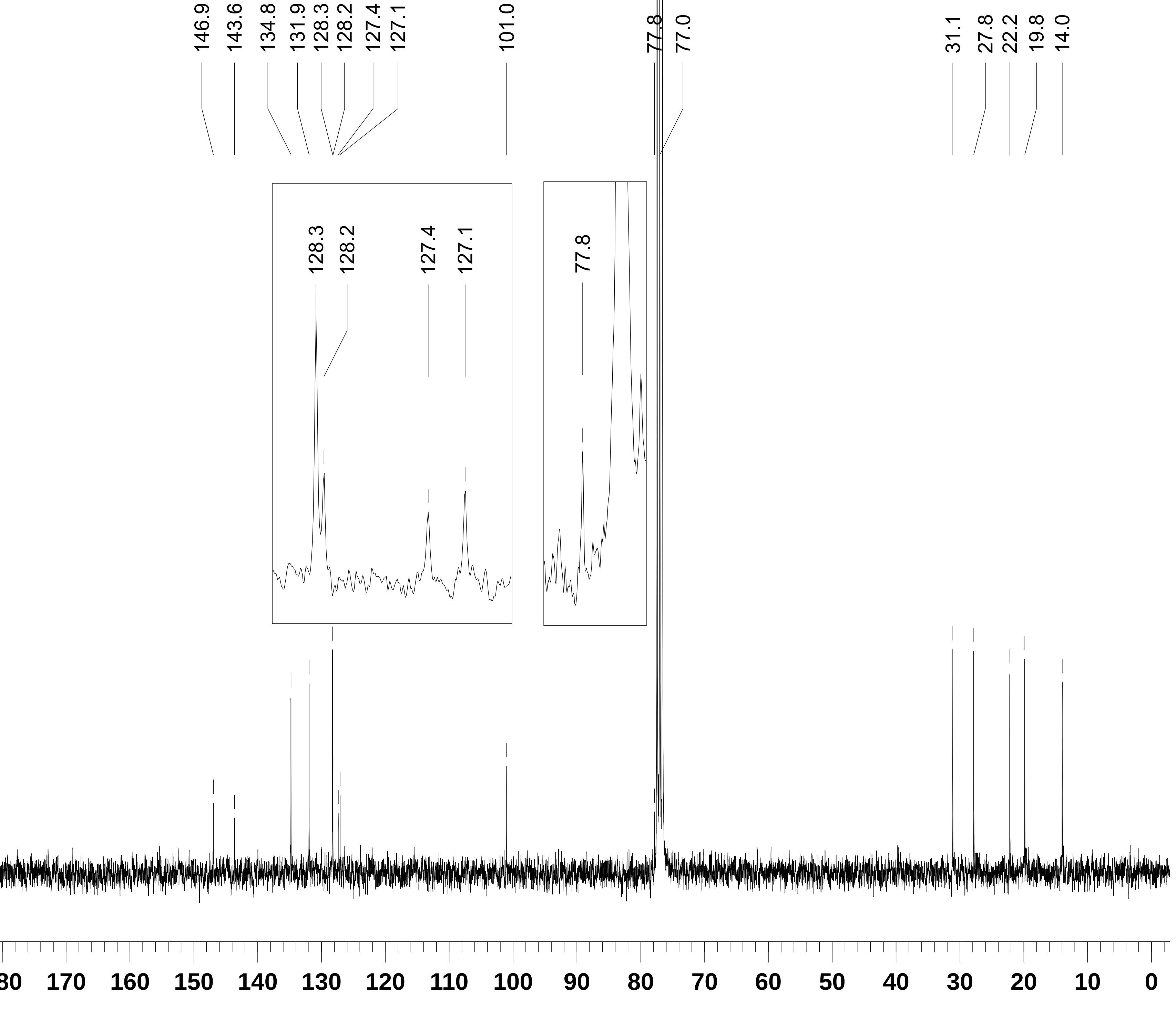

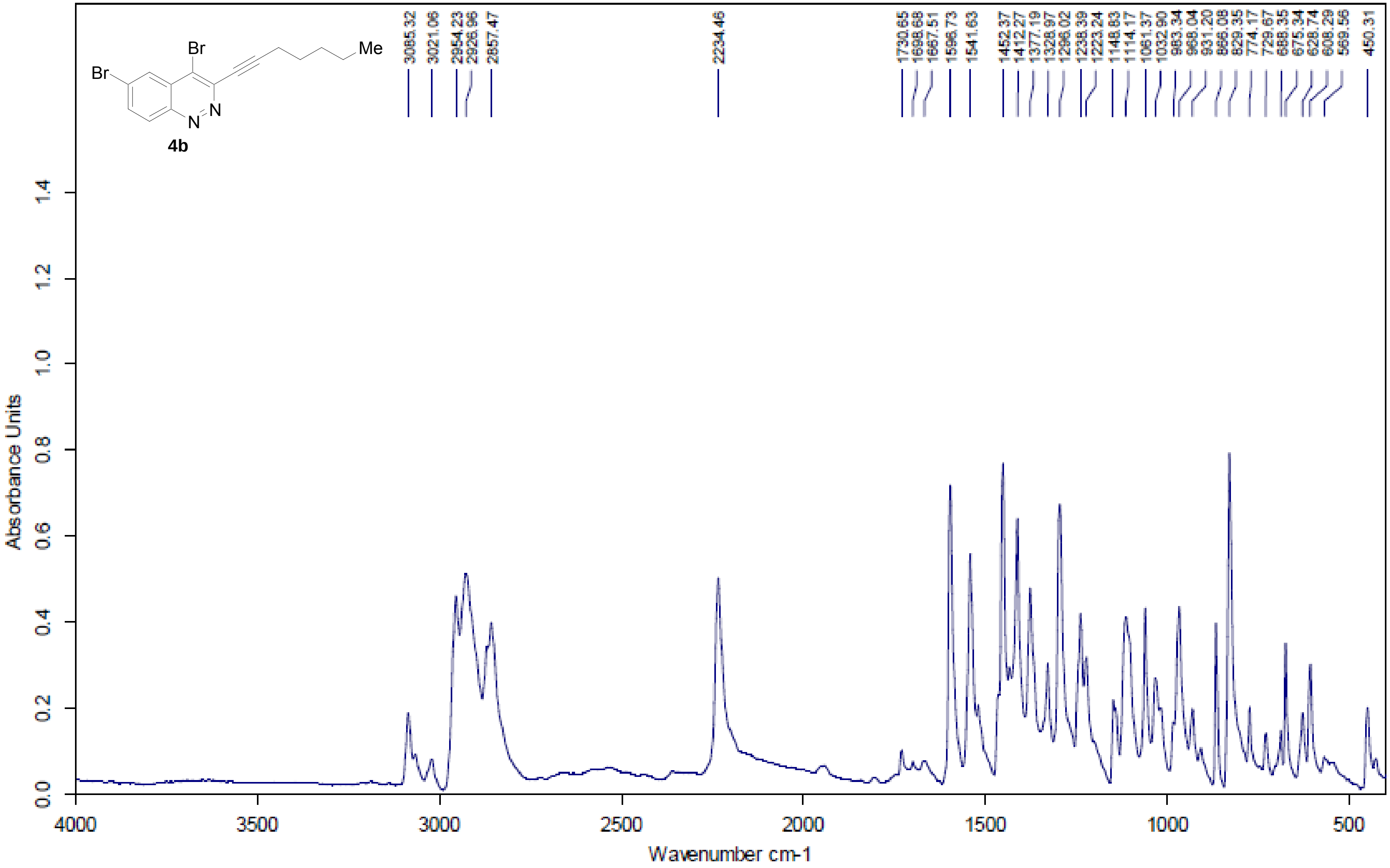

Br
Br
Me
N
N
4b
3085.32
3021.06
2954.23
2926.96
2857.47
2234.46
1730.65
1698.68
1667.51
1598.73
1541.63
1452.37
1412.27
1377.19
1328.97
1296.02
1238.39
1223.24
1148.83
1114.17
1061.37
1032.90
983.34
968.04
931.20
866.08
829.35
774.17
729.67
688.35
675.34
628.74
608.29
569.56
450.31
Absorbance Units
Wavenumber cm-1

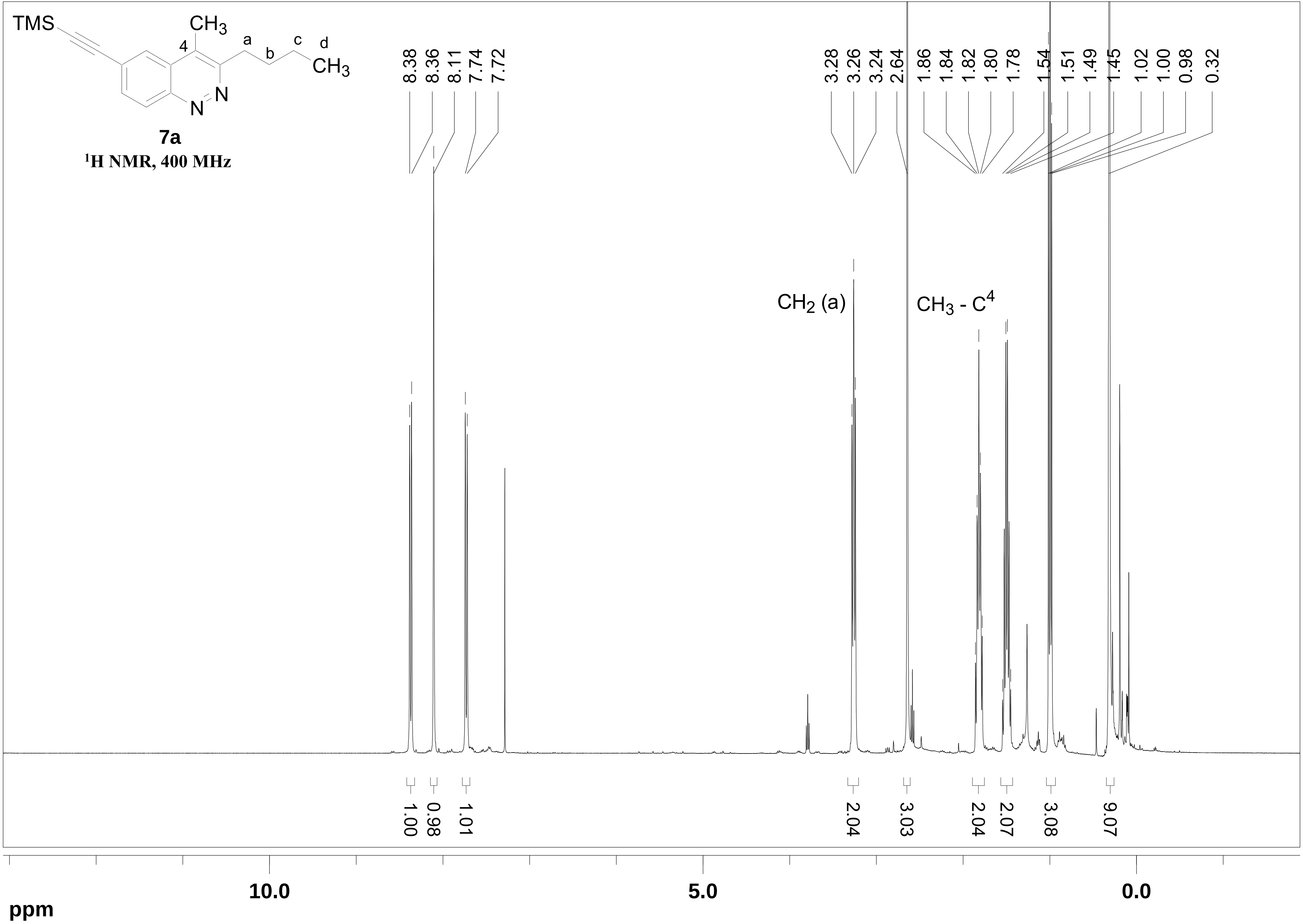

TMS
CH3
4
a
b
c
d
CH3
N
N
7a
1H NMR, 400 MHz
8.38 8.36 8.11 7.74 7.72
3.28 3.26 3.24 2.64 1.86 1.84 1.82 1.80 1.78 1.54 1.51 1.49 1.45 1.02 1.00 0.98 0.32
CH2 (a)
CH3 - C4
1.00 0.98 1.01 2.04 3.03 2.04 2.07 3.08 9.07
10.0
5.0
0.0
ppm

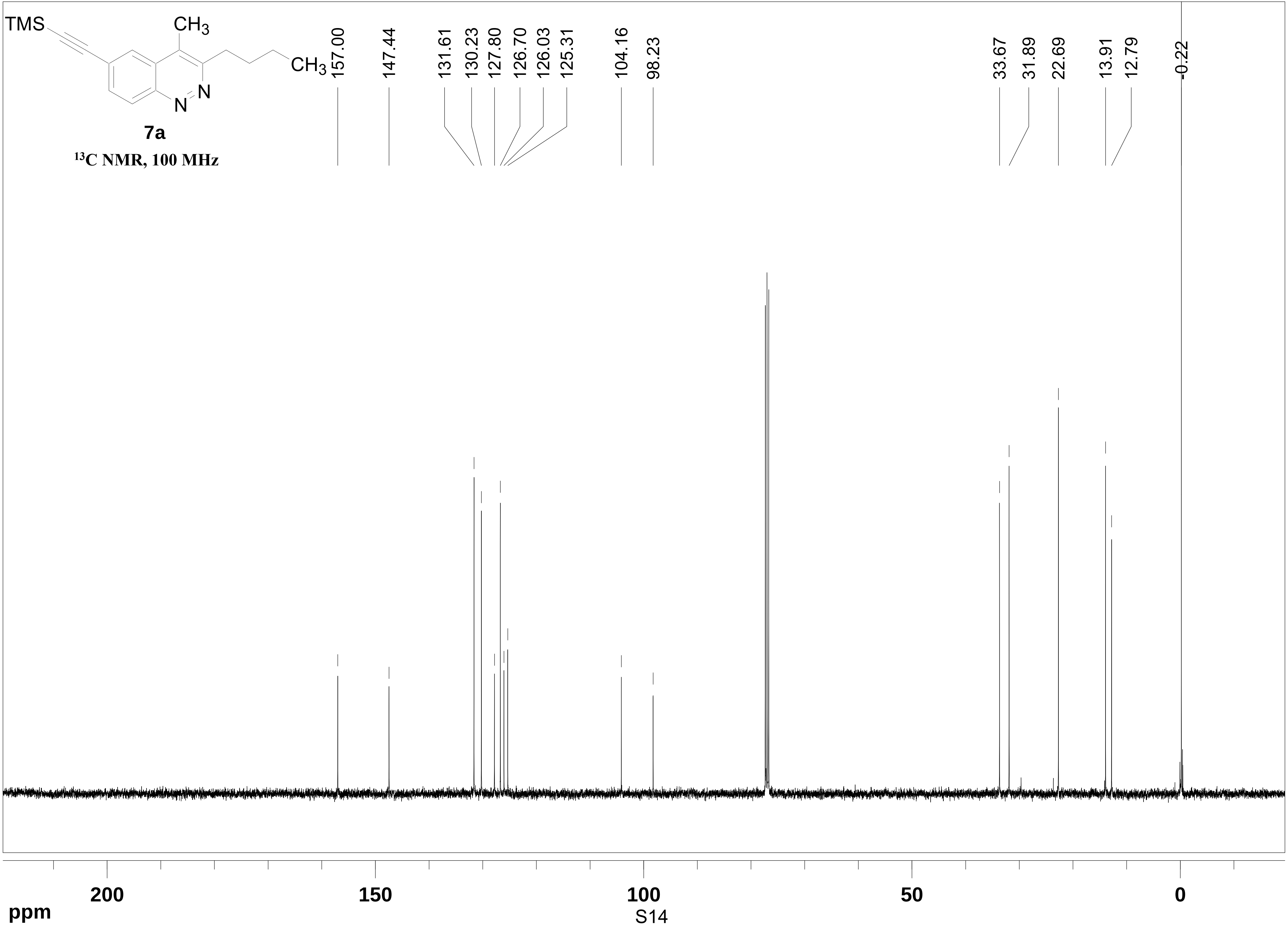

7a

^{13}C NMR, 100 MHz

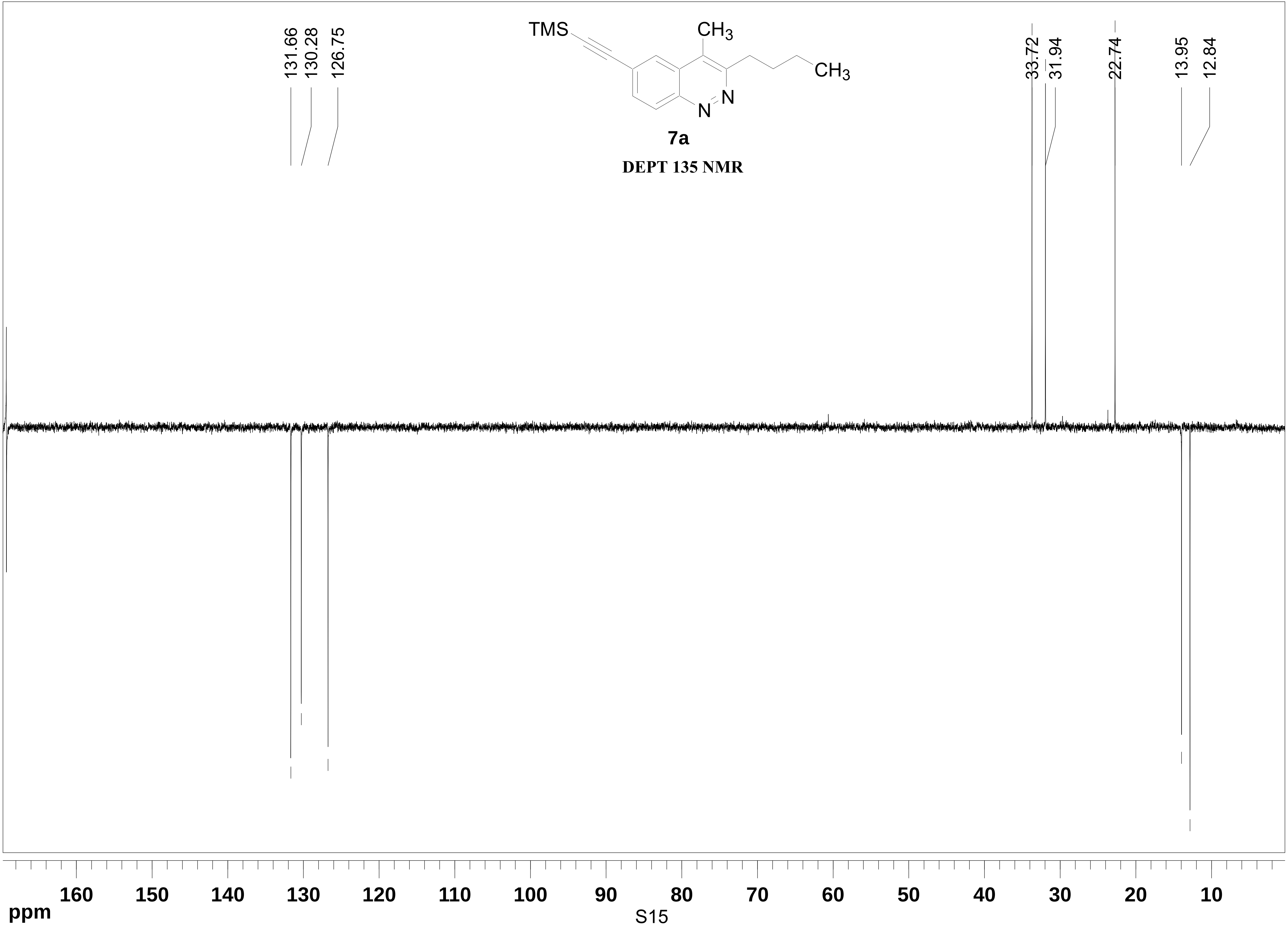

TMS
CH_3
CH_3
N
N
7a
DEPT 135 NMR
131.66
130.28
126.75
33.72
31.94
22.74
13.95
12.84
160
150
140
130
120
110
100
90
80
70
60
50
40
30
20
10
ppm

NOESY NMR

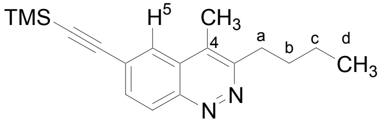

7a

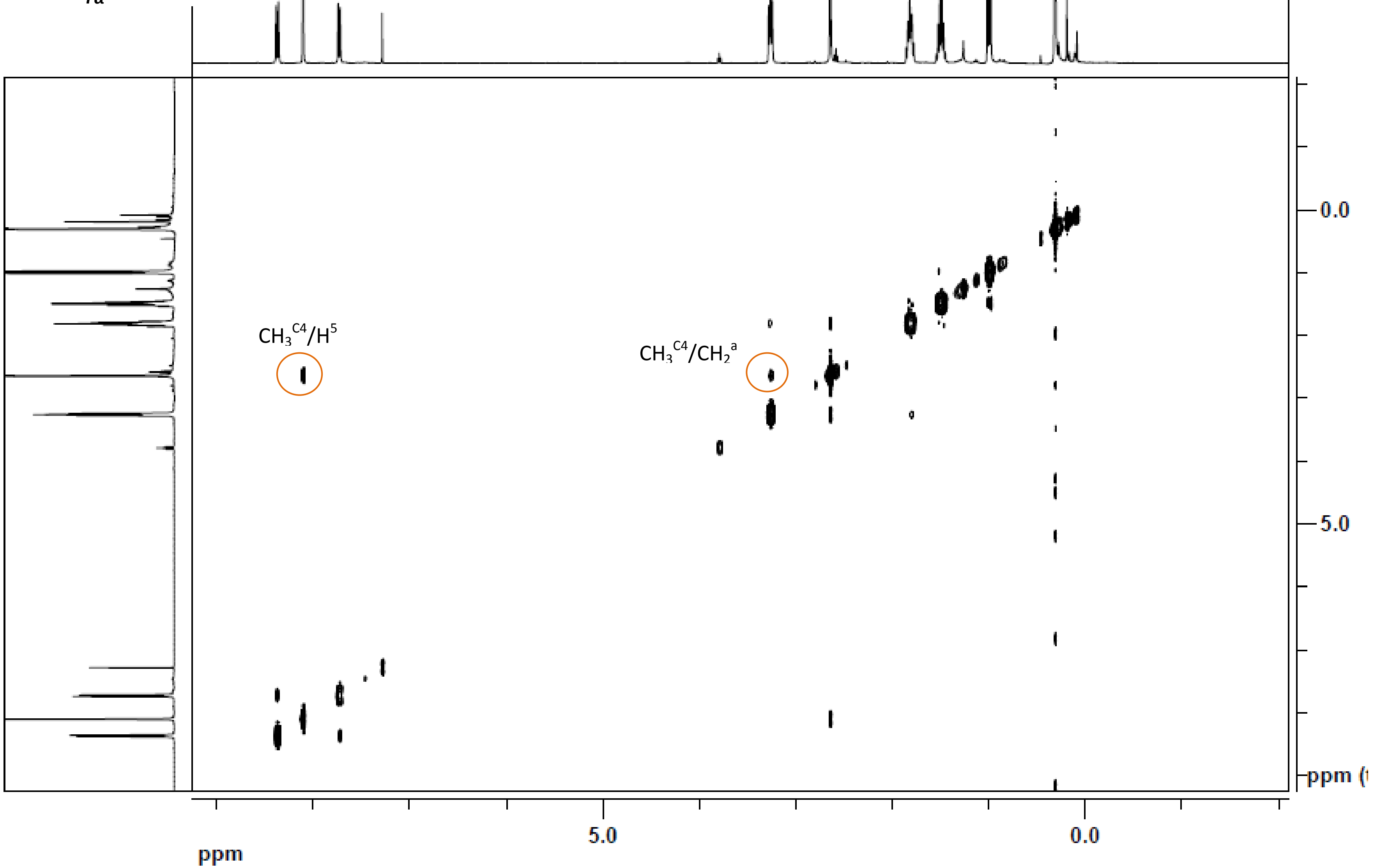

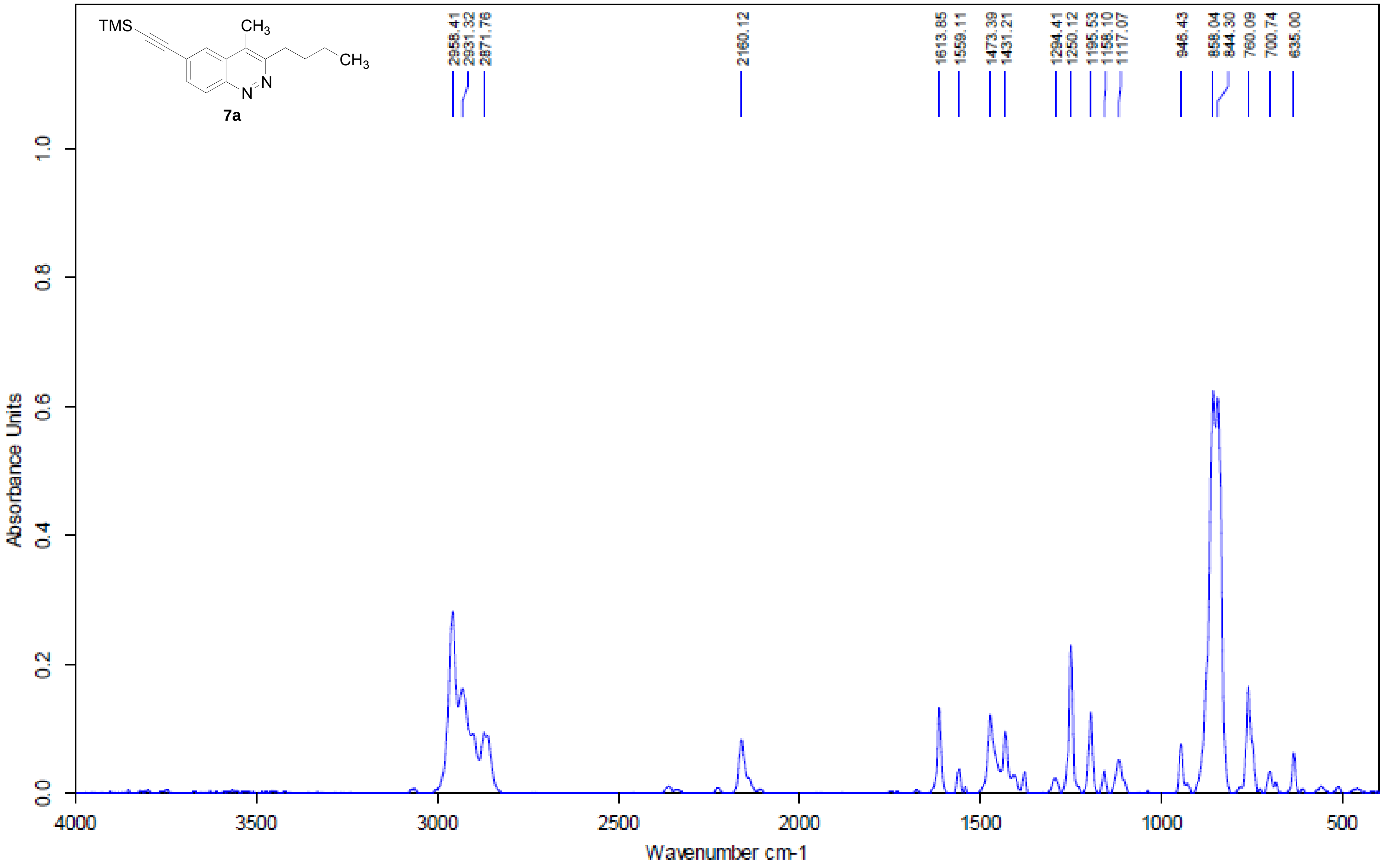

TMS
CH3
CH3
N
N
7a
2958.41
2931.32
2871.76
2160.12
1613.85
1559.11
1473.39
1431.21
1294.41
1250.12
1195.53
1158.10
1117.07
946.43
858.04
844.30
760.09
700.74
635.00
Absorbance Units
0.0
0.2
0.4
0.6
0.8
1.0
Wavenumber cm-1
4000
3500
3000
2500
2000
1500
1000
500

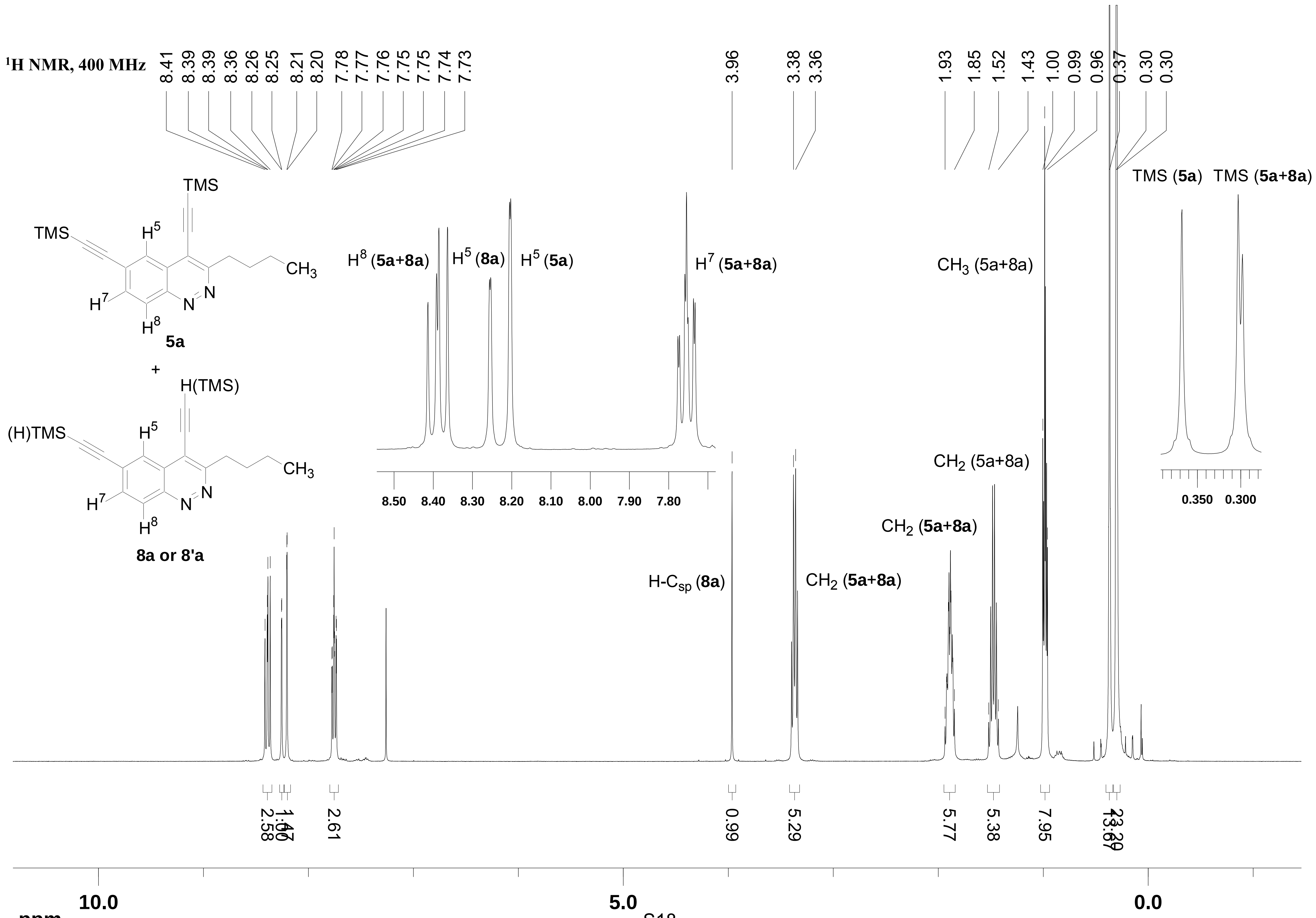

1H NMR, 400 MHz
8.41 8.39 8.39 8.36 8.26 8.25 8.21 8.20 7.78 7.77 7.76 7.75 7.75 7.74 7.73
3.96 3.38 3.36
1.93 1.85 1.52 1.43 1.00 0.99 0.96 0.37 0.30 0.30
TMS
TMS
H5
CH3
H7
N
N
H8
5a
+
H(TMS)
(H)TMS
H5
CH3
H7
N
N
H8
8a or 8'a
H8 (5a+8a)
H5 (8a)
H5 (5a)
H7 (5a+8a)
8.50 8.40 8.30 8.20 8.10 8.00 7.90 7.80
CH3 (5a+8a)
TMS (5a)
TMS (5a+8a)
0.350 0.300
CH2 (5a+8a)
CH2 (5a+8a)
H-Csp (8a)
CH2 (5a+8a)
2.58 1.00 1.47 2.61 0.99 5.29 5.77 5.38 7.95 43.20 4.67
10.0
5.0
0.0
ppm

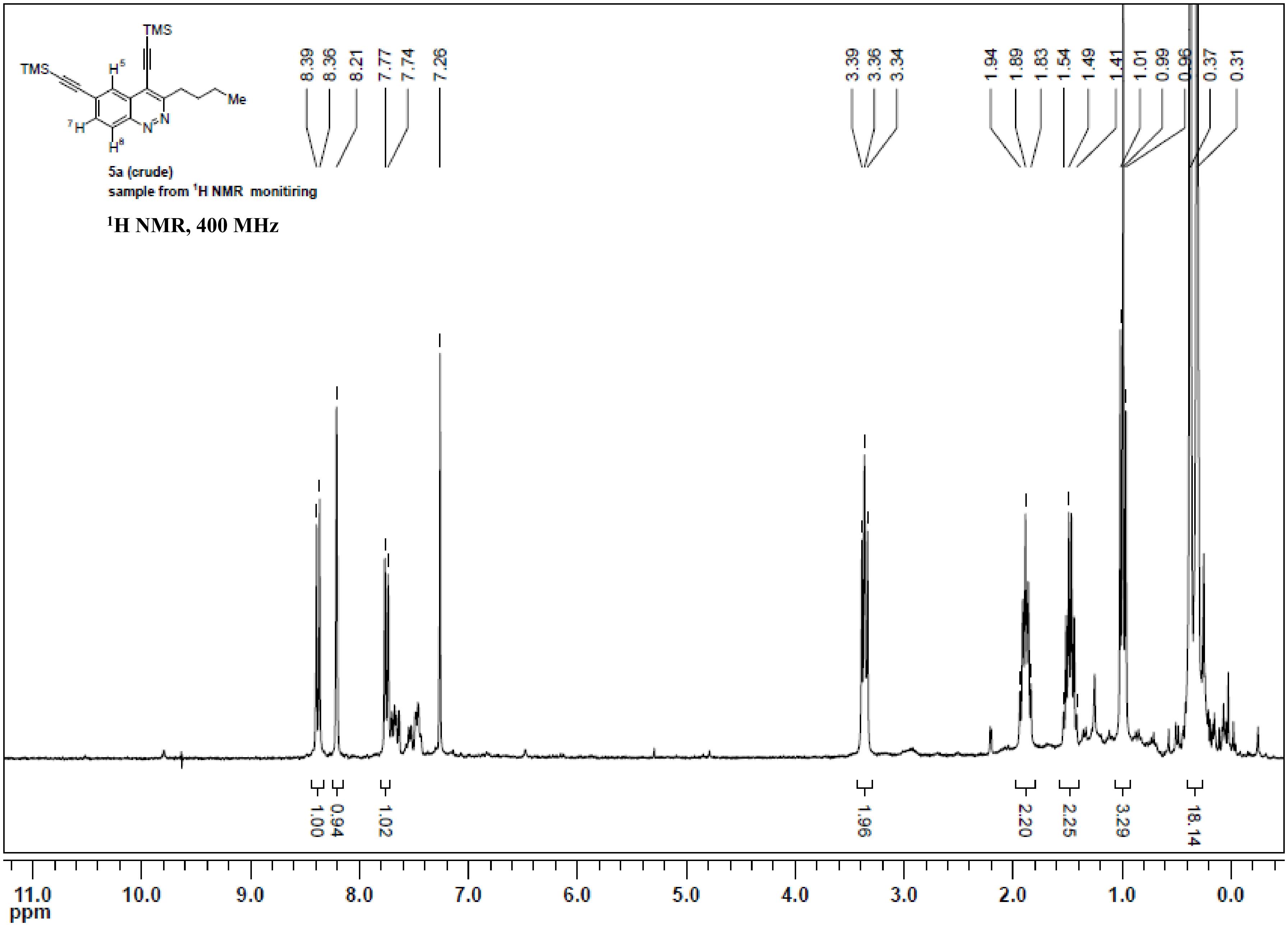
TMS
TMS
H5
7H
H8
Me
N
N
5a (crude)
sample from 1H NMR monitiring
1H NMR, 400 MHz
8.39
8.36
8.21
7.77
7.74
7.26
3.39
3.36
3.34
1.94
1.89
1.83
1.54
1.49
1.41
1.01
0.99
0.96
0.37
0.31
1.00
0.94
1.02
1.96
2.20
2.25
3.29
18.14
11.0
10.0
9.0
8.0
7.0
6.0
5.0
4.0
3.0
2.0
1.0
0.0
ppm

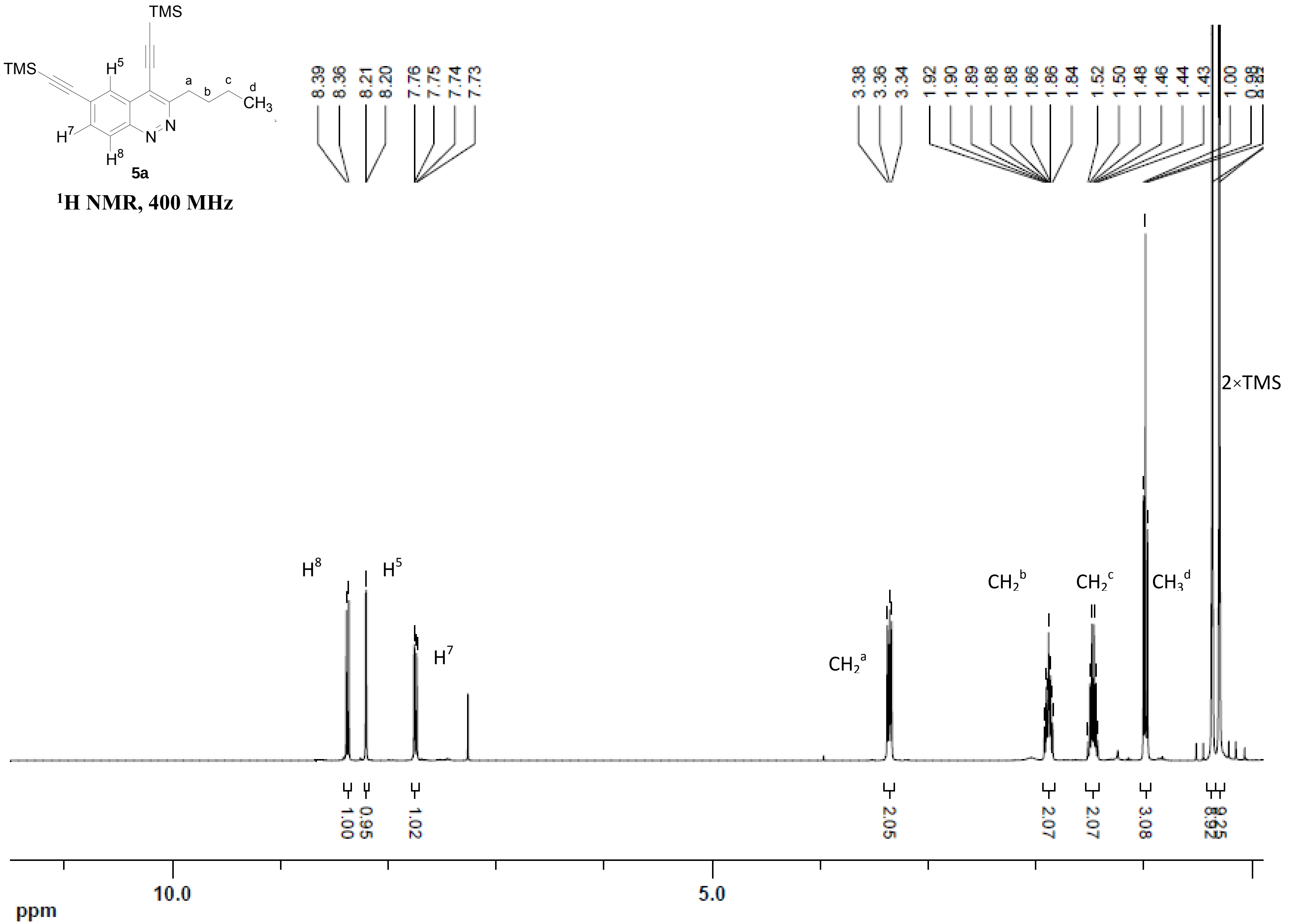
TMS
TMS
H5
H7
H8
a
b
c
d
CH3
N
N
5a
1H NMR, 400 MHz
8.39 8.36 8.21 8.20 7.76 7.75 7.74 7.73
3.38 3.36 3.34 1.92 1.90 1.89 1.88 1.88 1.86 1.86 1.84 1.52 1.50 1.48 1.46 1.44 1.43 1.00
H8
H5
H7
CH2a
CH2b
CH2c
CH3d
2×TMS
1.00 0.95 1.02 2.05 2.07 2.07 3.08
10.0
5.0
ppm

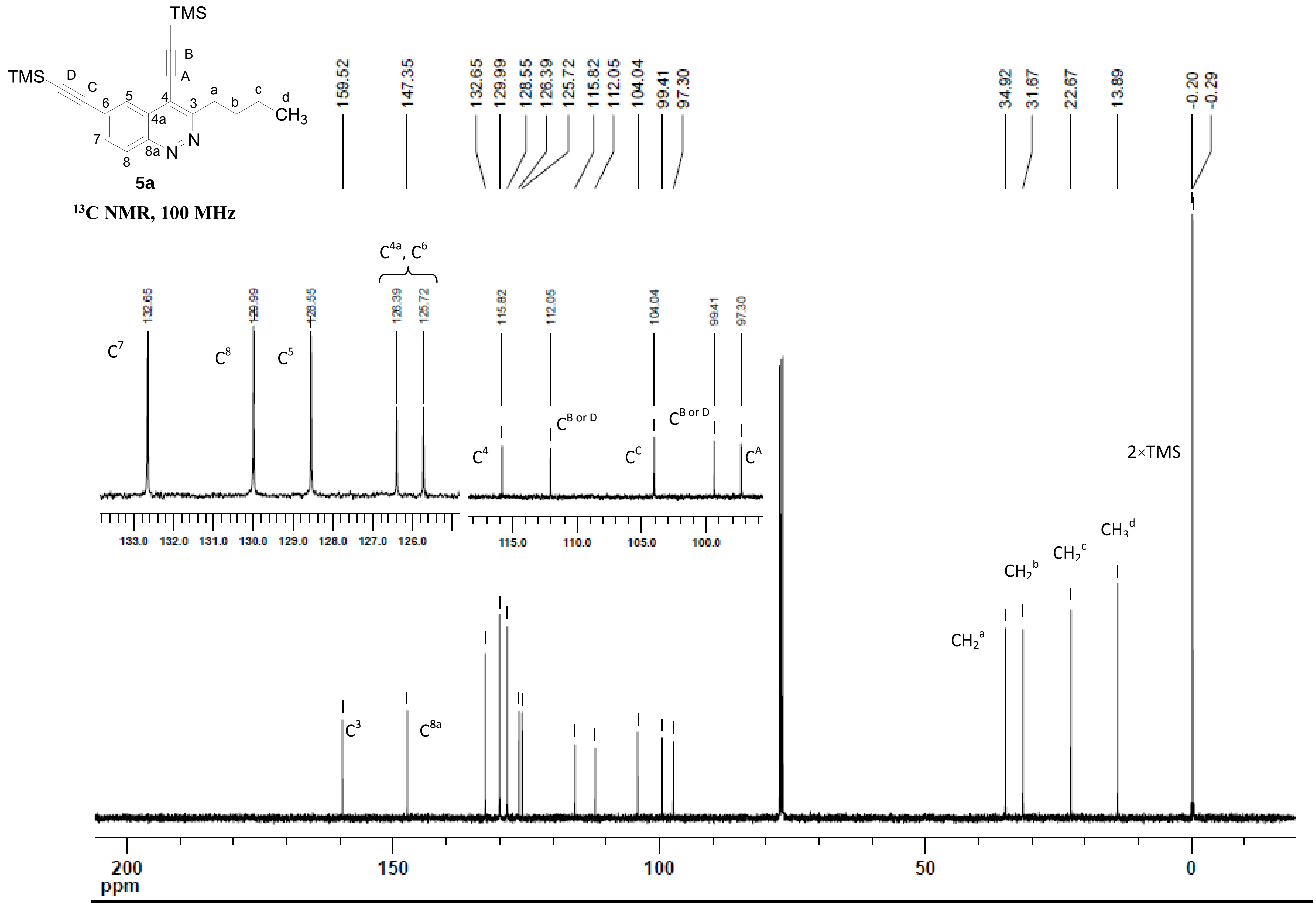

TMS
TMS
A
B
C
D
a
b
c
d
CH_3
3
4
4a
5
6
7
8
8a
N
N
5a
^{13}C NMR, 100 MHz
159.52
147.35
132.65
129.99
128.55
126.39
125.72
115.82
112.05
104.04
99.41
97.30
34.92
31.67
22.67
13.89
-0.20
-0.29
C^{4a}, C^6
C^7
C^8
C^5
C^4
$C^{B\ or\ D}$
C^C
$C^{B\ or\ D}$
C^A
2×TMS
CH_3^d
CH_2^c
CH_2^b
CH_2^a
C^3
C^{8a}
133.0
132.0
131.0
130.0
129.0
128.0
127.0
126.0
115.0
110.0
105.0
100.0
200
150
100
50
0
ppm

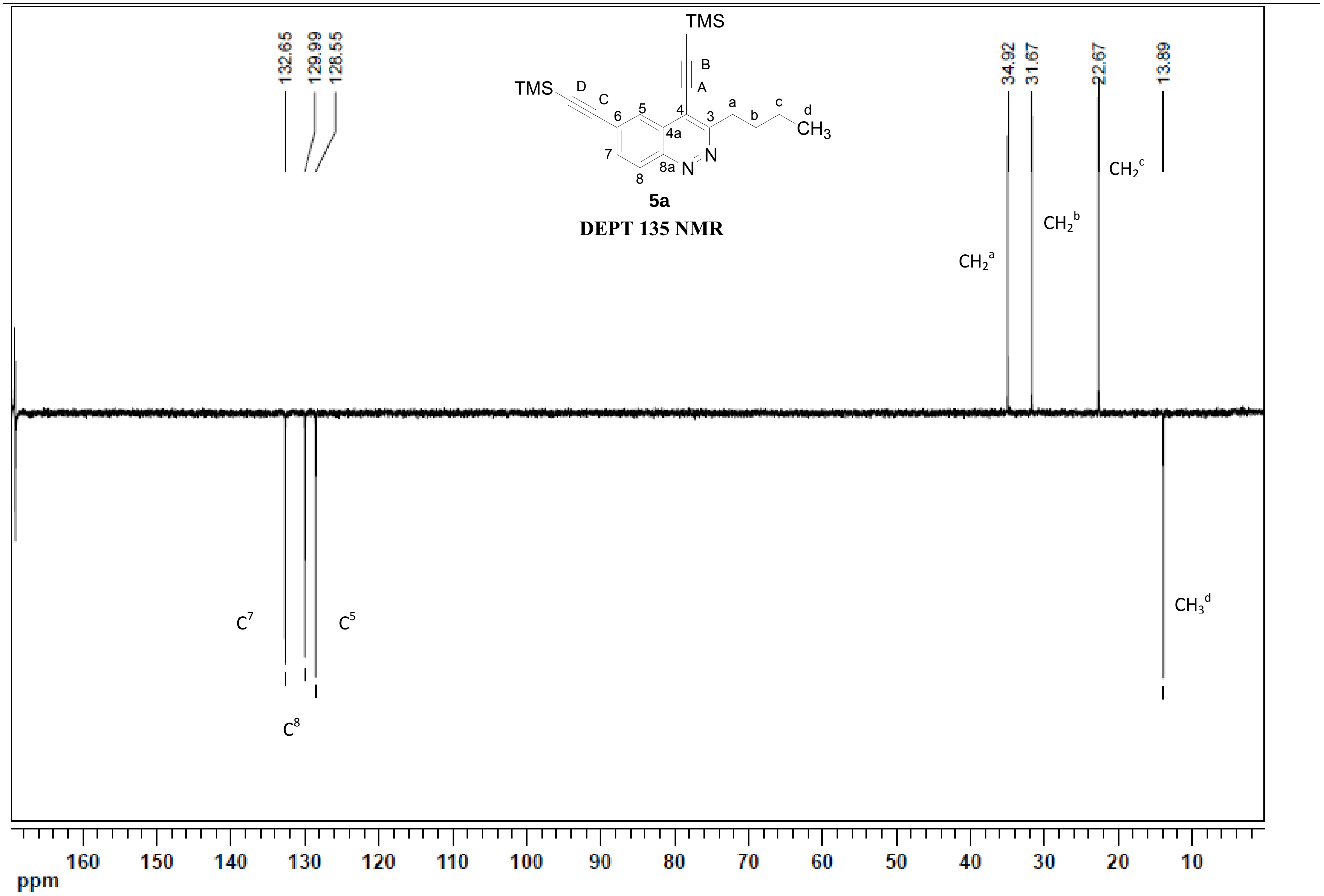

TMS
B
A
TMS
D
C
5
4
a
b
c
d
3
6
4a
CH3
7
N
N
8a
8
5a
DEPT 135 NMR
132.65
129.99
128.55
34.92
31.67
22.67
13.89
CH2c
CH2b
CH2a
C7
C5
C8
CH3d
160
150
140
130
120
110
100
90
80
70
60
50
40
30
20
10
ppm

HSQC NMR

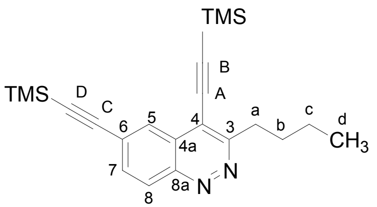

5a

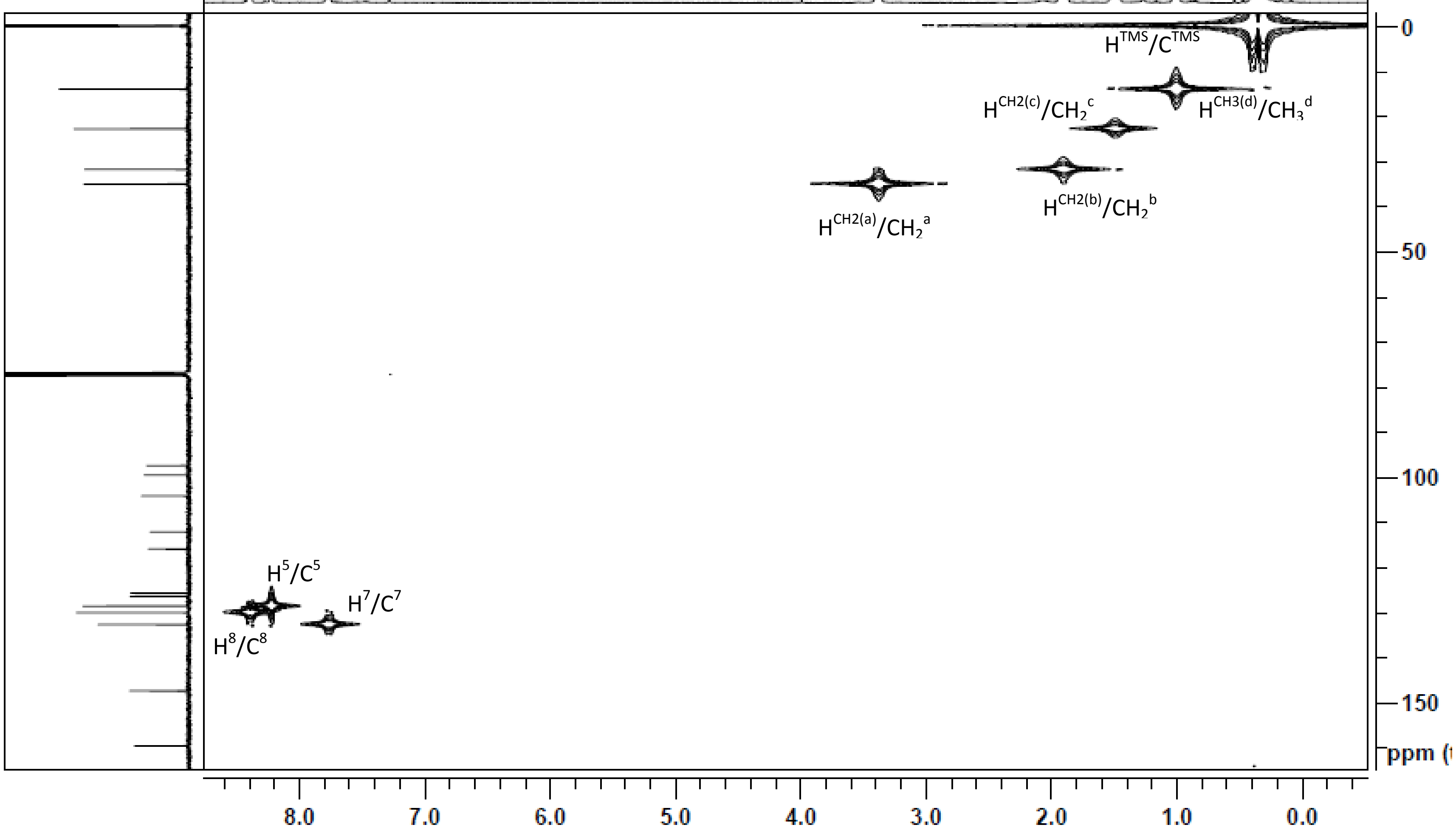

H^TMS/C^TMS
H^CH2(c)/CH2^c
H^CH3(d)/CH3^d
H^CH2(a)/CH2^a
H^CH2(b)/CH2^b
H^5/C^5
H^7/C^7
H^8/C^8
ppm

HMBC NMR

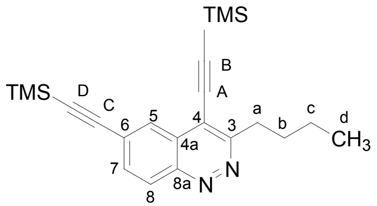
TMS
B
A
TMS
D
C
5
4
a
b
c
d
CH_3
6
4a
3
7
N
8
8a
N

5a

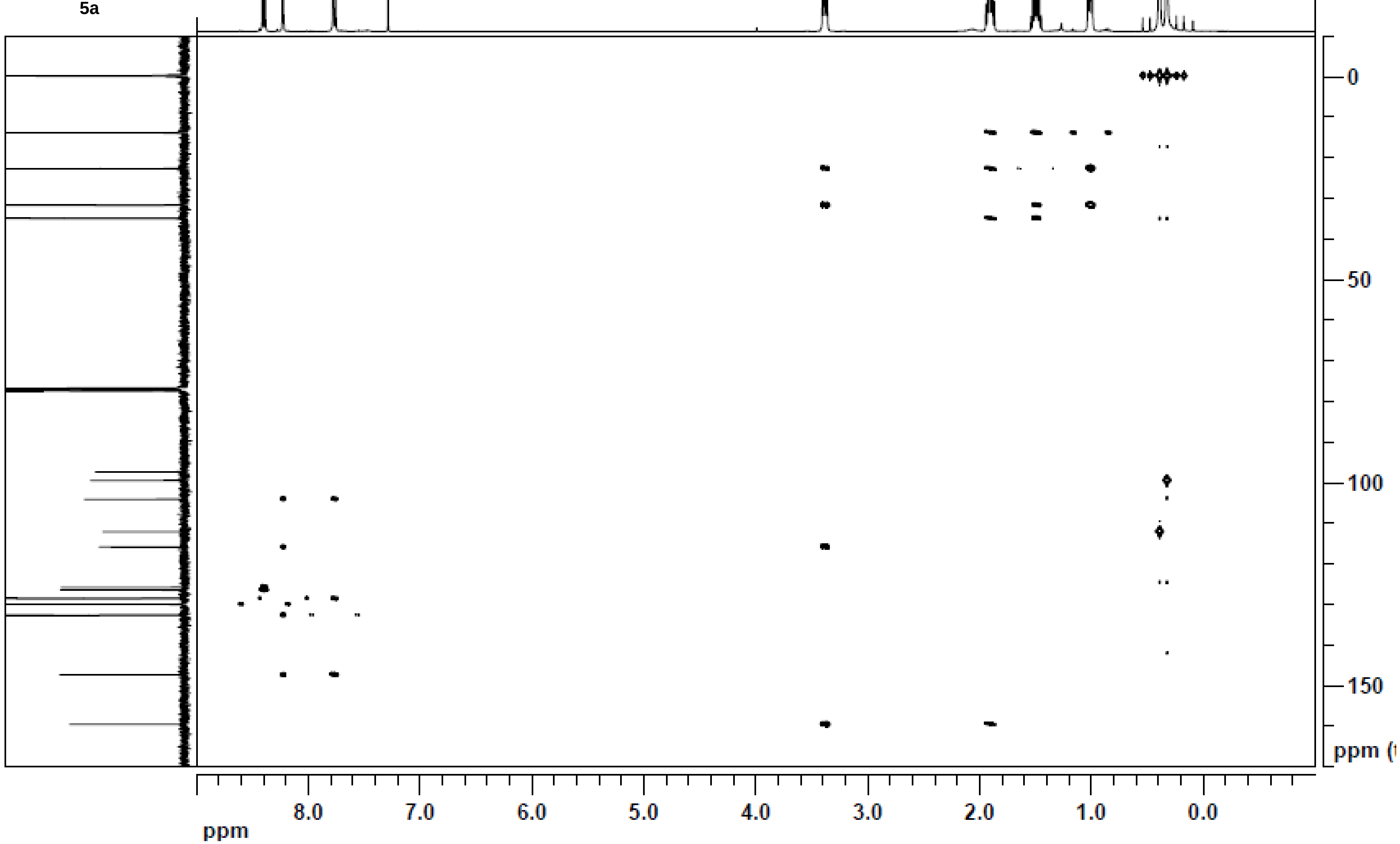
0
50
100
150
ppm
8.0
7.0
6.0
5.0
4.0
3.0
2.0
1.0
0.0
ppm

HMBC NMR (Zoom 1)

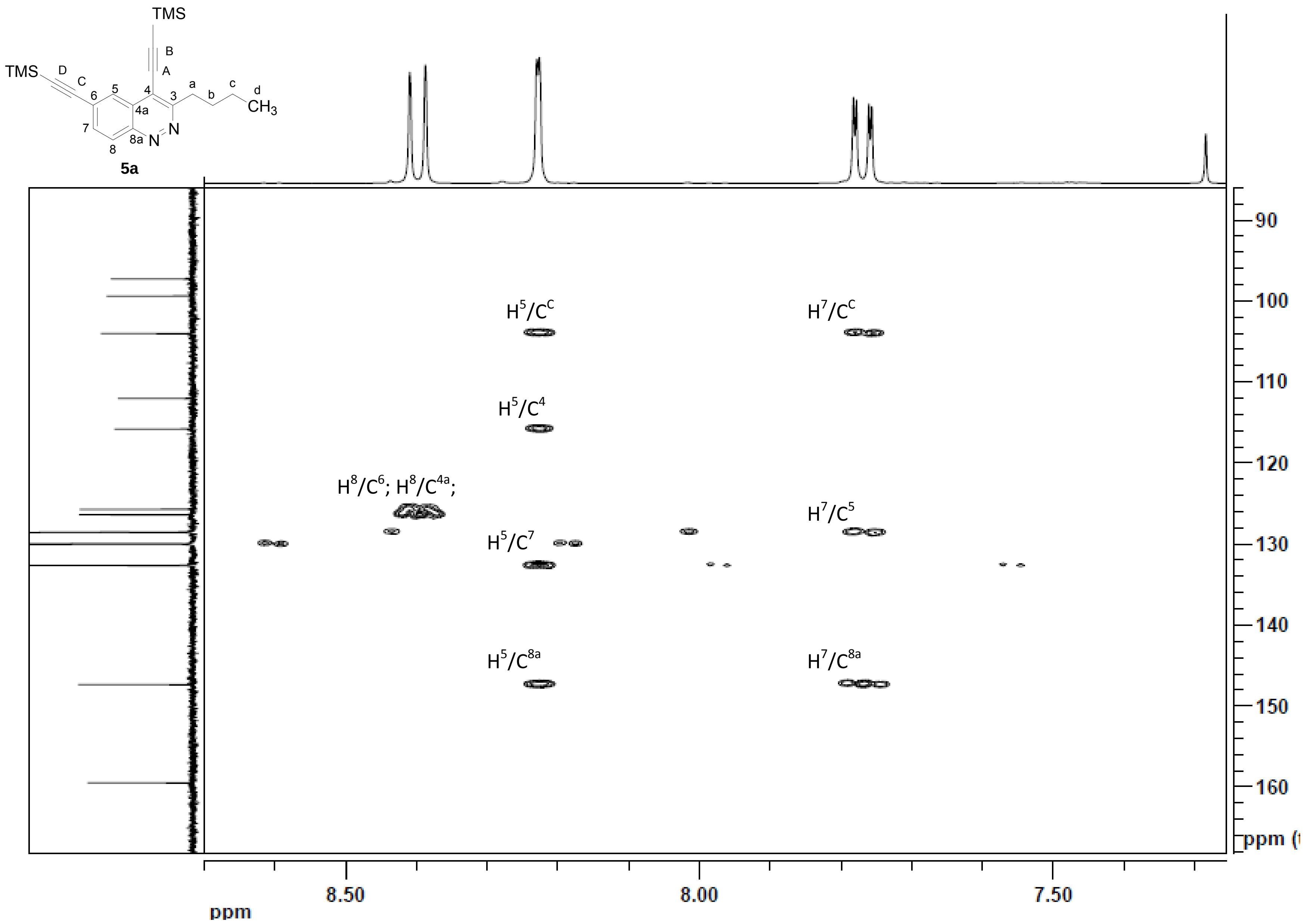

HMBC NMR (Zoom_1)

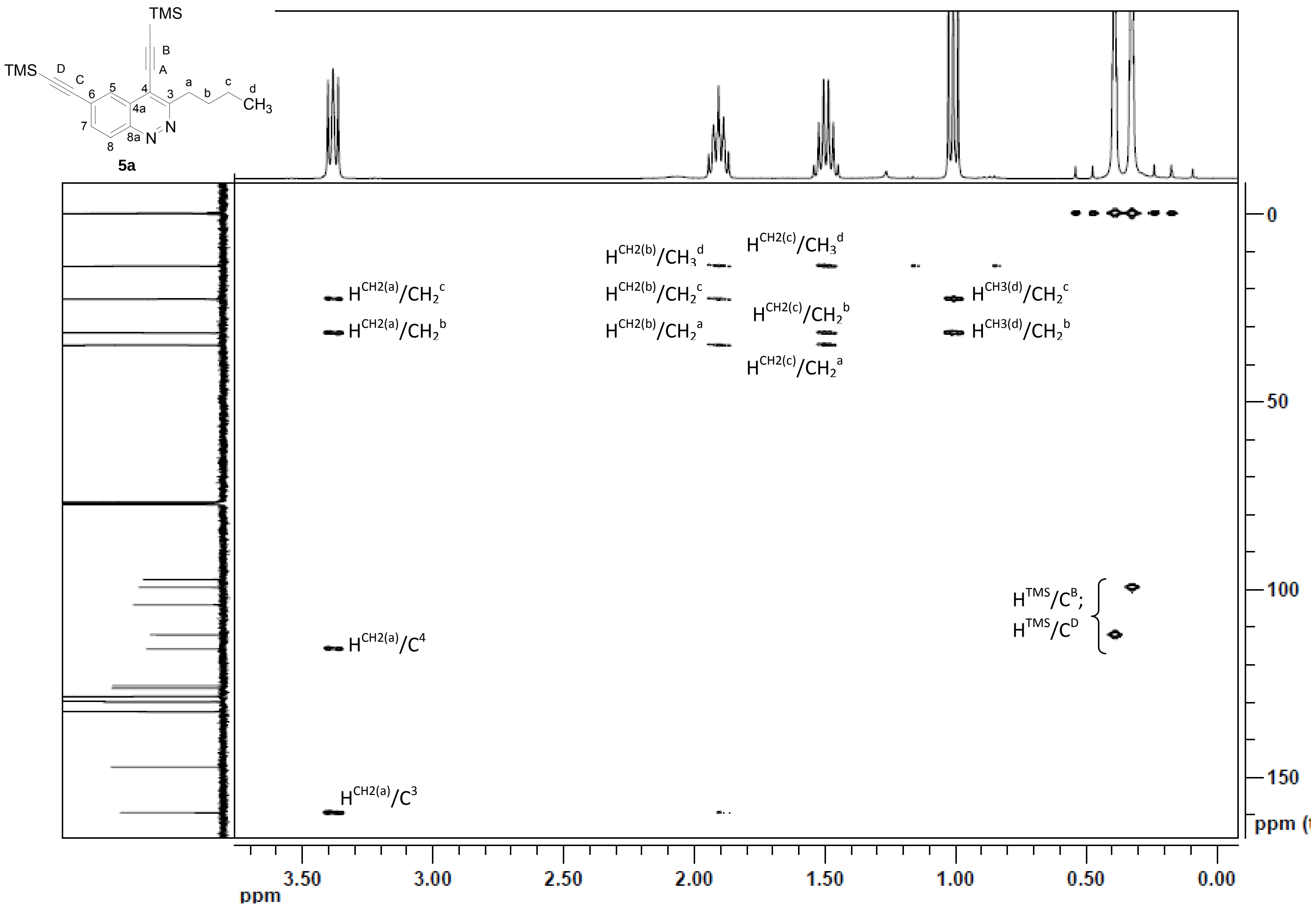

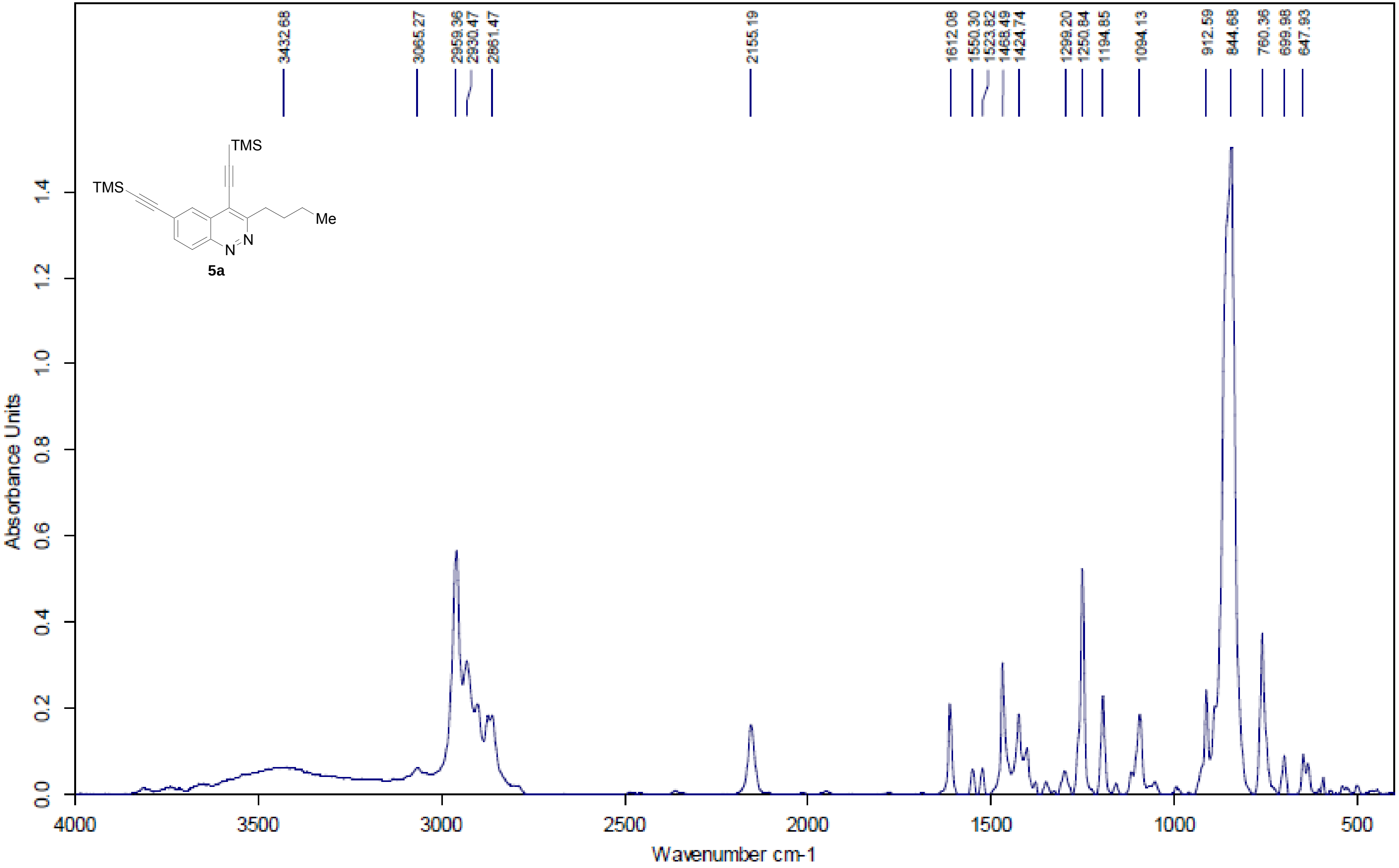
3432.68
3065.27
2959.36
2930.47
2861.47
2155.19
1612.08
1550.30
1523.82
1468.49
1424.74
1299.20
1250.84
1194.85
1094.13
912.59
844.68
760.36
699.98
647.93
TMS
TMS
Me
N
N
5a
Absorbance Units
0.0
0.2
0.4
0.6
0.8
1.0
1.2
1.4
4000
3500
3000
2500
2000
1500
1000
500
Wavenumber cm-1

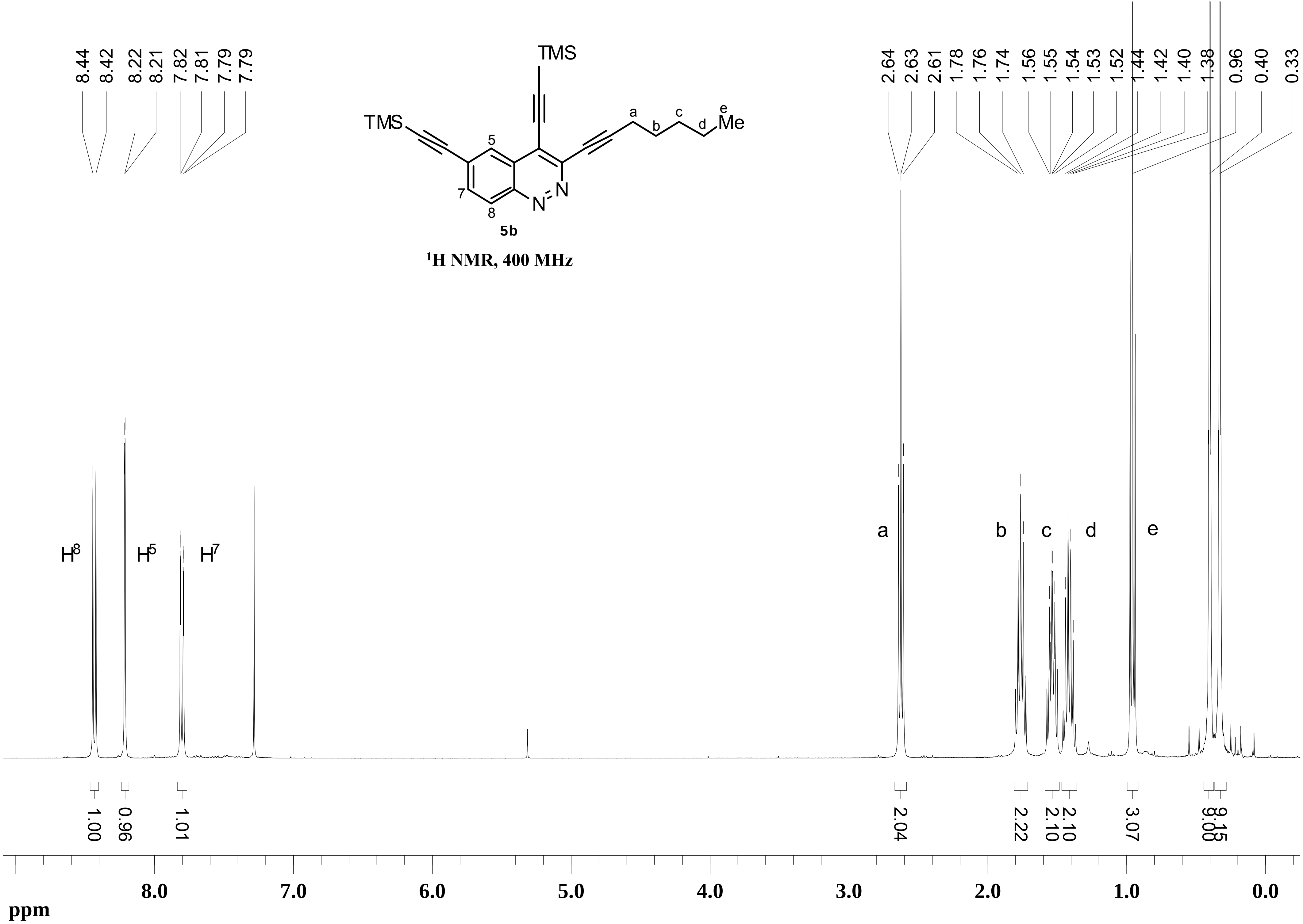

TMS
TMS
5b
1H NMR, 400 MHz
8.44 8.42 8.22 8.21 7.82 7.81 7.79 7.79
2.64 2.63 2.61 1.78 1.76 1.74 1.56 1.55 1.54 1.53 1.52 1.44 1.42 1.40 1.38 0.96 0.40 0.33
H8 H5 H7 a b c d e
1.00 0.96 1.01 2.04 2.22 2.10 2.10 3.07 9.00 9.15
8.0 7.0 6.0 5.0 4.0 3.0 2.0 1.0 0.0
ppm

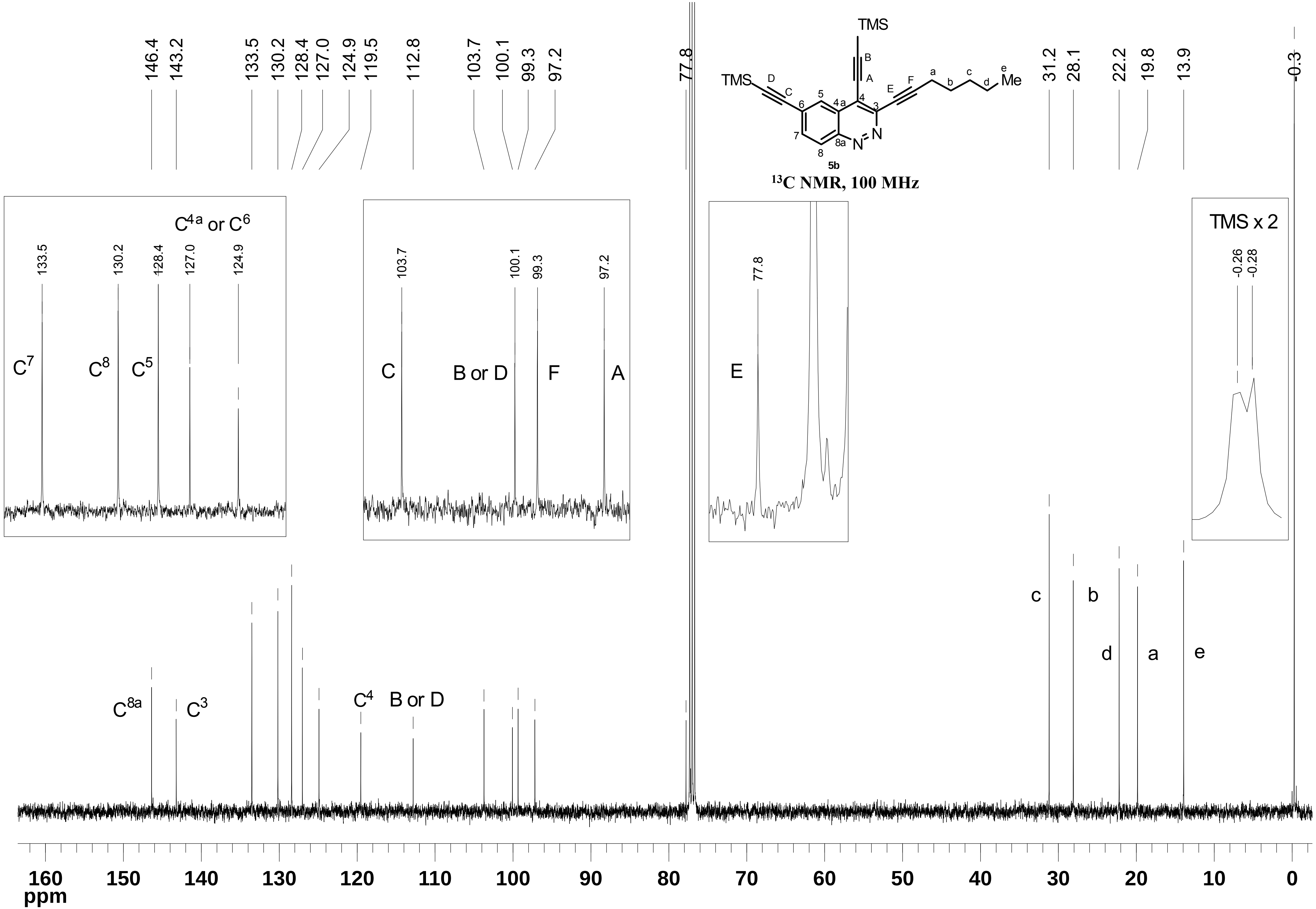
146.4
143.2
133.5
130.2
128.4
127.0
124.9
119.5
112.8
103.7
100.1
99.3
97.2
77.8
31.2
28.1
22.2
19.8
13.9
-0.3
TMS
B
A
TMS
D
C
E
F
a
b
c
d
e
Me
6
5
4a
4
3
7
8
8a
N
N
5b
13C NMR, 100 MHz
C4a or C6
C7
C8
C5
C
B or D
F
A
E
TMS x 2
-0.26
-0.28
c
b
d
a
e
C8a
C3
C4
B or D
160
150
140
130
120
110
100
90
80
70
60
50
40
30
20
10
0
ppm

HMBC NMR

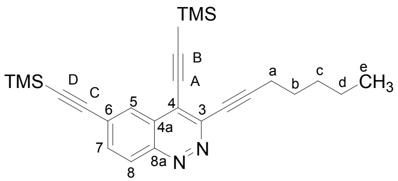
TMS
TMS
D
C
B
A
a
b
c
d
e
CH_3
6
5
4
4a
3
7
8
8a
N
N

5b

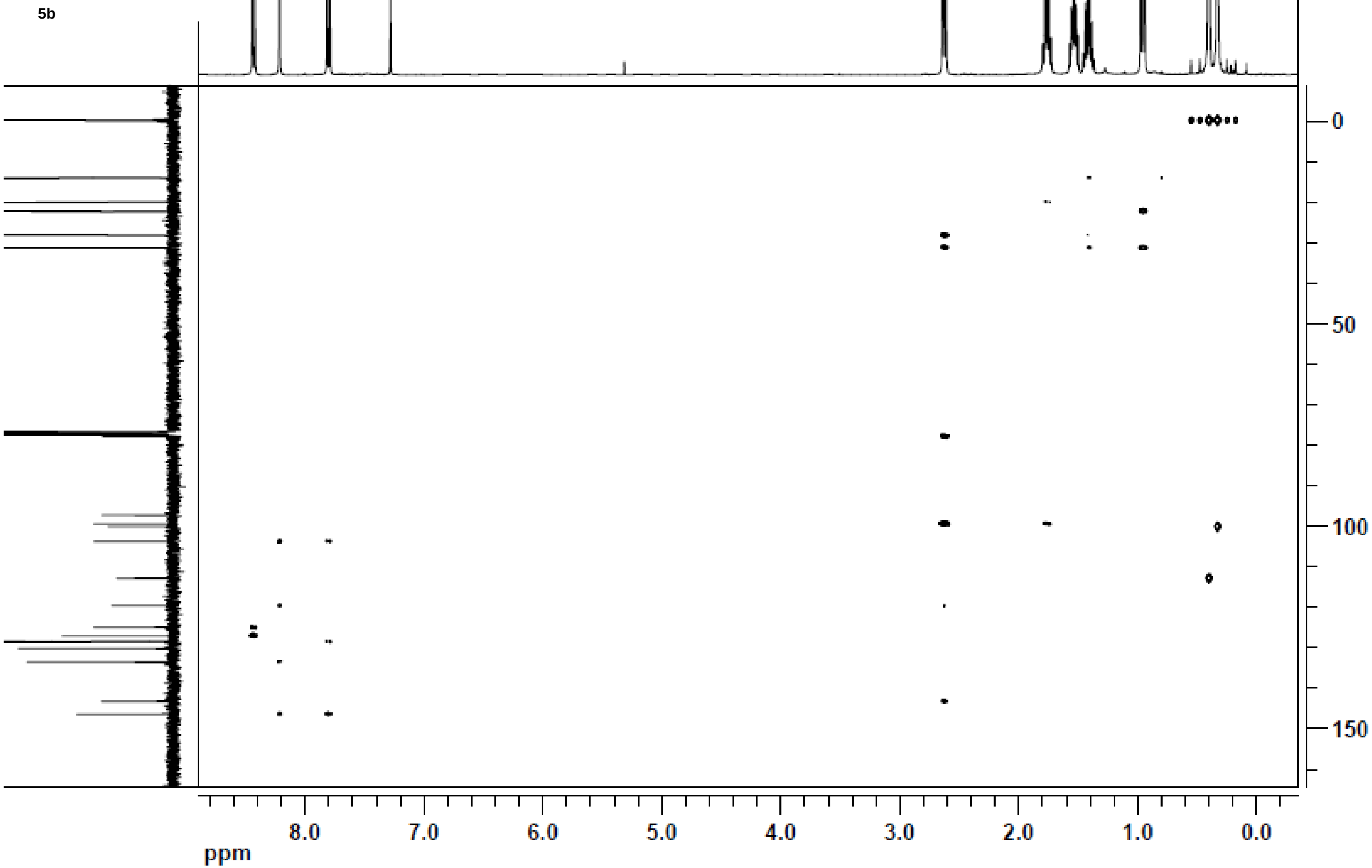
0
50
100
150
8.0
7.0
6.0
5.0
4.0
3.0
2.0
1.0
0.0
ppm

HMBC NMR (Zoom 1)

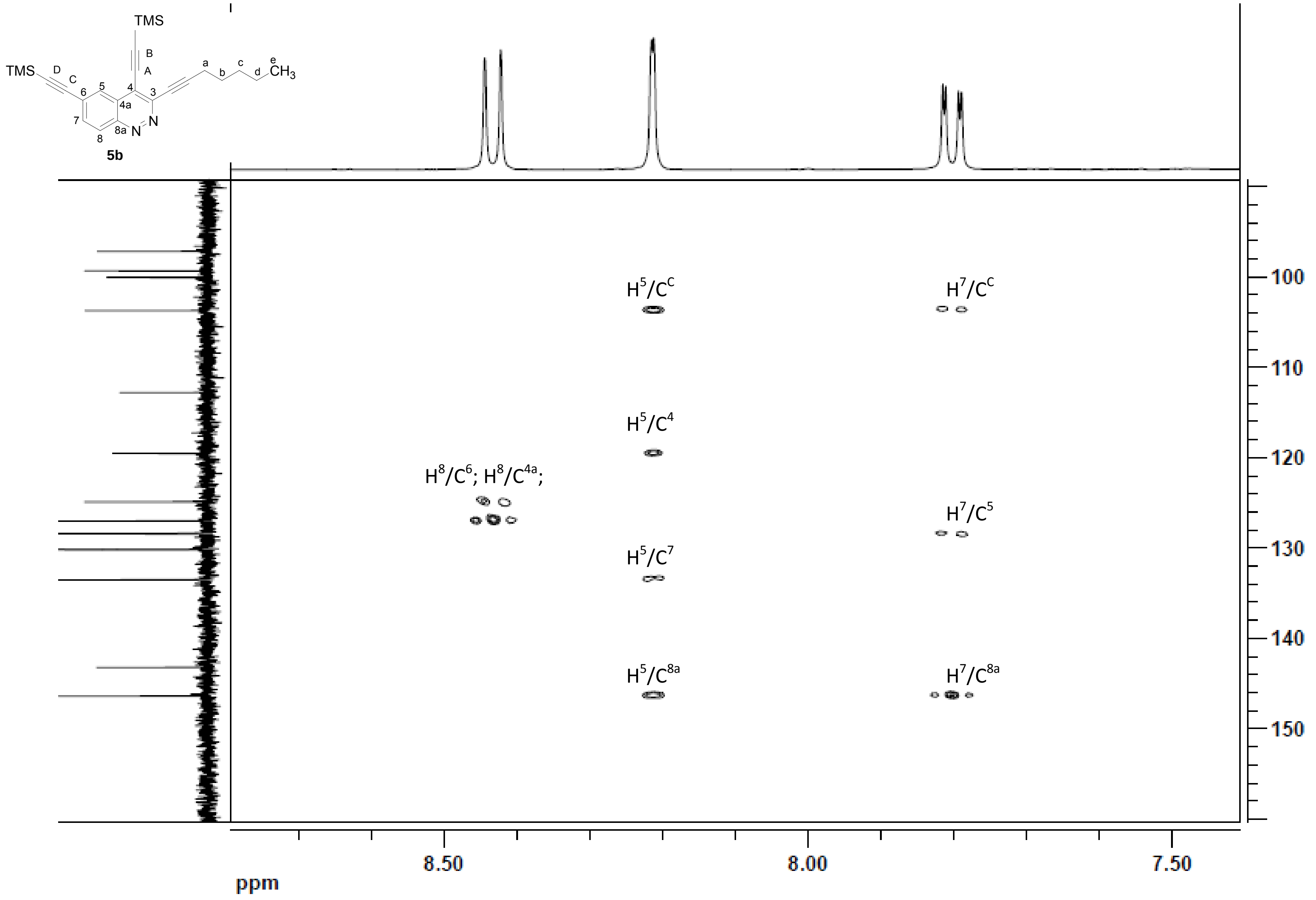

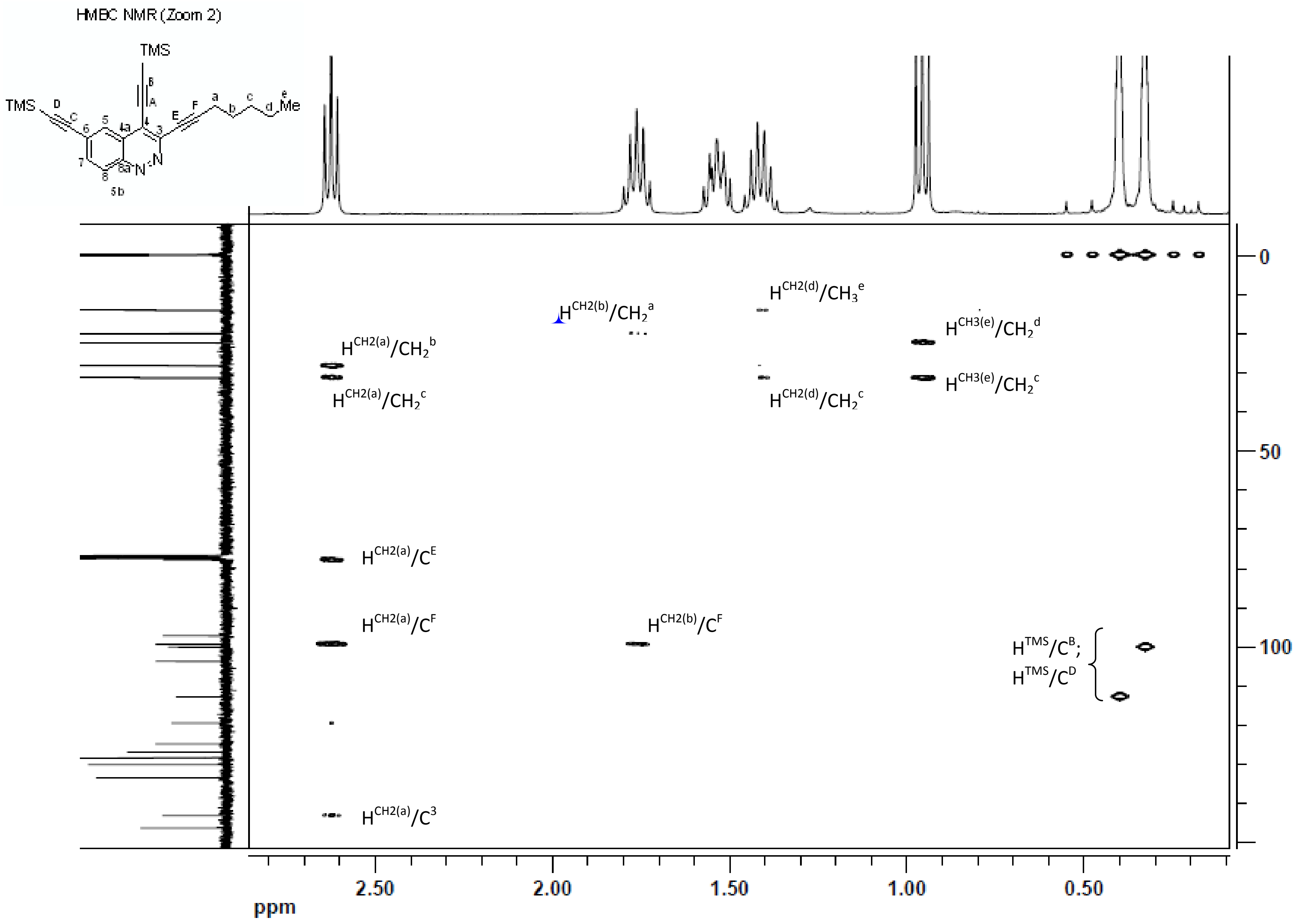
HMBC NMR (Zoom 2)
TMS
TMS
Me
H^CH2(b)/CH2^a
H^CH2(d)/CH3^e
H^CH3(e)/CH2^d
H^CH2(a)/CH2^b
H^CH2(a)/CH2^c
H^CH2(d)/CH2^c
H^CH3(e)/CH2^c
H^CH2(a)/C^E
H^CH2(a)/C^F
H^CH2(b)/C^F
H^TMS/C^B;
H^TMS/C^D
H^CH2(a)/C^3
0
50
100
2.50
2.00
1.50
1.00
0.50
ppm

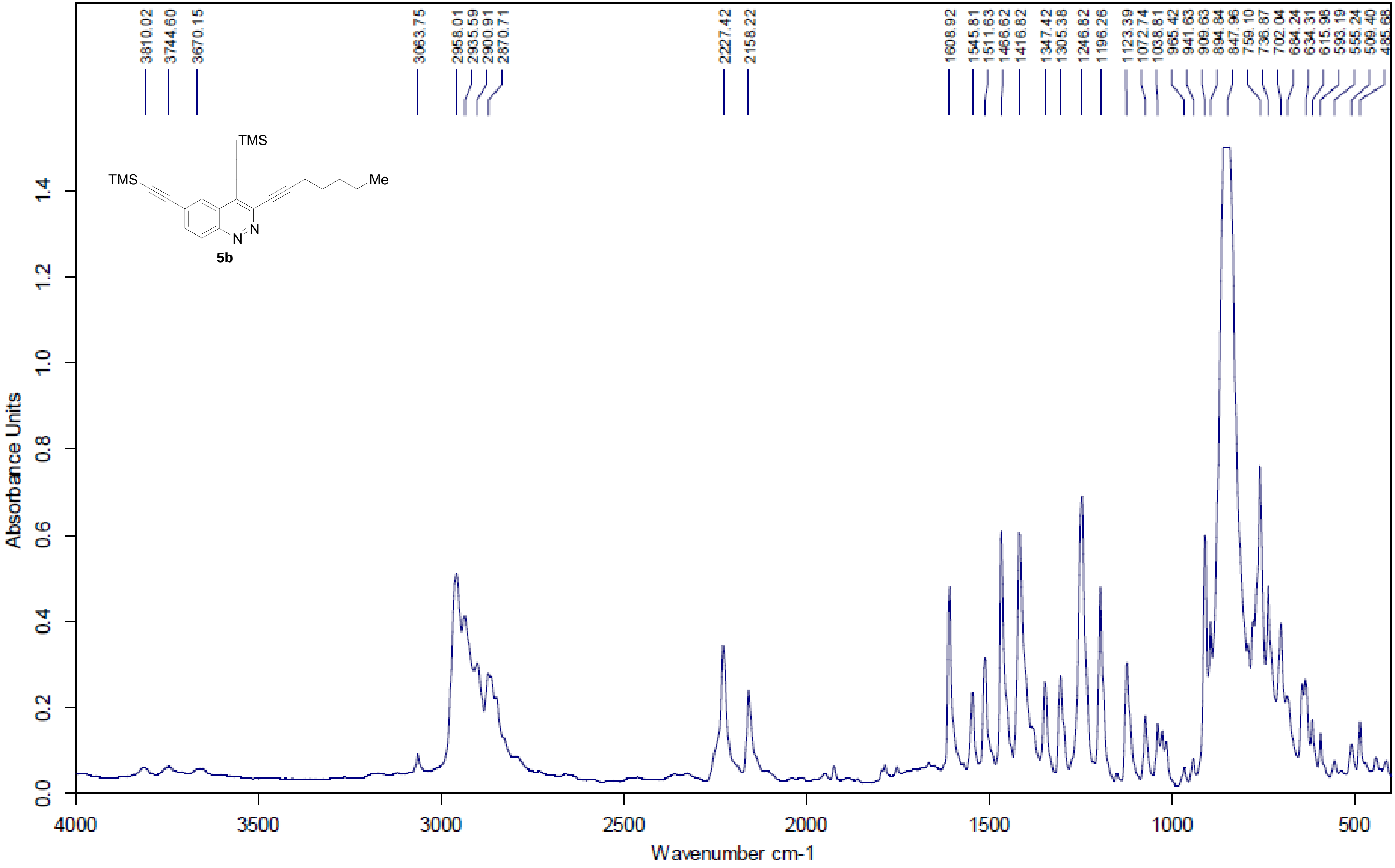
3810.02
3744.60
3670.15
3063.75
2958.01
2935.59
2900.91
2870.71
2227.42
2158.22
1608.92
1545.81
1511.63
1466.62
1416.82
1347.42
1305.38
1246.82
1196.26
1123.39
1072.74
1038.81
965.42
941.63
909.63
894.84
847.96
759.10
736.87
702.04
684.24
634.31
615.98
593.19
555.24
509.40
485.68
TMS
TMS
Me
N
N
5b
Absorbance Units
Wavenumber cm-1

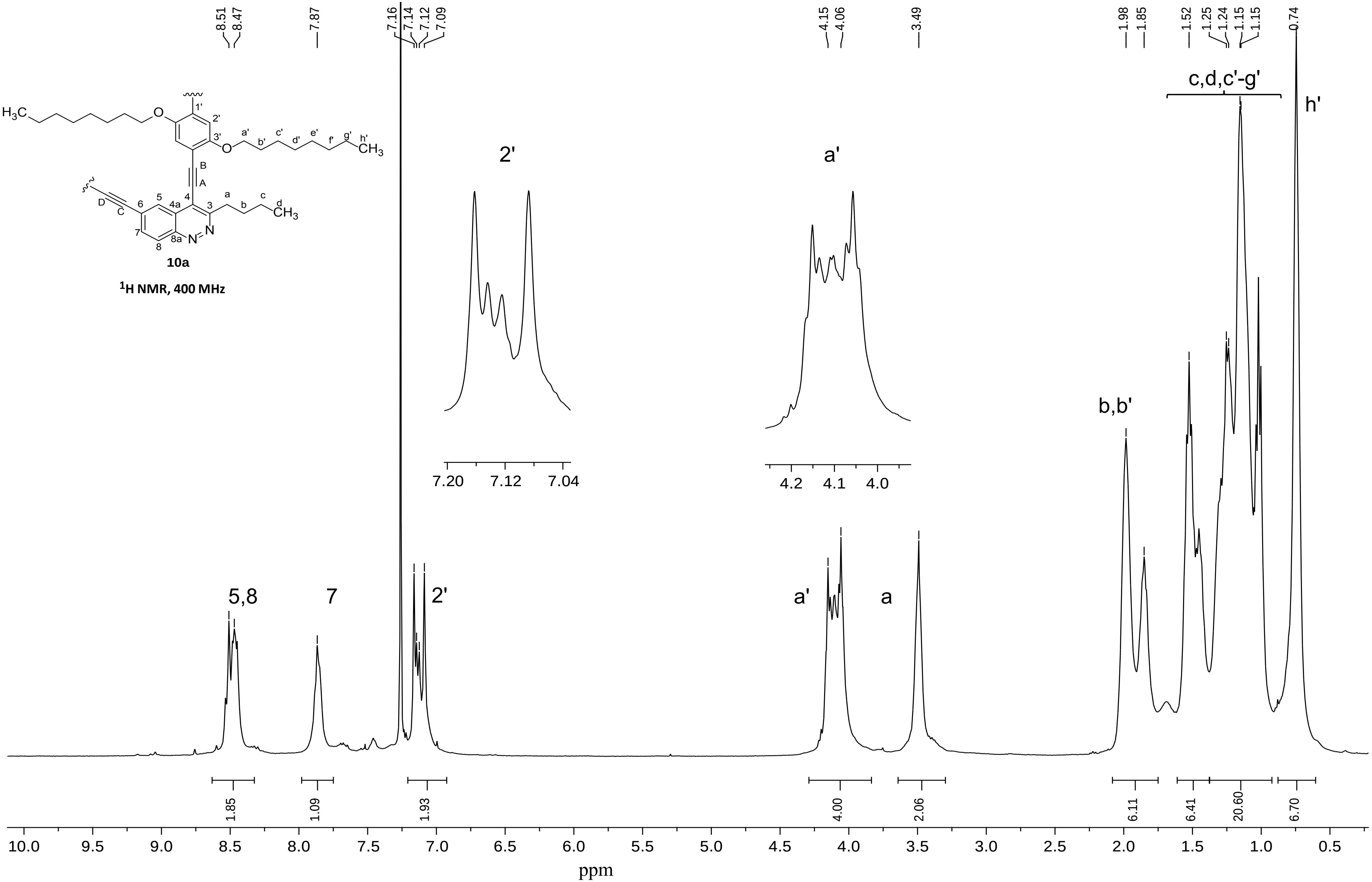
10a
1H NMR, 400 MHz
5,8
7
2'
a'
a
b,b'
c,d,c'-g'
h'
ppm

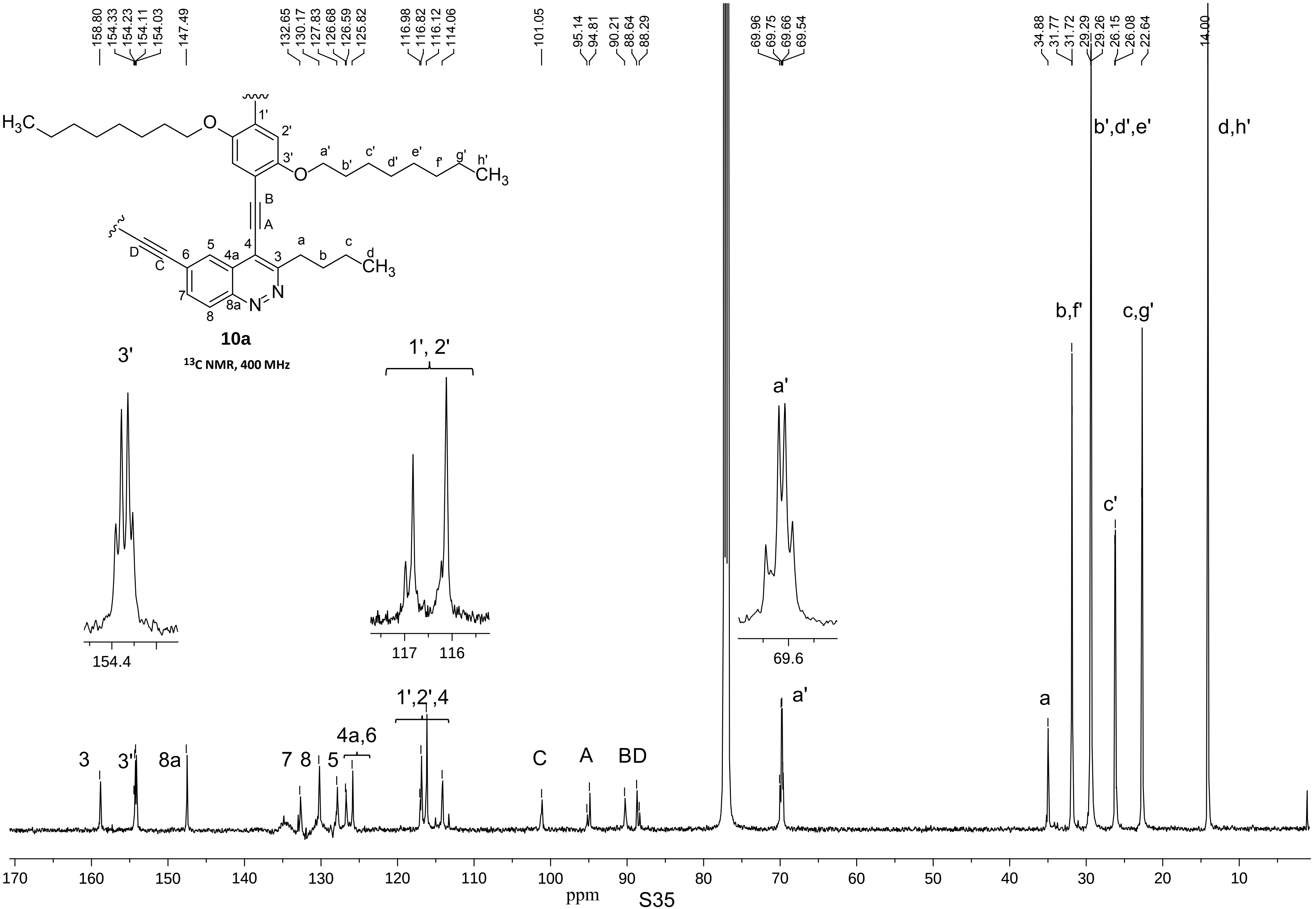
158.80
154.33
154.23
154.11
154.03
147.49
132.65
130.17
127.83
126.68
126.59
125.82
116.98
116.82
116.12
114.06
101.05
95.14
94.81
90.21
88.64
88.29
69.96
69.75
69.66
69.54
34.88
31.77
31.72
29.29
29.26
26.15
26.08
22.64
14.00
10a
13C NMR, 400 MHz
3'
1', 2'
a'
b',d',e'
d,h'
b,f'
c,g'
c'
a
3
8a
7 8
5
4a,6
1',2',4
C
A
BD
154.4
117
116
69.6
ppm

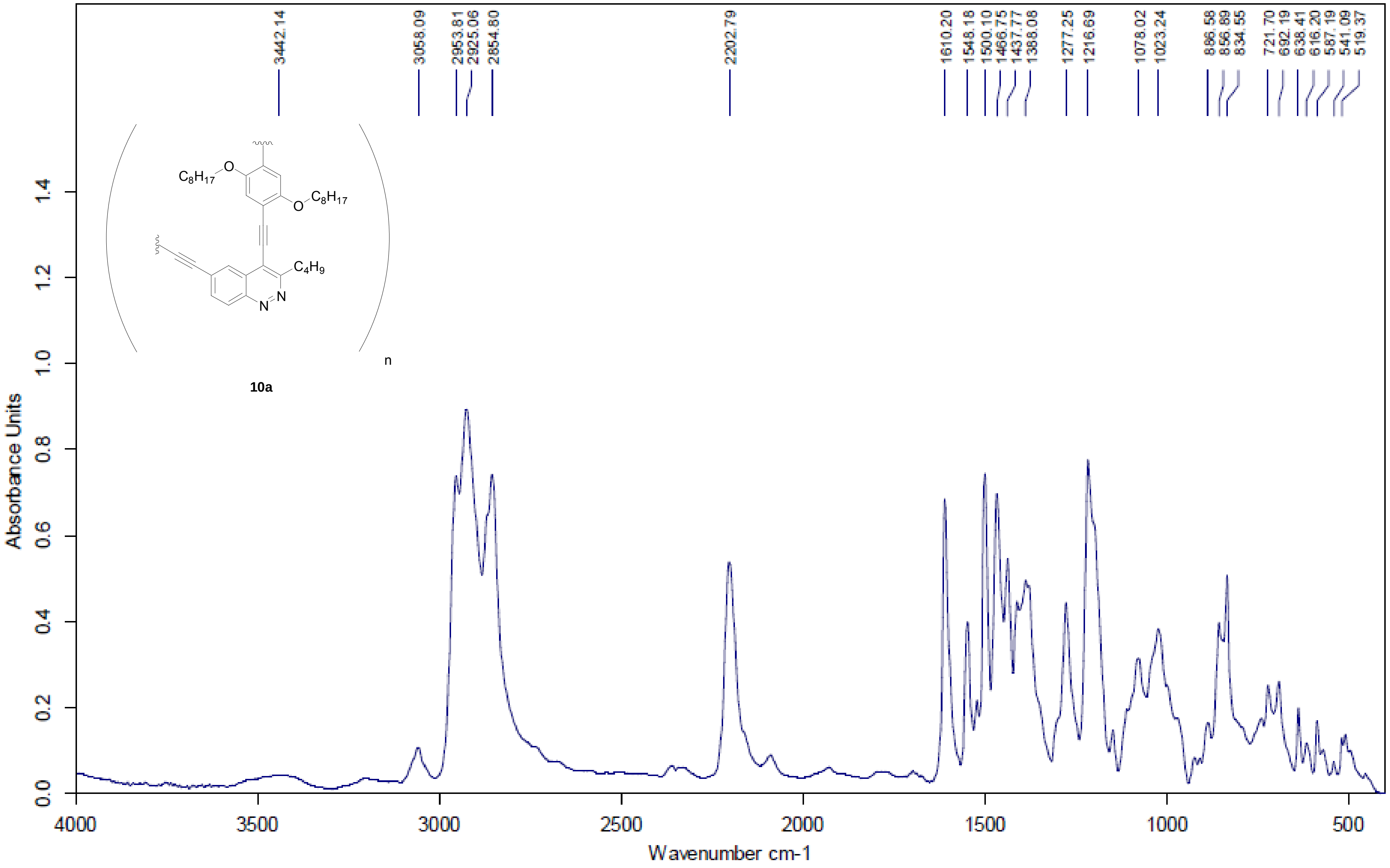
3442.14
3058.09
2953.81
2925.06
2854.80
2202.79
1610.20
1548.18
1500.10
1466.75
1437.77
1388.08
1277.25
1216.69
1078.02
1023.24
886.58
856.89
834.55
721.70
692.19
638.41
616.20
587.19
541.09
519.37
Absorbance Units
Wavenumber cm-1
10a

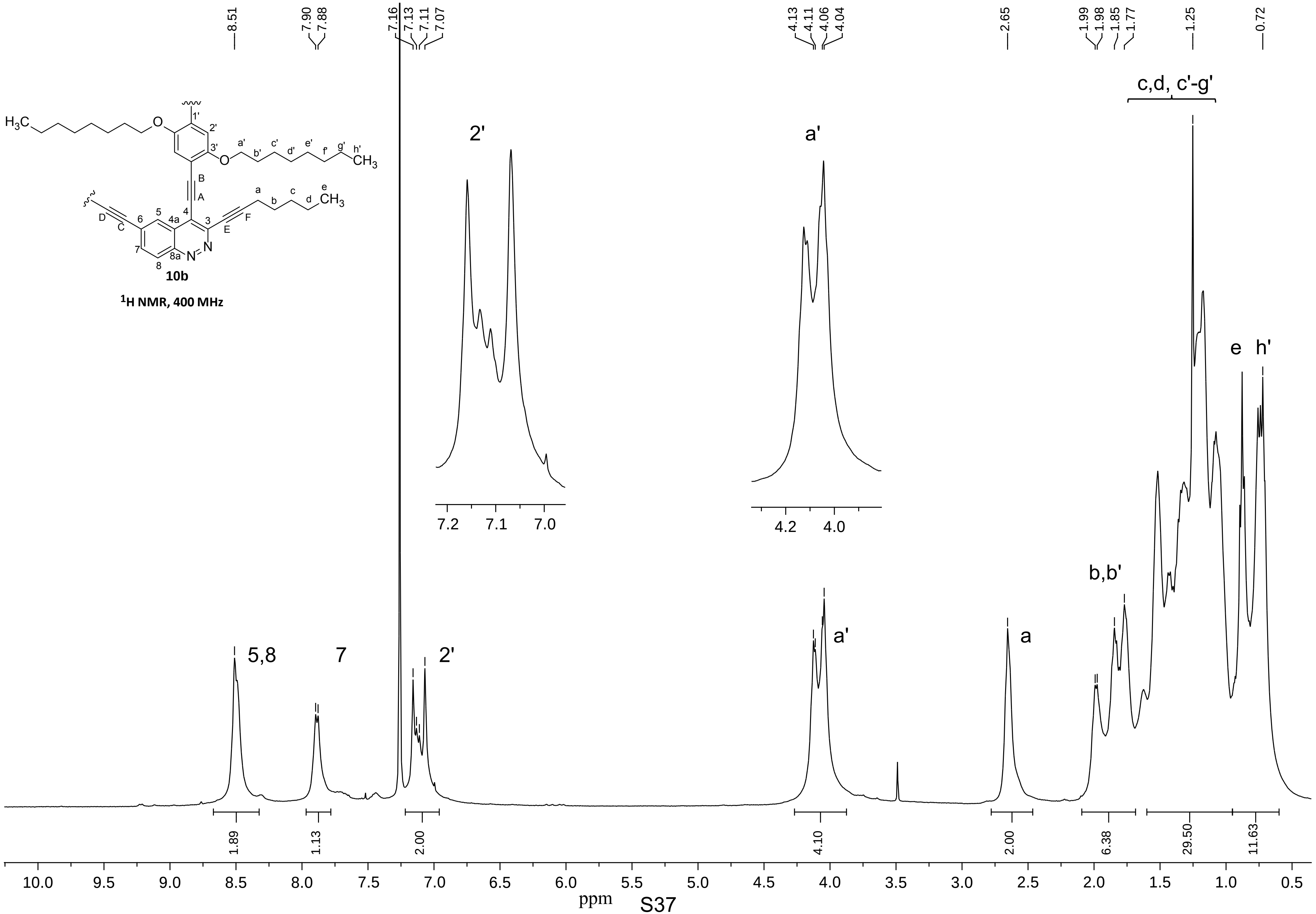

10b
1H NMR, 400 MHz
8.51
7.90
7.88
7.16
7.13
7.11
7.07
4.13
4.11
4.06
4.04
2.65
1.99
1.98
1.85
1.77
1.25
0.72
c,d, c'-g'
2'
a'
e
h'
b,b'
5,8
7
a
1.89
1.13
2.00
4.10
2.00
6.38
29.50
11.63
ppm

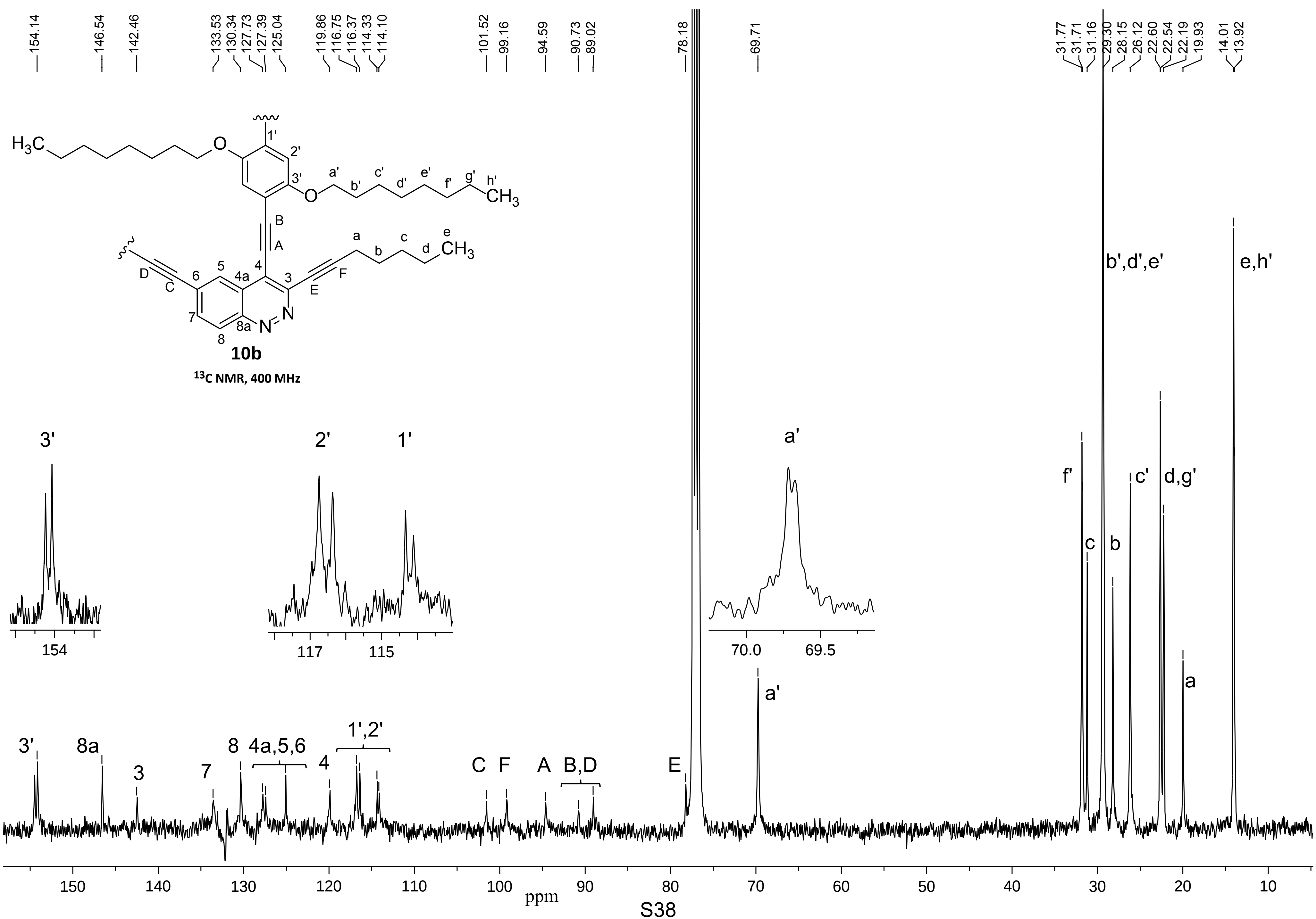
154.14
146.54
142.46
133.53
130.34
127.73
127.39
125.04
119.86
116.75
116.37
114.33
114.10
101.52
99.16
94.59
90.73
89.02
78.18
69.71
31.77
31.71
31.16
29.30
28.15
26.12
22.60
22.54
22.19
19.93
14.01
13.92
10b
13C NMR, 400 MHz
3'
2'
1'
a'
b',d',e'
e,h'
f'
c'
d,g'
c
b
a
8a
3
7
8
4a,5,6
4
1',2'
C
F
A
B,D
E
ppm

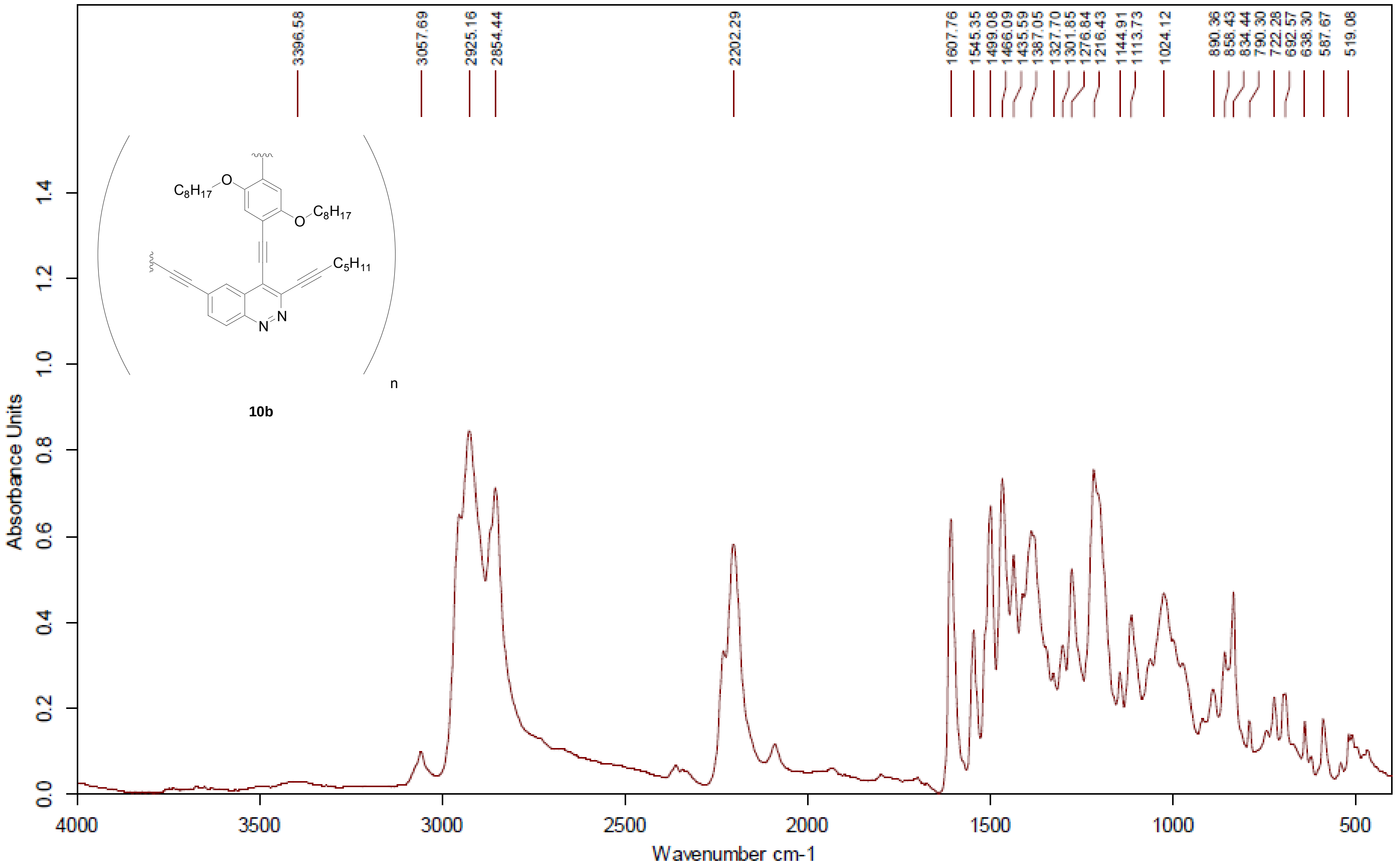
3396.58
3057.69
2925.16
2854.44
2202.29
1607.76
1545.35
1499.08
1466.09
1435.59
1387.05
1327.70
1301.85
1276.84
1216.43
1144.91
1113.73
1024.12
890.36
858.43
834.44
790.30
722.28
692.57
638.30
587.67
519.08
C_8H_{17}
O
C_8H_{17}
O
C_5H_{11}
N
N
n
10b
Absorbance Units
0.0
0.2
0.4
0.6
0.8
1.0
1.2
1.4
4000
3500
3000
2500
2000
1500
1000
500
Wavenumber cm-1

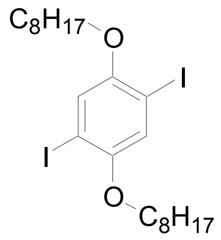

9

^{1}H NMR, 400 MHz

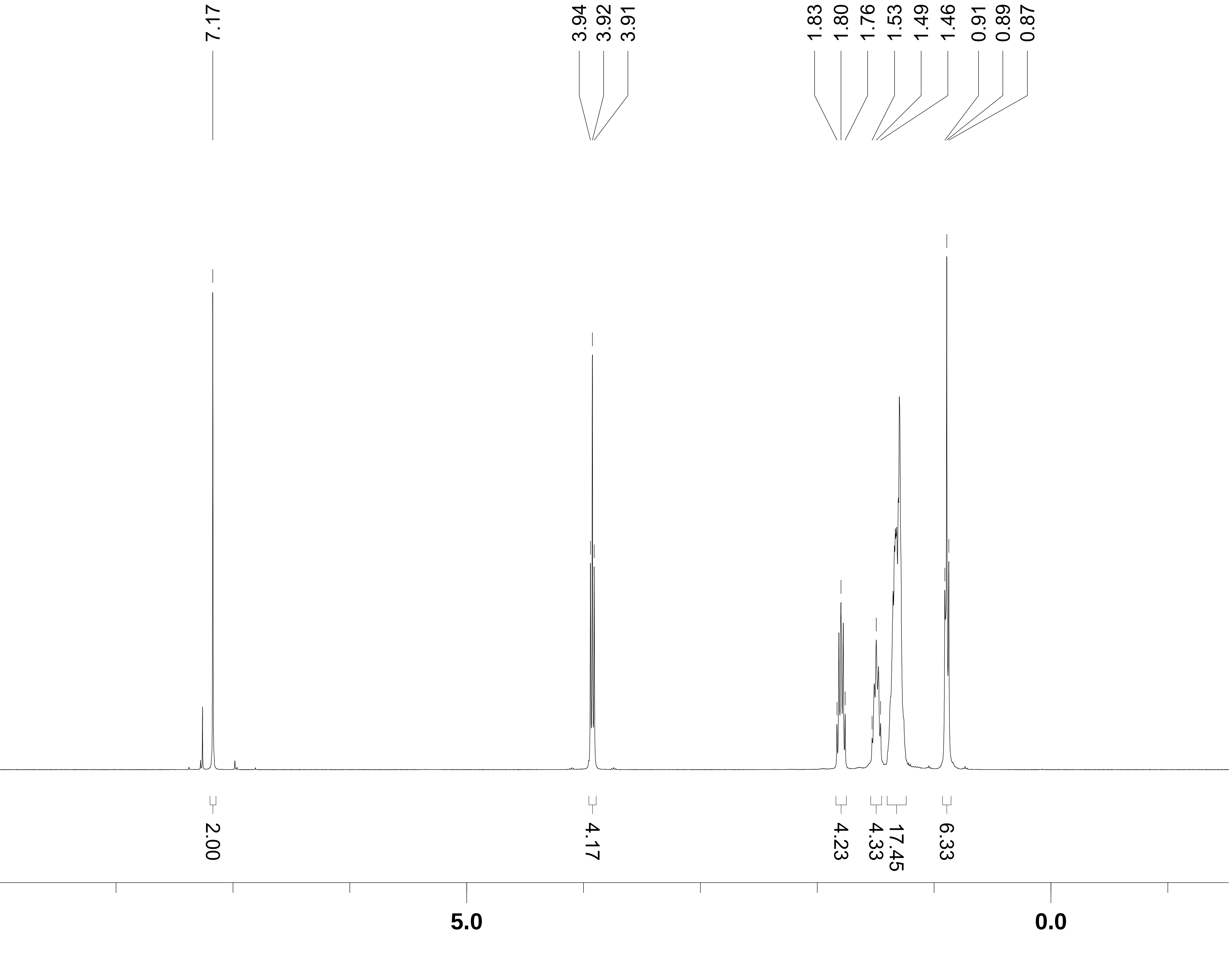

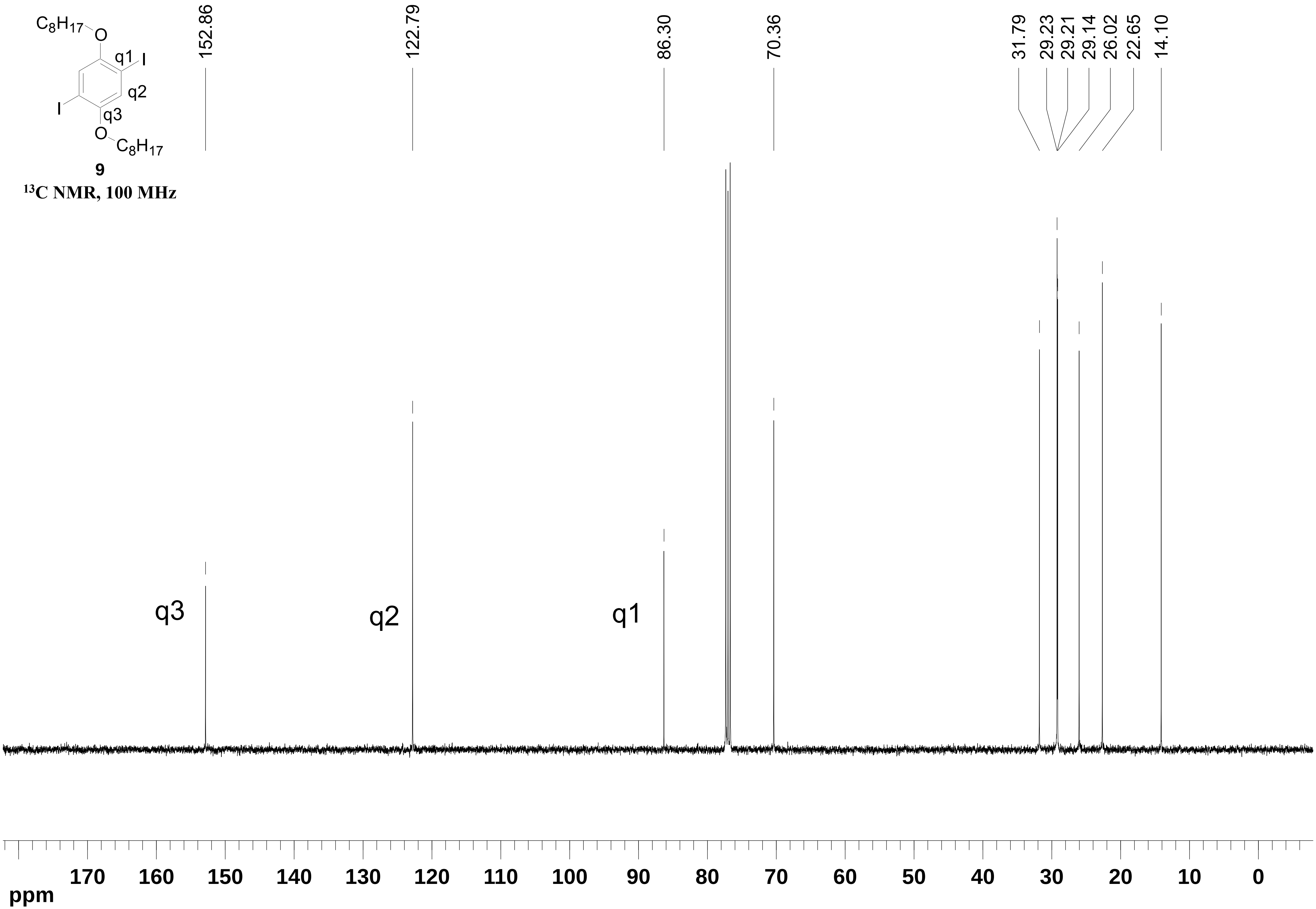

C_8H_{17}
O
q1
I
q2
I
q3
O
C_8H_{17}
9
^{13}C NMR, 100 MHz
152.86
122.79
86.30
70.36
31.79
29.23
29.21
29.14
26.02
22.65
14.10
q3
q2
q1
170
160
150
140
130
120
110
100
90
80
70
60
50
40
30
20
10
0
ppm

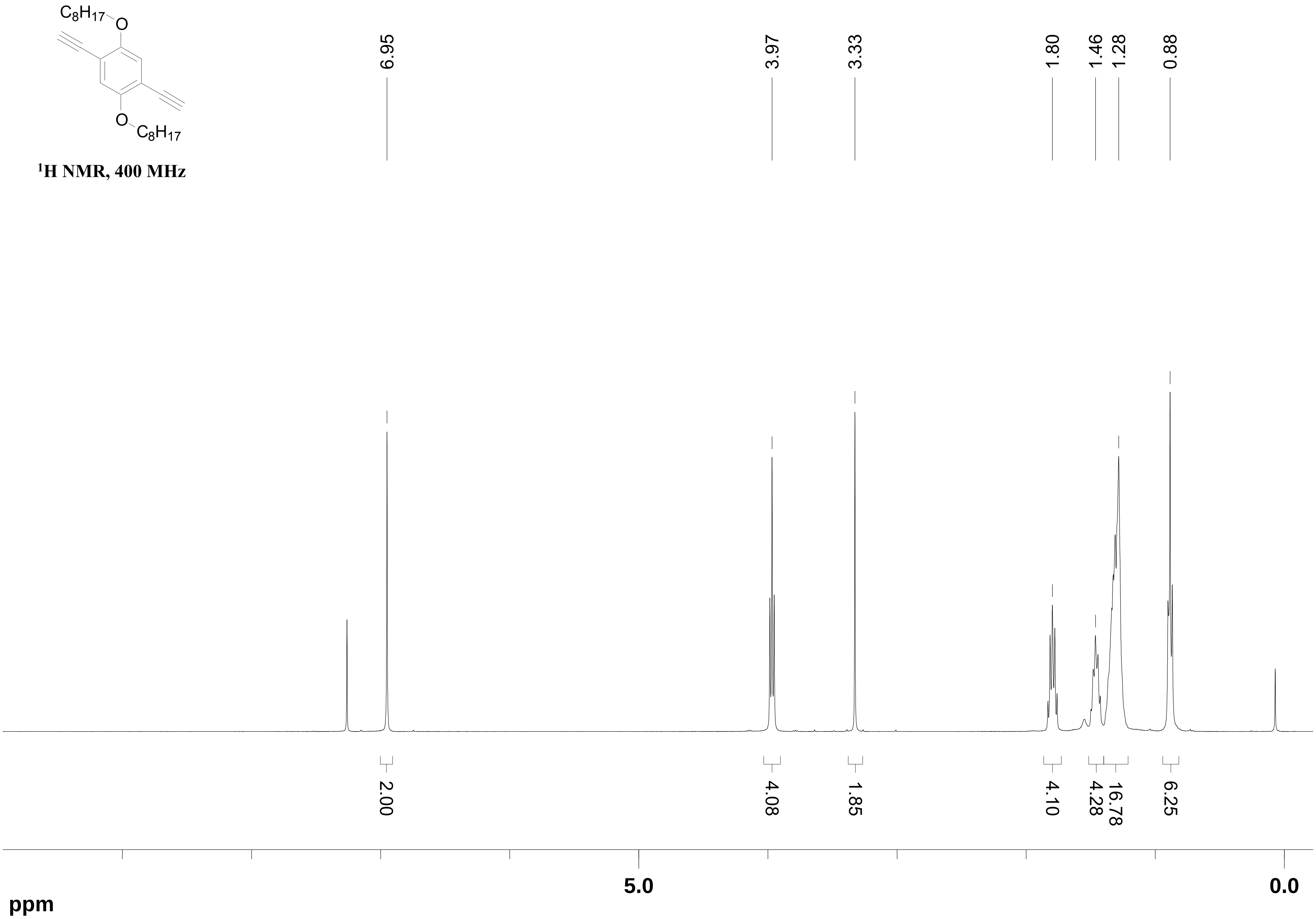

C_8H_{17}
O
O
C_8H_{17}
^{1}H NMR, 400 MHz
6.95
3.97
3.33
1.80
1.46
1.28
0.88
2.00
4.08
1.85
4.10
4.28
16.78
6.25
5.0
0.0
ppm

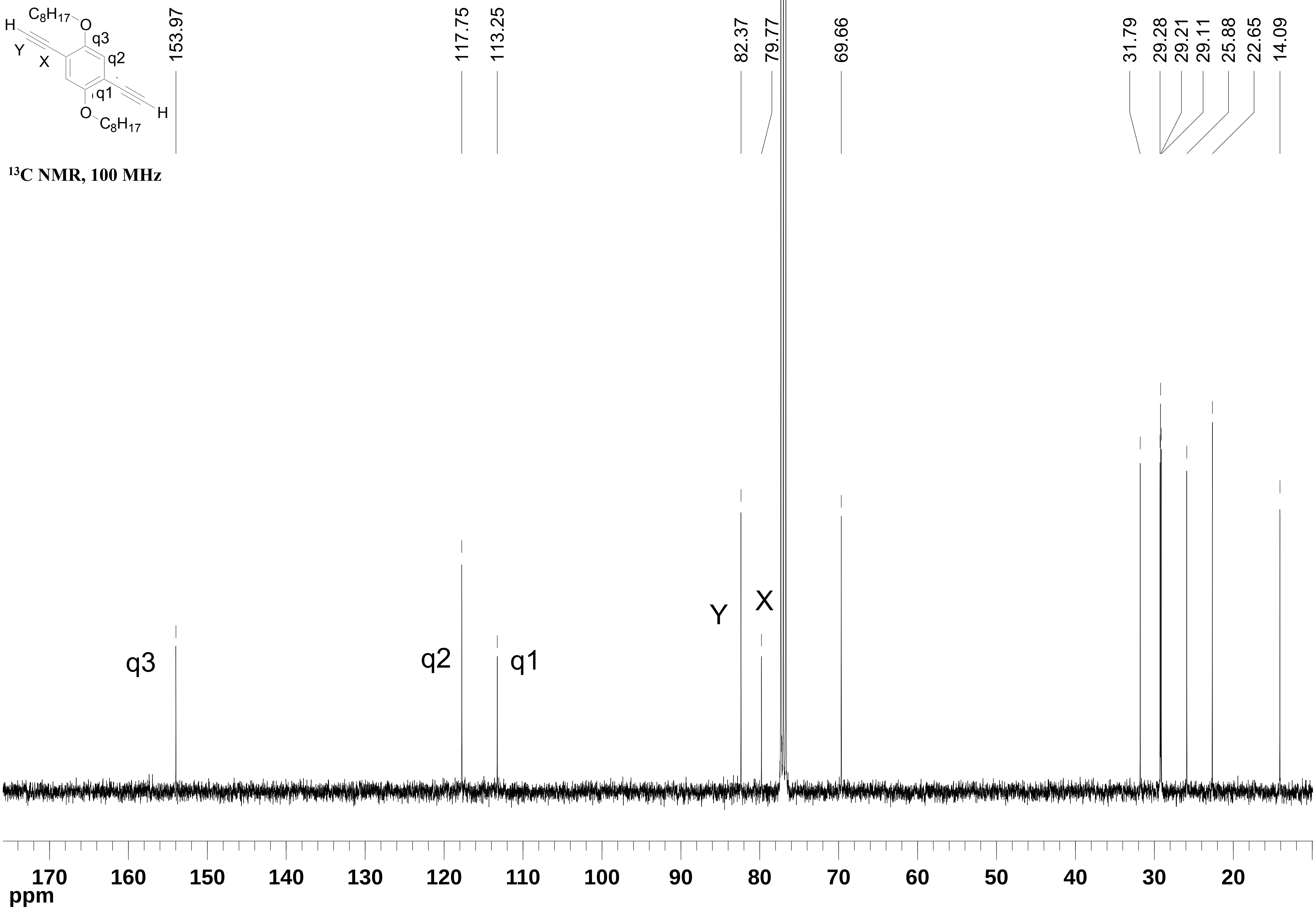

^{13}C NMR, 100 MHz

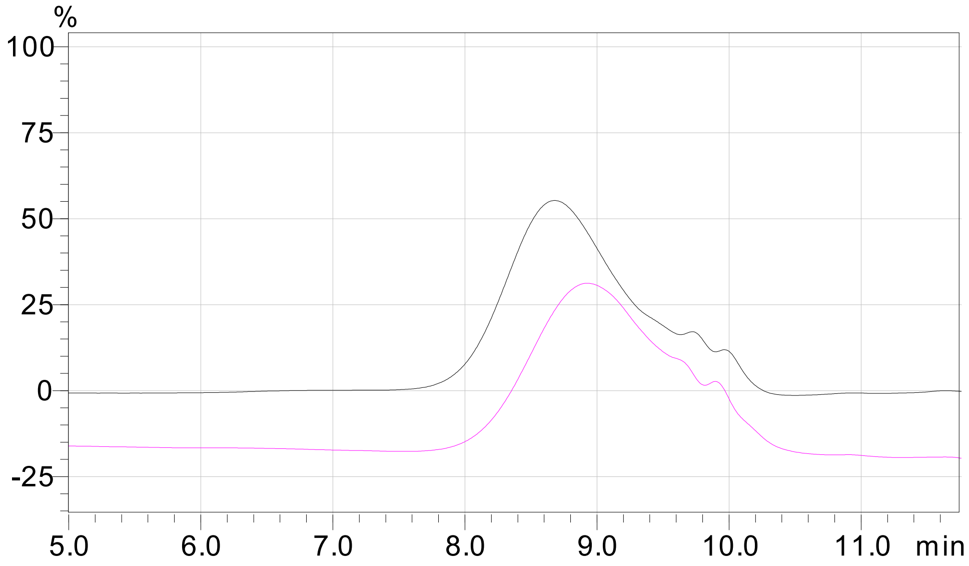

GPC analysis of PAEs **10a,b.** Elution curves for **10a** (black) and **10b** (pink) are shown

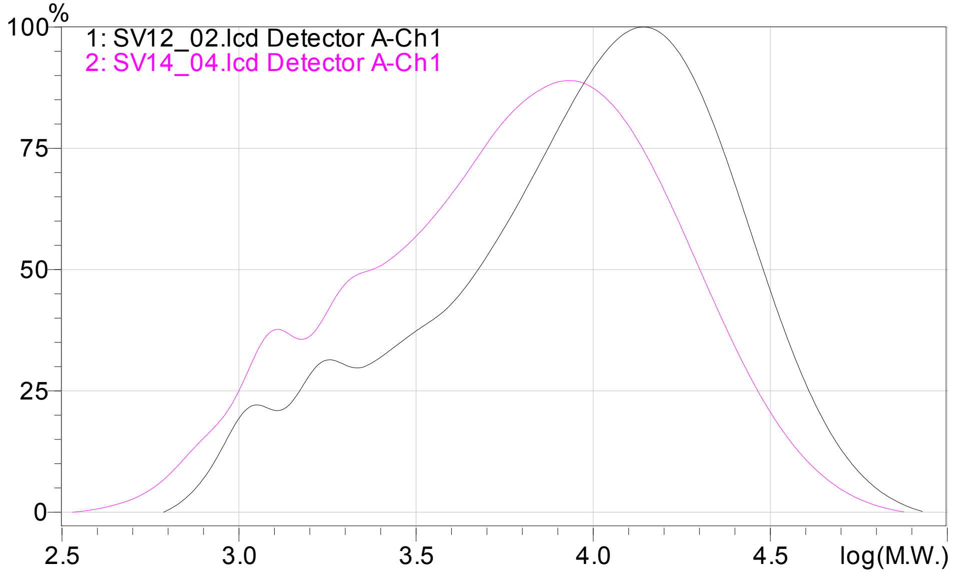

GPC analysis of PAEs **10a,b.** Mw distribution curves for **10a** (black) and **10b** (pink) are shown.
